# The poly(I:C)-induced maternal immune activation model; a systematic review and meta-analysis of cytokine levels in the offspring

**DOI:** 10.1016/j.bbih.2020.100192

**Published:** 2020-12-19

**Authors:** Bart C. Hameete, José M.S. Fernández-Calleja, Martje W.G.D.M. de Groot, Titia Rixt Oppewal, Machteld M. Tiemessen, Astrid Hogenkamp, Rob B.M. de Vries, Lucianne Groenink

**Affiliations:** a Department of Pharmacology, Utrecht Institute for Pharmaceutical Sciences (UIPS), Utrecht University, Universiteitsweg 99, Utrecht, 3584 CG, the Netherlands; b University College Utrecht (UCU), Campusplein 1, Utrecht, 3584 ED, the Netherlands; c Stratingh Institute for Chemistry, University of Groningen, Nijenborgh 4, Groningen, 9747 AG, the Netherlands; d Research & Innovation, GCoE Immunology, Danone Nutricia Research, Uppsalalaan 12, Utrecht, 3584 CT, the Netherlands; e SYstematic Review Center for Laboratory (Animal) Experimentation, Department for Health Evidence, Radboud University Medical Center, Geert Grooteplein zuid 10, Nijmegen, 6525 GA, the Netherlands

**Keywords:** Poly I–C, Maternal exposure, Cytokines, Systematic review, Meta-analysis, Immune system, Neurodevelopmental disorders, Models animal, Maternal immune activation

## Abstract

The maternal polyinosinic:polycytidylic acid (poly(I:C)) animal model is frequently used to study how maternal immune activation may impact neuro development in the offspring. Here, we present the first systematic review and meta-analysis on the effects of maternal poly(I:C) injection on immune mediators in the offspring and provide an openly accessible systematic map of the data including methodological characteristics.

Pubmed and EMBASE were searched for relevant publications, yielding 45 unique papers that met inclusion criteria. We extracted data on immune outcomes and methodological characteristics, and assessed the risk of bias. The descriptive summary showed that most studies reported an absence of effect, with an equal number of studies reporting an increase or decrease in the immune mediator being studied.

Meta-analysis showed increased IL-6 concentrations in the offspring of poly(I:C) exposed mothers. This effect appeared larger prenatally than post-weaning. Furthermore, poly(I:C) administration during mid-gestation was associated with higher IL-6 concentrations in the offspring. Maternal poly(I:C) induced changes in IL-1β, Il-10 and TNF-α concentrations were small and could not be associated with age of offspring, gestational period or sampling location. Finally, quality of reporting of potential measures to minimize bias was low, which stresses the importance of adherence to publication guidelines.

Since neurodevelopmental disorders in humans tend to be associated with lifelong changes in cytokine concentrations, the absence of these effects as identified in this systematic review may suggest that combining the model with other etiological factors in future studies may provide further insight in the mechanisms through which maternal immune activation affects neurodevelopment.

## Highlights

• Long-term effects of maternal poly(I:C) on immune mediators in the offspring appear limited.
• Prenatal measurements and mid gestation poly(I:C) injection are associated with increases in IL-6 concentrations.
• Variety in methodological conduct hampers identification of key elements that affect cytokine concentrations.
• The quality of reporting of potential measures to minimize bias is poor.

## 1 Introduction

It was observed as early as 1985 that the seasonal birthrates of people suffering from schizophrenia differ from those of the general population (Bradbury and Miller, 1985). Those born during the winter and spring have an increased risk of schizophrenia and other psychiatric disorders, suggesting the existence of risk factors for neurodevelopmental deficits that correlate with seasonal change (Torrey et al., 1997). One popular contender was infectious agents, which are known for showing seasonal variance. Indeed, a wide variety of infectious diseases have been linked to neurodevelopmental disorders, including but not limited to, influenza, *Toxoplasma gondii*, rubella, herpes simplex type 2 and infection in general (Brown et al., 2000; Brown et al., 2004; Brown et al., 2005; (Brown et al., 2014; Canetta et al., 2014; Parboosing et al., 2013; Mortensen et al., 2007; Mortensen et al., 2010). Because this connection did not appear to be limited to any specific pathogen, it has instead been hypothesized that the maternal immune response to the pathogen, or “maternal immune activation”, is the driving force behind the detrimental effects on neurodevelopment in the offspring (Solek et al., 2018).

While a relationship has been established between maternal immune activation and neurodevelopmental disorders using epidemiological studies, such studies are poorly suited to study the mechanisms that accommodate this link. There are several potential mechanisms through which maternal immune activation could influence fetal neurodevelopment, each of which may be fully or partially responsible for the increased risk for neurodevelopmental disorders. Since neurodevelopmental disorders are typically difficult to treat, understanding their etiology and working towards a form of prevention is very worthwhile. One possible mechanism through which maternal immune activation may influence fetal development could be the alteration of cytokine concentrations in the fetus, as cytokines are well known to play a role in the functioning and development of both the nervous and immune system (Deverman and Patterson, 2009; McAfoose and Baune, 2009). However, substantial *in vitro* and *in vivo* work is necessary to come to a full understanding of the mechanisms through which maternal immune activation acts as a risk factor for neurodevelopmental disorders.

For this purpose, several animal models for maternal immune activation have been developed, using live pathogens or immunogens such as lipopolysaccharide. One of the best established maternal immune activation models however is the maternal polyinosinic:polycytidylic acid (poly(I:C)) model, which uses a synthetic analogue of double-stranded RNA, poly(I:C), to mimic a viral infection during pregnancy. The poly(I:C) model is considered to have good construct, face and predictive validity. Poly(I:C) binds to the toll-like receptor 3, retinoic-acid-inducible protein 1 and melanoma-differentiation-associated gene 5, which results in the release of pro-inflammatory cytokines and initiates the inflammatory cascade in a fashion similar to a viral infection (Linehan et al., 2018, Takeuchi and Akira, 2007). In addition, like prenatal infection, injection with poly(I:C) has been shown to cause a multitude of behavioral and cognitive deficits in the offspring that can be alleviated by antipsychotic drug treatment (Ozawa et al., 2006; Zuckerman et al., 2003; Zuckerman and Weiner, 2005).

While the maternal poly(I:C) model has been used to study the effect of maternal immune activation on neurodevelopment and behavior for over fifteen years, a systematic review (SR) and meta-analysis (MA) that objectively summarizes and synthesizes the currently available, often conflicting findings has not yet been performed. Given the presumed role of immune mediators in the detrimental effects of maternal immune activation, a first step towards a better understanding of how neurodevelopment may be affected is comparing the immunological profiles of offspring from poly(I:C)-treated pregnant animals to those of the offspring of vehicle-treated pregnant animals.

Since there is a large variability in experimental set-up of animal studies, this meta-analysis has an exploratory purpose. The analyses mainly focus on evaluating heterogeneity between studies and establishing the relationship between study characteristics and the direction of effects induced by maternal poly(I:C), rather than focusing on the pooled effect sizes per se. Results may be used to generate new hypotheses and guide the design of future studies.

The current systematic review was performed by collecting all relevant papers through a search in electronic scientific databases. We proceeded by inventorying study characteristics, assessing study quality and extracting immune outcomes. When possible, these outcomes were further analyzed and visualized. Given the anticipated differences in outcome measurements and methodological characteristics we used standardized mean differences, a random effects model and subgroup analysis to study factors influencing the effect of maternal poly(I:C) in the offspring. Predefined factors for subgroup analyses were species, the gestational period during which poly(I:C) was injected, age of offspring at outcome assessment and sampling location. Together, this provided a comprehensive summary and analysis of the available evidence, which may help decide on methods and directions for future research.

## 2 Materials and Methods

This systematic review was performed based on a preregistered protocol (CAMARADES, 2014). After the initial search the scope of the review was limited to changes in cytokines in the offspring and accompanying changes were then documented in the SYRCLE protocol (Supplementary file S1). No changes to the protocol were made after beginning the extraction phase.

a. Exclusion criteria: to reduce heterogeneity, studies involving pre- or postnatal comorbidities (i.e. double hit models), studies involving treatments to prevent or reduce the negative impact of poly(I:C) on the offspring (i.e. co-treatments), and studies involving genetically modified organisms (GMO) were excluded, unless these studies also contained groups that met the inclusion criteria.
b. Risk of bias assessment: the SYstematic Review Centre for Laboratory animal Experimentation (SYRCLE) risk of bias tool was used instead of the more limited criteria formulated in the preregistered protocol (Hooijmans et al., 2014).
c. Data analysis/synthesis: the meta-analysis was limited to outcome measures reported in at least 10 independent studies to ensure sufficient statistical power and translatability.

### 2.1 Literature search and selection

#### 2.1.1 Search strategy

A literature search in two major biomedical databases, PubMed and EMBASE, was completed including all papers published up until August 6, 2019. The search strategy was based on the search components “poly(I:C)”, “maternal exposure” and “animal model”. A full search string can be found in supplementary file S1.

#### 2.1.2 Study selection

Papers were selected based on the pre-specified inclusion and exclusion criteria according to the objectives of this systematic review (summarized in Table 1). These criteria were applied by two independent observers (BH, JFC, TRO) in two phases: 1) a pre-screening phase where papers were selected on the basis of title and abstract information; 2) when title and abstract information was not sufficient for the dismissal of a study, the full text was read for a better judgement. Discrepancies in study selection between observers were solved by discussion and, in the case of a continuing disagreement, consultation with a third investigator (LG). Only studies or reviews containing original data were included and no studies were excluded based on language. To minimize bias in the subsequent steps of this systematic review authors were not contacted at this stage, with the only exception of contacting authors to clarify whether specific data belonged to wildtype animals (Pratt et al., 2013). Selected papers were then included for the extraction of study characteristics, outcome data and risk of bias assessment.

**Table 1:** Objectives and inclusion and exclusion criteria for study selection.

Objectives	Inclusion criteria	Exclusion criteria
**Subjects:** all mammals.	*In vivo* studies in which pregnant animals of any species are injected with poly(I:C), with outcomes assessed in the female or male offspring at any pre- or postnatal age.	Human, *ex vivo* and *in vitro* studies; studies in GMOs.
**Type of intervention:** poly(I:C) injection(s) during any stage of pregnancy.	All types of studies which characterize the effect of any dose or frequency of maternal poly(I:C) injection on the offspring; regardless whether drug or treatment intervention is being tested.	Administration of poly(I:C) outside the gestational period.
**Control:** an animal of which the mother has not been exposed to poly(I:C) but has received a sham equivalent.	Studies including an appropriate control group (*e.g.* saline injection).	Studies not including an adequate control group (and controls for double-hit and co-treatment studies, if applicable).
**Outcomes:** immunological outcomes.	Studies reporting cytokine gene or protein expression levels.	Any other (immunological) outcomes (*e.g.* behavioral outcomes, metabolic outcomes); genetic analyses; omics studies (*e.g.* transcriptomics, proteomics).

### 2.2 Extraction of study characteristics

The following study characteristics were extracted by one investigator and a second investigator randomly checked the data entered by the first investigator (BH, JFC, TO).

#### 2.2.1 Animal model characteristics

Species, strain, and sex of the offspring, poly(I:C) dose, vehicle, route of administration, gestational day of poly(I:C) injection, frequency of administration and sham equivalent.

#### 2.2.2 Outcome characteristics

Type of immunological outcome (cytokine name, protein/gene expression), sampling location, offspring’s age at the time of outcome assessment.

### 2.3 Study quality assessment

SYRCLE’s Risk of bias tool was applied using three scoring categories: high/unclear/low risk of bias (Hooijmans et al., 2014). In case insufficient information was reported to judge the risk of bias, it was scored as “unclear”. As part of the application of the tool, we included unit-of-analysis errors and the combination of different measurements or cohorts as a replacement for the category “other”. For the item “selective outcome reporting”, two databases for preregistration of preclinical studies were consulted: Animal Study Registry (German Centre for the Protection of Laboratory Animals, 2019) and Preclinicaltrials (Preclinicaltrials.eu, 2019). Study quality was assessed by two independent researchers and discrepancies were solved by discussion (BH, JFC).

### 2.4 Extraction of outcome data

Descriptive (significantly increased/no effect/significantly decreased cytokine levels compared to control group, and non-detectable levels) and quantitative data (mean, SEM or SD, and sample size) for control and poly(I:C) groups as reported in the paper were collected by one investigator and a second investigator randomly checked the data entered by the first investigator (BH, JFC, TRO). For sample sizes, priority was given to information found in the Results section over the Materials and Methods. When outcome measures were only presented graphically, data were extracted with a digital ruler (Universal Desktop Ruler, AVPSoft.com). Authors were contacted in case of missing or unclear data. If authors were unable to provide the requested information, outcome measures for that study were excluded from the analysis and potentially the systematic review as a whole, depending on the type of information that was missing and if useable data remained.

The extracted study characteristics and outcome data were converted to the required format when this was necessary for analysis. For descriptive summaries and meta-analysis, timing of poly(I:C) administration and offspring’s age were expressed as days. When sample sizes were reported as ranges, the most conservative value was applied to calculate SD.

### 2.5 Data synthesis and meta-analysis

**Fig. 1:**
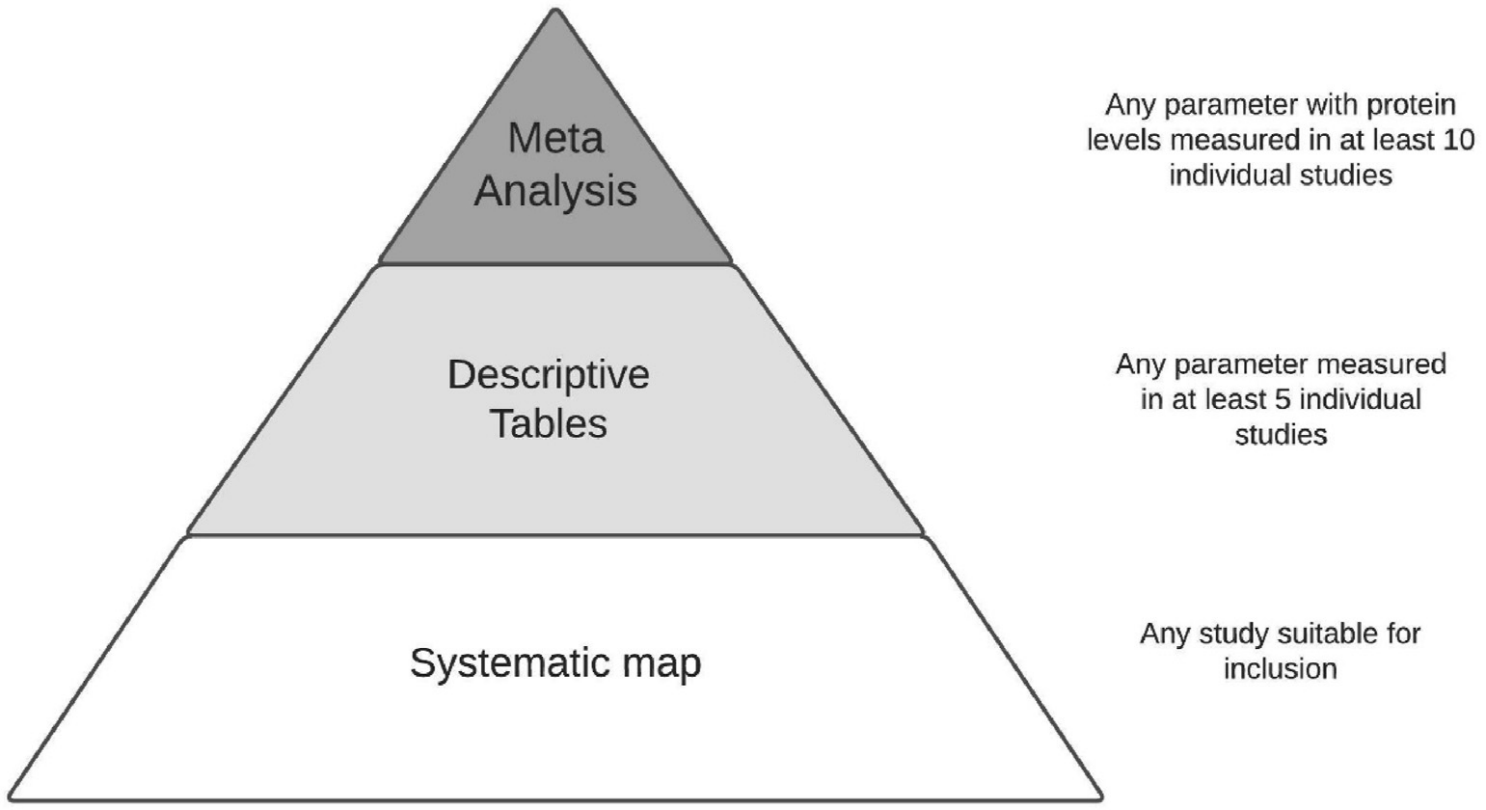
Overview of the distribution of data within this review.

To provide a comprehensive overview of the available evidence, the following selections were applied. The outcome data, quality assessment and study characteristics of any included study can be found in the systematic map (Supplementary file S2). The outcome of any parameter that was measured in at least 5 individual studies is also presented in the descriptive tables, sorted by outcome direction (increased, decreased or no effect). Parameters that had protein concentrations measured in at least 10 individual studies were quantitatively analyzed in the meta-analysis. Fig. 1 provides an overview of where data is presented.

Data included in the meta-analysis was analyzed using Comprehensive Meta-Analysis V3 (Biostat, Inc) in order to pool and visually represent the outcomes. Measurements that were below detection limits were excluded from this assessment. To prevent biological duplicates, only a single sample was included per animal. Gene expression data was excluded from the analysis and when multiple brain sample locations were available, prioritization went as following: Frontal cortex/hippocampus/cingulate cortex/basal ganglia/cerebellum. Two samples of one animal were however included if these were categorized in different subgroups, such as blood and brain samples. While not entirely independent values, these were treated as such. Standardized mean difference (SMD) was used as an effect size measure because included papers had varying units of measurement and species. I^2^ was used as the measure to express statistical heterogeneity.

Planned subgroup analyses were performed for species, age at outcome assessment, gestational period of poly(I:C) injection, sampling location and species. Age of outcome assessment was divided into groups of prenatal, pre-weaning and post-weaning measurements. Gestational period of poly(I:C) administration was divided into groups representing thirds of the pregnancy, defined as early, mid and late gestation. A subgroup was considered eligible for processing if it contained at least five experiments originating from at least three individual papers. Comparisons between subgroups were made using t-tests, with Holm-Bonferroni correction for multiple testing.

The possibility of publication bias was evaluated by plotting the SMD’s of processed parameters against 1/√n, as is recommended for funnel plots using SMD (Zwetsloot et al., 2017). Plots were visually inspected for asymmetry.

## 3 Results

### 3.1 Search results and selection

**Fig. 2:**
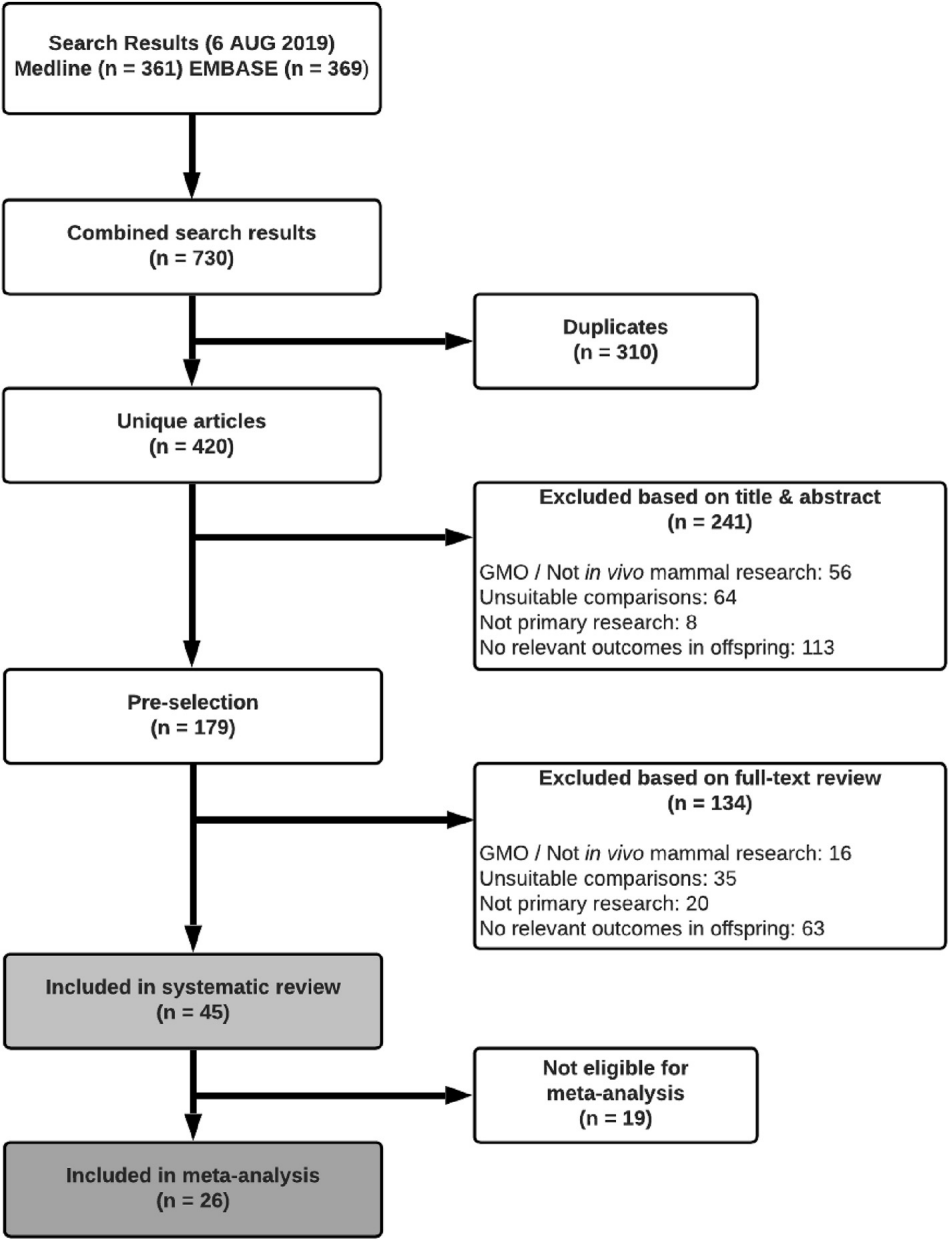
Flow chart for study selection.

Fig. 2 shows the selection procedure for papers. The electronic search retrieved 730 hits, 310 of which were duplicates and 375 of which were not eligible for inclusion. The remaining 45 papers were included, collectively containing 1259 measurements of cytokines and chemokines. The authors of 13 papers were contacted to obtain missing data, from 10 of which a response was received and by 8 of which the requested data was provided.

### 3.2 Description of included studies

Table 2 shows the general characteristics of the 45 included papers, as well as the parameters measured and sampling locations. Because individual studies may perform several measurements with different characteristics, some entries show multiple values.

**Table 2:** Study characteristics.

Article	Species	Strain	Sex	Age	Poly(I:C) dose (mg/kg)	Route	Day of injection (GD)	Sampling locations	*n*	Parameters
Arrode-Brusés et al. (2012)	Mice	C57BL/6J	M&F	Prenatal (G16 ​+ ​3 ​h, G17)	2	IP	16	Brain	9–10	CXCL1, CXCL5, CXCL9, CXCL10, Eotaxin, G-CSF, GM-CSF, IFN-ɣ, IL-1α, IL-1β, IL-2, IL-3, IL-4, IL-5, IL-6, IL-7, IL-9, IL-10, IL-12 (p40), IL-12 (p70), IL-13, IL-15, IL-17, LIF, MCP-1, MCSF, MIP-1α, MIP-1β, MIP-2, RANTES, TNF-α, VEGF
Arsenault et al. (2014)	Mice	C57BL/6	M&F	Prenatal (G18)/Pre-weaning (P10)	5	IV	15–17	Brain, plasma	11-17 (G18), 5-10 (P10)	CD68, GM-CSF, IFN-ɣ, IL-1α, IL-1β, IL-2, IL-3, IL-4, IL-5, IL-6, IL-10, IL-12 (p70), IL-17, MCP-1, MIP-α, RANTES, TNF-α
Clark et al. (2019)	Rats	Wistar	M	Post-weaning (P36, P61)	5	IV	15	Brain	5–11	IFN-ɣ, IL-1β, IL-4, IL-6, IL-10, TNF-α
Connor et al. (2012)	Mice	C57BL/6J	NR	Prenatal (G12.5, G17.5)	5	IV	12.5/17.5	Brain	3–4	IL-6
Corradini et al. (2018)	Mice	C57BL/6	NR	Prenatal(G9 ​+ ​3 ​h, G9 ​+ ​6 ​h, G10)/Post-weaning (P90)	2	IP	9	Brain, whole fetus	4-10 (prenatal), NR (P90)	IL-1β, IL-6, IL-17, TGF-β-1, TNF-α
Ding et al. (2019)	Rats	Sprague Dawley	M&F	Post-weaning (P40, P60)	10	IV	9	Hip, PFC	7–8	IL-1β, IL-6, TNF-α
Duchatel et al. (2018)	Rats	Wistar	M&F	Post-weaning (P70-84)	4	IV	10/19	CiC	12	IL-1β, TNF-α
Ehninger (2014)	Mice	C57BL6/Ncrl (M) ​× ​C57BL6/J (F)	NR	Prenatal (G12.5 ​+ ​2 ​h, G12.5 ​+ ​6.5 ​h)	20	IP	12.5	Brain	≥4	AXL, CD30, CD30L, CD40, CCL1, CCL17, CCL25, CCL27, CXCL1, CXCL4, CXCL5, CXCL9, CXCL10, CXCL12, CXCL13, CXCL16, Eotaxin, Eotaxin-2, Fas ligand, Fractalkine, G-CSF, GM-CSF, IFN-ɣ, IGFBP-3, IGFBP-5, IGFBP-6, IL-1α, IL-1β, IL-2, IL-3, IL-3R-β, IL-4, IL-5, IL-6, IL-9, IL-10, IL-12 (p40), IL-12 (p70), IL-13, IL-17, L-selectin, Leptin, LEP-R, Lymphotactin, MCP-1, MCP-5, MCSF, MIP-1α, MIP-1ɣ, MIP-2, MIP-3α, MIP-3β, P-selectin, RANTES, SCF, TIMP-1, TNF-α, TNF-R1, TNF-R2, TPO, VCAM-1
Garay et al. (2013)	Mice	C57BL/6J	M&F	Pre-weaning (P0, P7, P14)/Post-weaning (P30, P60)	20	IP	12.5	CiC, Hip, FC, serum	5–6	CXCL1, Eotaxin, G-CSF, GM-CSF, IFN-ɣ, IL-1α, IL-1β, IL-2, IL-3, IL-4, IL-5, IL-6, IL-9, IL-10, IL-12 (p40), IL-12 (p70), IL-13, IL-17, MCP-1, MIP-1α, MIP-1β, RANTES, TNF-α
Gilmore et al. (2005)	Rats	Sprague Dawley	NR	Prenatal(G16, G17)/Pre-weaning (P1, P7)	20	IP	16	Brain, liver/spleen, PFC	9	TNF-α
Giovanoli et al. (2013)	Mice	C57BL/6	M&F	Post-weaning (P41, P70)	1	IV	9	Hip, plasma	10–13	IL-1β, IL-6, IL-10, TNF-α
Giovanoli et al. (2015)	Mice	C57BL/6J	M	Post-weaning (P30,150^a^,660^a^)	5	IV	17	Hip, plasma	10–13	IL-1β, IL-4, IL-6, TNF-α
Giovanoli et al. (2016)	Mice	C57BL/6N	M	Post-weaning (P40, P90)	5	IV	9	Hip, plasma	8–10	IL-1β, IL-4, IL-6, TNF-α
Han et al. (2011)	Rats	Sprague Dawley	M	Post-weaning (P44)	0.5	IP	15–18	Serum	6	TNF-α
Han et al. (2017)	Mice	ddY	NR	Post-weaning (P70)	5	IP	12–17	Hip, nucleus accumbens, CA1, CA3, dentate gyrus	5–6	C1q
Hollins et al. (2018)	Rats	Wistar	M	Pre-weaning (P7)/Post-weaning (P84)	5	IV	10,19	Colon	6	IL-1β, IL-6, TNF-α
Horváth et al. (2019)	Mice	C57BL/6	M	Prenatal (G12.5)	3	IP	12.5	Brain	6	CXCL1, IL-1α, IL-1β, IL-6, IL-10, TNF-α
Hu et al. (2019)	Rats	Sprague Dawley	NR	Prenatal (G17)/Pre-weaning (P0)	10	IV	17	PFC	≥3	ISG15
Hui et al. (2018)	Mice	C57BL/6	M&F	Post-weaning (P80-90)	5	IP	9.5	Cer, CeC, Hip	4–5	CD45, IL-1β, IL-6, Fractalkine, Fractalkine receptor, TGF-β-1, TNF-α, TREM-2, Ym1
Krstic et al. (2012)	Mice	C57BL/6J	NR	Pre-weaning (P21)/Post-weaning (P90-150, P450)	5	IV	17	Hip, NeC, plasma	4–7	IL-1α, IL-1β, IL-6, TNF-α
Lipina et al. (2013)	Mice	C57BL/6J	NR	Prenatal (G9)	2.5/5	IV	9	Brain	8	IL-6
MacDowell et al., 2017	Mice	C56BL/6J	M&F	Post-weaning (≥P81)	5	IP	9.5	PFC	9–11	Fractalkine, IFN-α-1, IFN-β, IL-1β, IL-6, IL-10, PPAR-ɣ, TGF-β-1, TNF-α
Mandal et al. (2013)	Mice	C57BL/6	M&F	Post-weaning (P56-70)	10	IP	12	Plasma	5–8	IL-6, IL-10
Mattei et al. (2014)	Rats	Wistar	M	Post-weaning (P128)	4	IV	15	Cer, Hip	5	IL-1β, TNF-α, TNF-R1, TNF-R2
Meyer et al. (2005)	Mice	C57BL6/J	ND	Prenatal (G9)	2.5/5/10	IV^c^	9	Brain	7–10	IFN-ɣ, IL-1β, IL-2, IL-10
Meyer et al. (2006)	Mice	C57BL6/J	NR	Prenatal (G9 ​+ ​3 ​h, G9 ​+ ​6 ​h, G17 ​+ ​3 ​h, G17 ​+ ​6 ​h)	5	IV	9/17	Brain	4–6	IL-1β, IL-6, IL-10, TNF-α
Meyer et al. (2008)	Mice	FVB	NR (G9), M&F (P100)	Prenatal (G9)/Post-weaning (P100)	2	IV	9	Brain, CPu, FC (medial), Hip (dorsal), Hip (ventral)	20 (G9), 10 (P100)	IL-1β, IL-6, IL-10, TNF-α
Missault et al. (2014)	Rats	Wistar-Hannover	NR	Prenatal (G9, G15)	2/4/8	SC	9	Central nervous system	3–8	IL-1β, IL-6, IL-10, TNF-α
Mueller et al., (2019)^b^	Mice	C57BL6/N	NR	Prenatal (G12)	5	IV	12	Brain	6–7	CXCL1, IL-1β, IL-6, IL-10, IL-17, MCP-1, TNF-α
Murray et al. (2019)	Rats	Wistar	M&F	Prenatal (G21)	10	IP	15	Plasma	17–20	IL-6
Nakamura et al. (2019)	Mice	C57BL/6	NR (G17.5) M&F (P91)	Prenatal (G17.5)/Post-weaning (P91-98)	20	IP	17.5	Brain, FC, Hip	4–15	Furin, IL-1β, IL-6, IL-6R-α, TGF-β-1, TGF-β-2, TNF-α
Oh-Nishi et al. (2016)	Rats	Wistar	M	Pre-weaning (P3)/Post-weaning (P63-112)	4	IP	15–18	Serum	6 (P3), 7 (P63-112)	Ig-κ light chain, IL-1β, IL-6, TNF-α
Openshaw et al. (2019)	Mice	C57BL/6	M&F^d^	Prenatal (G12.5)	20	SC	12.5	Brain	4	CXCL1, CXCL9, CXCL10, FGF-2, GM-CSF, IFN-ɣ, IL-1α, IL-1β, IL-2, IL-4, IL-5, IL-6, IL-10, IL-12 (p40), IL-13, IL-17, MCP-1, MIP-1α, RANTES, TNF-α, VEGF-A
Pacheco-Lopez et al. (2013)	Mice	C57BL/6J	M	Post-weaning (P30, P70)	5	IV	9	Plasma	8–9	IFN-ɣ, IL-1β, IL-2, IL-6, IL-10, TNF-α
Pratt et al. (2013)	Mice	C57BL/6J	NR	Prenatal (G16)	20	IP	12.5	Brain (CD11b^+^ cells)	4–6	CXCL5, Eotaxin, GM-CSF, IL-1α, IL-1β, IL-4, IL-6, IL-9, IL-10, MCP-1, MCSF, MIP-1β, RANTES, TNF- α
Ratnayake et al. (2014)	Spiny mice	NA	NR	Prenatal (G20 ​+ ​2 ​h, G21)	5	SC	20	Brain	5	IL-6, TNF-α
Rose et al. (2017)	Rhesus monkeys	NA	M&F	Post-weaning (~P395, ~P1338)	0.25^e^	Injection^c^	43,44,46/100,101,103	Plasma	11–13	G-CSF, GM-CSF, IFN-ɣ, IL-1β, IL-2, IL-4, IL-5, IL-6, IL-8, IL-10, IL-12 (p40), IL-13, IL-17, MCP-1, MIP-1α, MIP-1β, TNF-α
Tsukada et al. (2015)	Mice	C57BL/6J	ND	Prenatal (G12.5 ​+ ​3 ​h, G13.5)	4/20	IP	12.5	CSF	3–4	LIF
Volk et al. (2015)	Mice	C57BL/6J	M&F	Post-weaning (P56)	20	IP	11-13/15-17	PFC	14–16	IFITM1, IFITM2, IFITM3, IFN-β, IL-1β, IL-6, Schnurri-2
Volk et al. (2019)	Mice	C57BL/6J	M&F	Post-weaning (P60)	20	IP	11-13/15-17	PFC	14–16	CD40, IL-1R-1, LTβR, TNF-R1, TNF-R2
Vuillermot et al. (2017)	Mice	C57BL/6N	NR	Prenatal (G9)	5	IV	9	Brain	6	IL-1β, IL-6, TNF-α
Wang et al. (2019)	Mice	C57BL/6J	M	Prenatal (G14.5)	20	IP	12.5	Brain	6	IL-1β, IL-6, IL-17, TNF-α
Wu et al. (2015)	Mice	C57BL/6N	NR	Prenatal (G12.5)	20	IP	12.5	Brain	3–4	IL-6^f^
Yee et al. (2011)	Rats	Sprague Dawley	M	Post-weaning (P69)	4	IV	15	Plasma	6	IL-1β, IL-6, TNF-α
Zhao *et a*l. (2019)	Rats	Wistar	NR (G18) & M (P28)	Prenatal (G18)/Post-weaning (P28)	1/5/10	IP	18	Brain, Cer, FC, Hip	5–6	CCR-2, IFN-ɣ, IL-1β, IL-4, IL-6, IL-10, TNF-α

CA = Cornu Ammonis; CCL = Chemokine (C–C motif) ligand; CCR = C–C chemokine receptor; CD = Cluster of differentiation; CeC = Cerebral cortex; Cer ​= ​Cerebellum; CiC ​= ​Cingulate cortex; CPu = Caudate putamen; CSF = Cerebrospinal fluid; CXCL = Chemokine (C-X-C motif) ligand; F = Female; FC = Frontal cortex; FGF = Fibroblast growth factor; G ​= ​Gestational day; G-CSF; Granulocyte colony-stimulating factor; GM-CSF ​= ​Granulocyte-macrophage colony-stimulating factor; Hip ​= ​Hippocampus; IFN = Interferon; Ig ​= ​Immunoglobulin; IFITM = Interferon-induced transmembrane protein; IGFBP = Insulin-like growth factor-binding protein; IL = Interleukin; IP = Intraperitoneal; ISG = Interferon-stimulated gene; IV = Intravenous; LEP ​= ​Leptin; LIF ​= ​Leukemia inhibitory factor; LT ​= ​Lymphotoxin; M ​= ​Male; MCP ​= ​Monocyte chemoattractant protein; MCSF ​= ​Macrophage colony-stimulating factor; MHCII ​= ​Major histocompatibility complex class II; MIP ​= ​Macrophage inflammatory protein; NA = Not applicable; ND = Not determined; NeC = Neocortex; NR = Not reported; P = Postnatal day; PFC = Prefrontal cortex; Poly(I:C) ​= ​Polyriboinosinic-polyribocytidylic acid; PPAR = Peroxisome proliferator-activated receptor; RANTES ​= ​Regulated on activation normal T cell expressed and secreted; SC = Subcutaneous; SCF = Stem cell factor; TGF ​= ​Transforming growth factor; TIMP ​= ​Tissue inhibitor of metalloproteinases; TNF ​= ​Tumor necrosis factor; TPO ​= ​Thrombopoietin; TREM ​= ​Triggering receptor expressed on monocytes; VCAM = Vascular cell adhesion molecule; VEGF = Vascular endothelial growth factor.

a Measured in two separate cohorts, either undergoing behavioral testing or behaviorally naïve animals.

b Experiments done using six different poly(I:C) batches.

c This study includes a non-injection control group.

d Brain samples within each litter were pooled.

e Poly(I:C) stabilized with poly-L-lysine.

f Measured in two separate cohorts.

All studies but one were performed in rodents. Mice were the most frequently used species (31 studies) and C57BL/6 was the most frequently used mouse strain (28 studies). Among the C57BL/6 studies however, a variety of substrains was used. Thirteen studies were performed in rats. These include seven studies in Wistar rats, five studies in Sprague Dawley rats and one study in Wistar-Hannover rats. The remaining two studies were performed in rhesus monkeys and spiny mice. The sex of the offspring studied was mostly either mixed (28 studies) or male (11 studies). Seven papers did not report the sex of at least a part of the animals used in the study.

The methods used to induce maternal immune activation using poly(I:C) injection varied between studies as well. Doses ranged from as low as 0,25 ​mg/kg to as high as 20 ​mg/kg and were administered either a single (38 studies) or multiple (8 studies) times. The gestational day at which poly(I:C) was injected varied, but in most studies poly(I:C) administration occurred in either the mid or late gestational period, with only one exception injecting during early gestation (Rose et al., 2017). The administration route was generally intravenous (21 studies) or intraperitoneal (22 studies). Three studies administered poly(I:C) subcutaneously. The ages at which samples were collected from the offspring also varied considerably. The most frequently used sampling periods were prenatal and post-weaning, with 24 and 21 studies collecting at least part of the samples from these periods respectively. Pre-weaning samples were taken in only five studies. The included studies measured a variety of immunological parameters in various locations. The brain was the most researched organ, with 38 studies sampling at least part of the brain. The specific brain region of interest, however, varied considerably. Thirteen studies included blood samples and whole fetus, colon and liver/spleen samples were each reported in a single study.

**Fig. 3:**
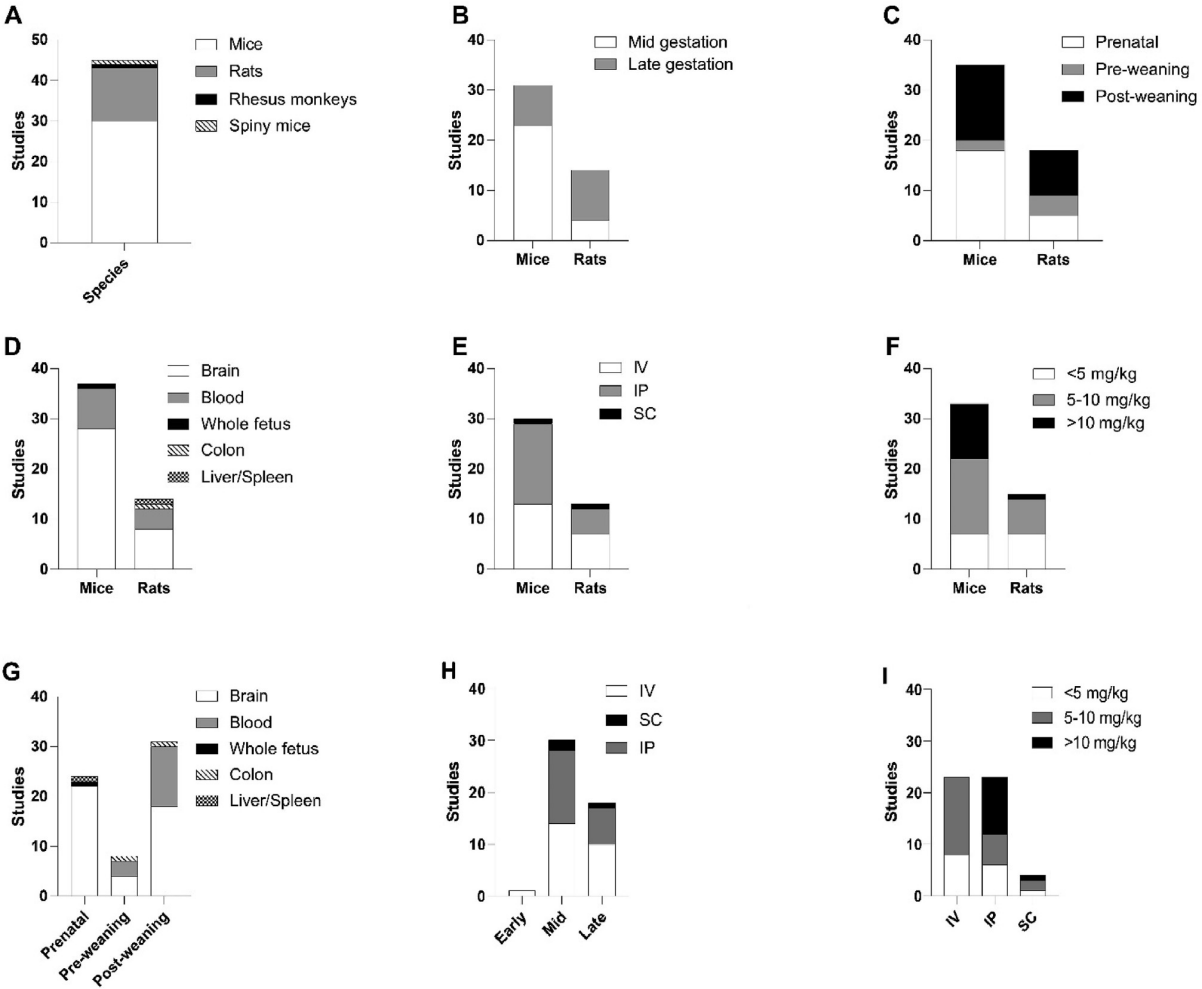
Qualitative analysis of included studies. Distribution of species (A), gestational period of poly(I:C) administration (B), age of outcome assessment (C), sampling location by species (D), administration route (E), poly(I:C) dose by species (F), sampling location by age of outcome assessment (G), administration route by gestational period of poly(I:C) injection (H) and poly(I:C) dose by administration route (I).

Fig. 3 schematically summarizes the most important study characteristics from Table 2, showing how many studies utilized certain species, injection techniques, injection timepoints, doses and ages of outcome assessment. Studies that measured or injected at multiple timepoints are represented multiple times in the figure. For clarity, spiny mouse and rhesus monkey data have been excluded from the figures showing data by species, but are available in the systematic map (Supplementary file S2).

### 3.3 Risk of bias assessment and quality of reporting

**Fig. 4:**
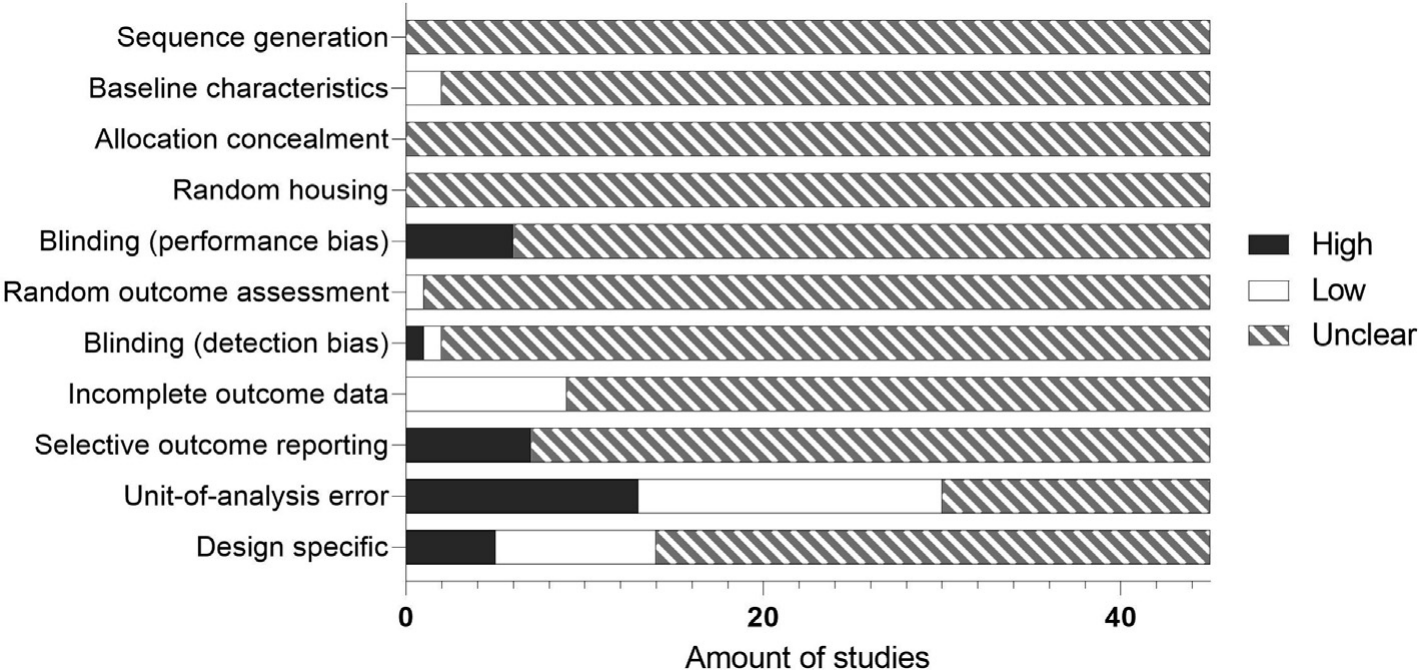
Results of risk of bias assessment.

The designs described in the 45 included papers were checked for risk of bias using the SYRCLE Risk of bias tool for animal studies (Hooijmans et al., 2014). Fig. 4 shows the outcomes of these assessments per type of bias. Scores for individual studies can be found in the systematic map (Supplementary file S2). If an article took adequate measures to avoid or minimize a certain type of bias, the risk of bias was scored as low. Alternatively, if it could be concluded that no adequate measures were taken to avoid a risk of bias, it was high. If insufficient information was given to judge whether adequate measures were taken, the risk of bias was scored as unclear.

As shown in Fig. 4, the way in which the included studies dealt with risks of bias was mostly unclear. Most explicitly high risks of bias were found in the “Unit-of-analysis error” category, with 13 studies defining the experimental unit as the individual pup or including an insufficient number of dams per group, thus risking bias through the litter effect. “Selective outcome reporting” was scored as high when the methods mention measurements for which the outcomes were not shown in the results. “Blinding”, either for performance or detection bias, was scored as high when it could be determined that no adequate blinding was used.

### 3.4 Descriptive tables

The descriptive tables present the outcome directions of all immune parameters that were reported in at least 5 individual studies. In the three descriptive tables the outcome measurements are sorted by varying study characteristics.

Table 3 provides an overview of immune parameters sorted by sampling location. Each individual measurement is reported in the table. Measurements performed in whole blood, plasma or serum are combined as “Blood”. Additionally, measurements reported to be performed in either frontal or prefrontal cortex are combined as “Frontal cortex”.

**Table 3:**
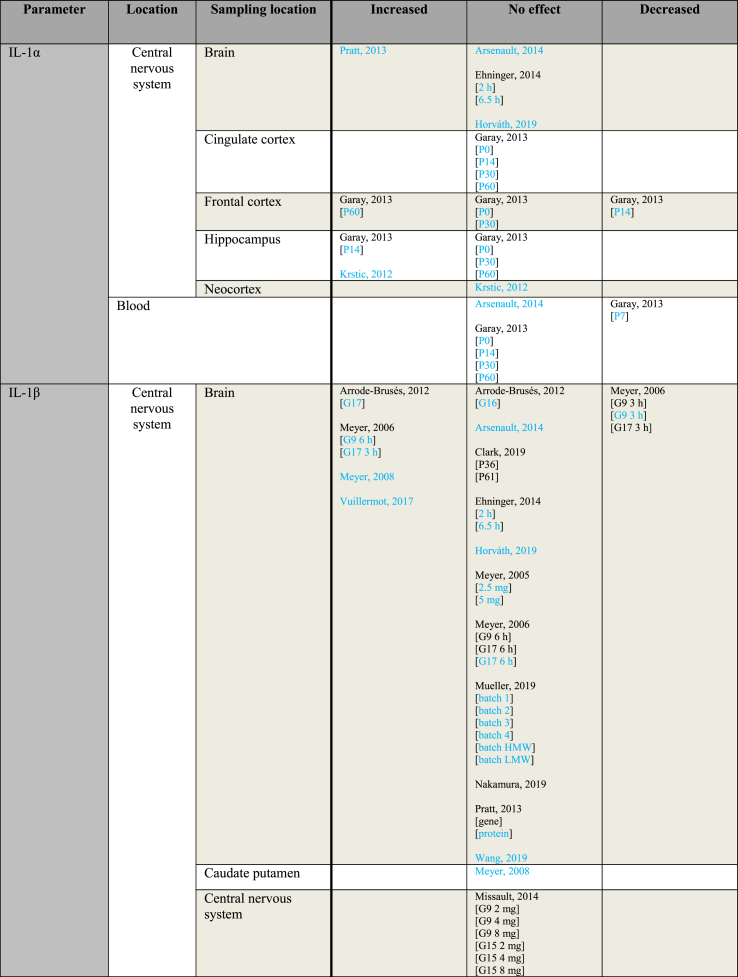

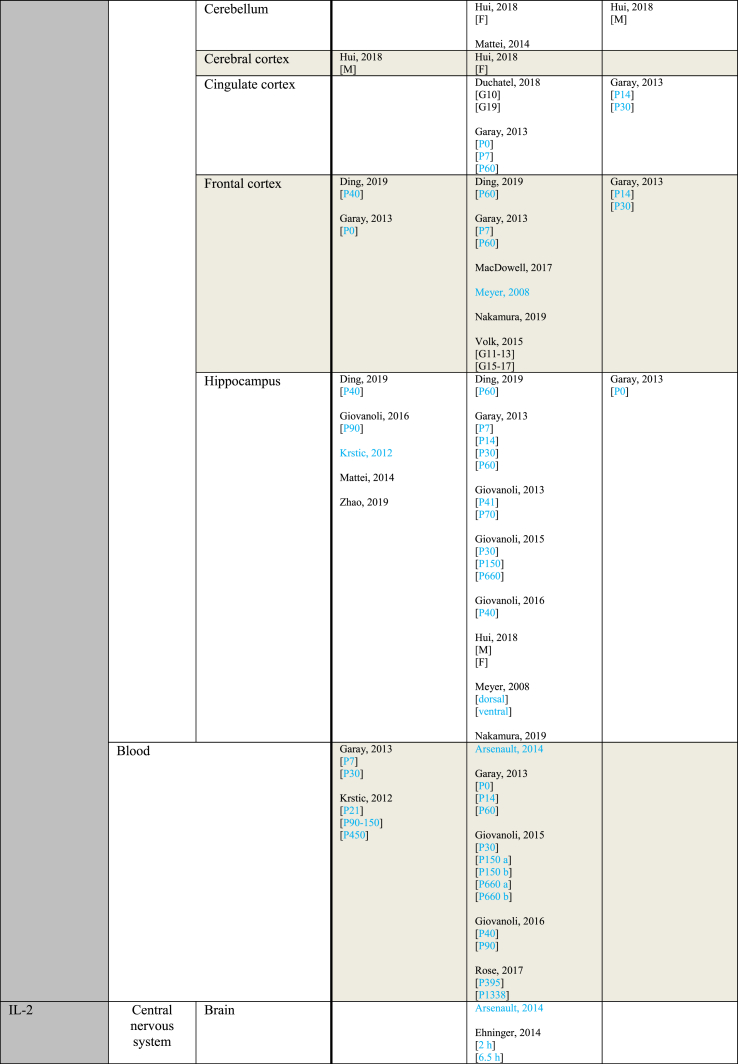

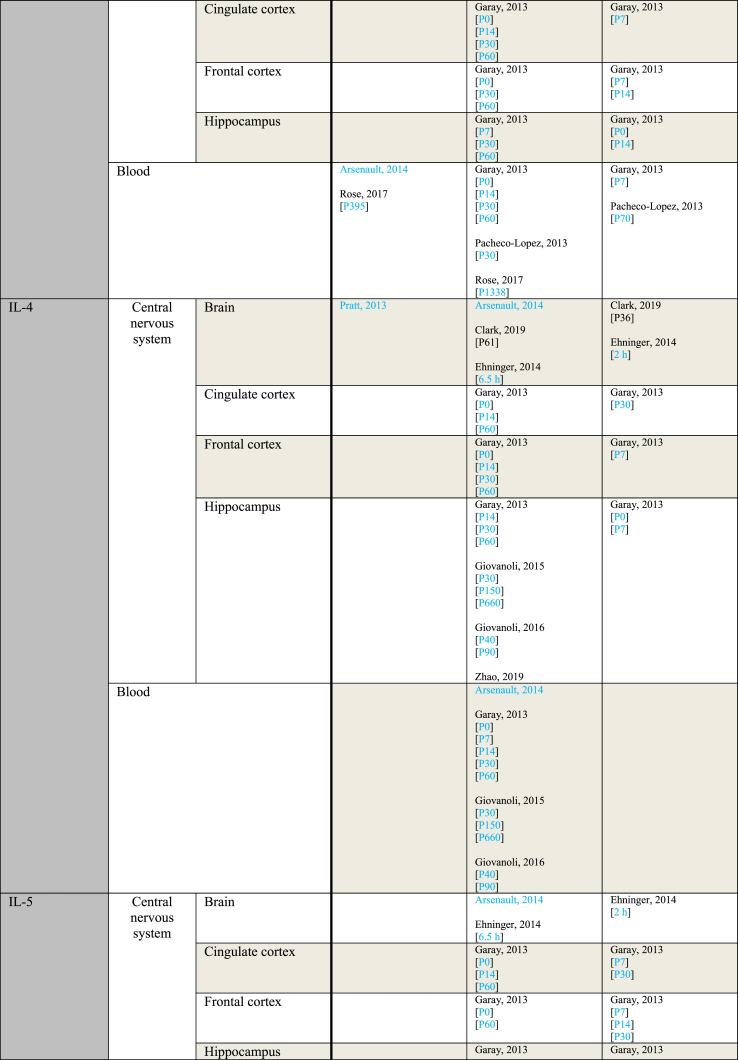

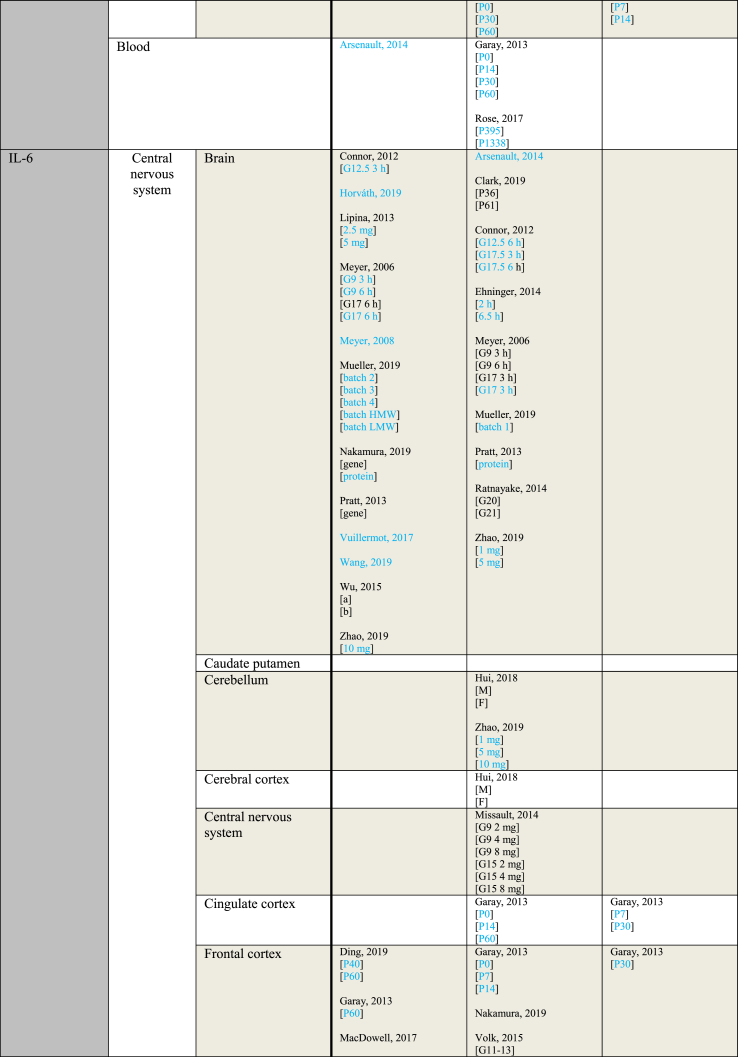

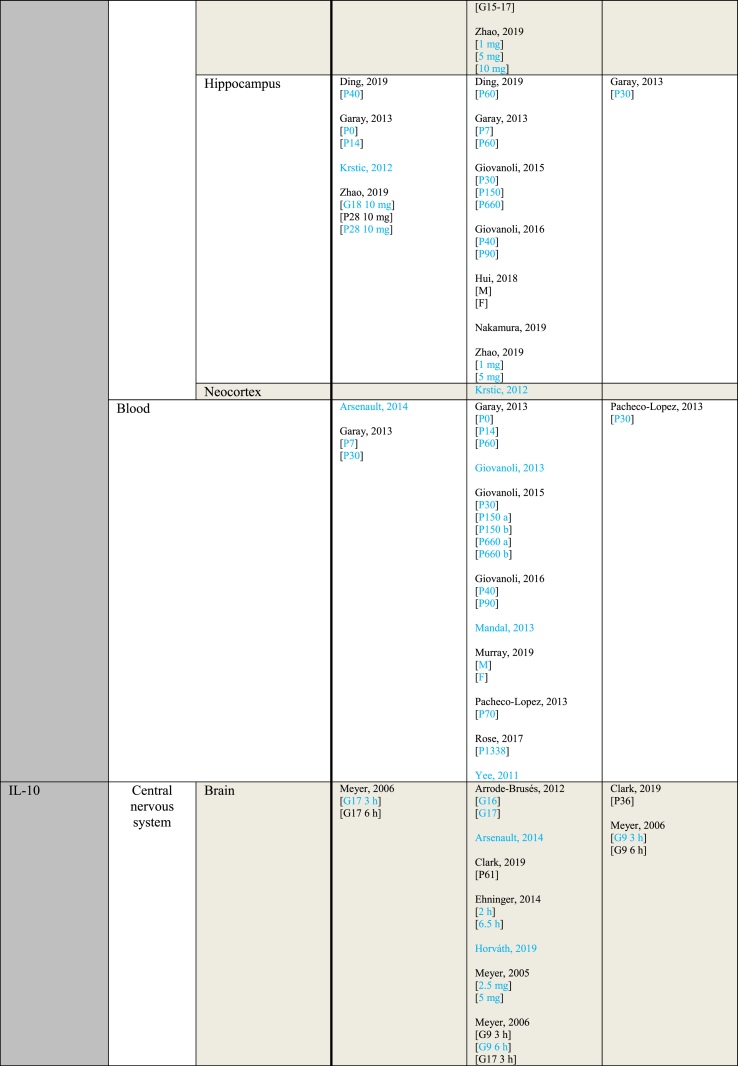

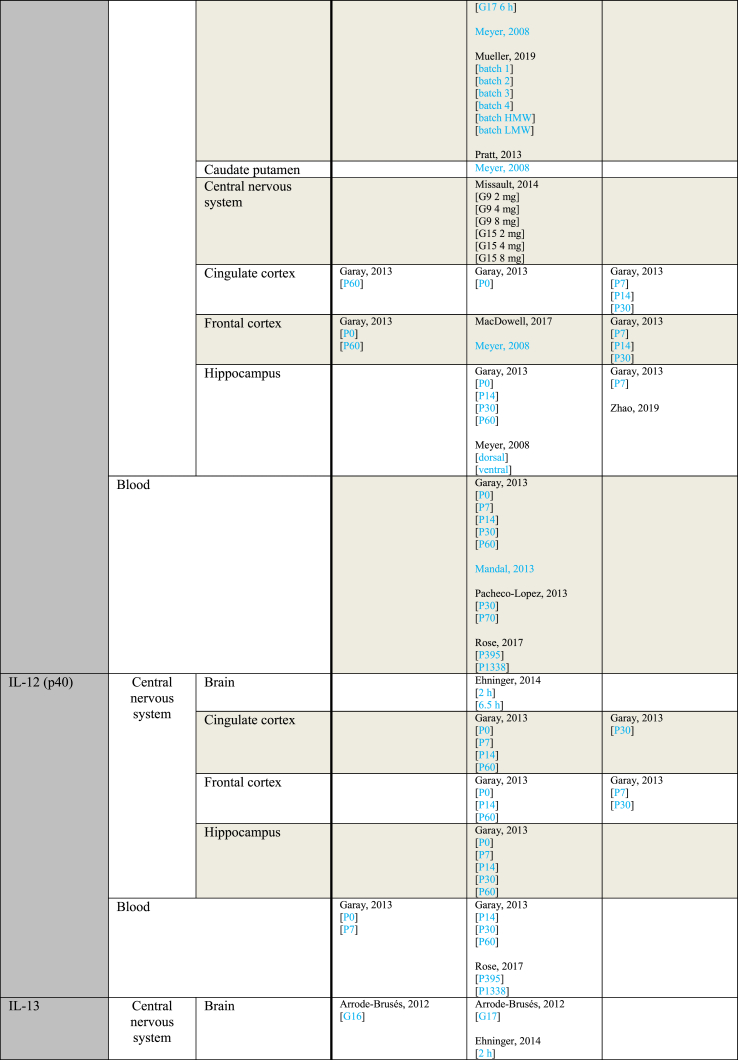

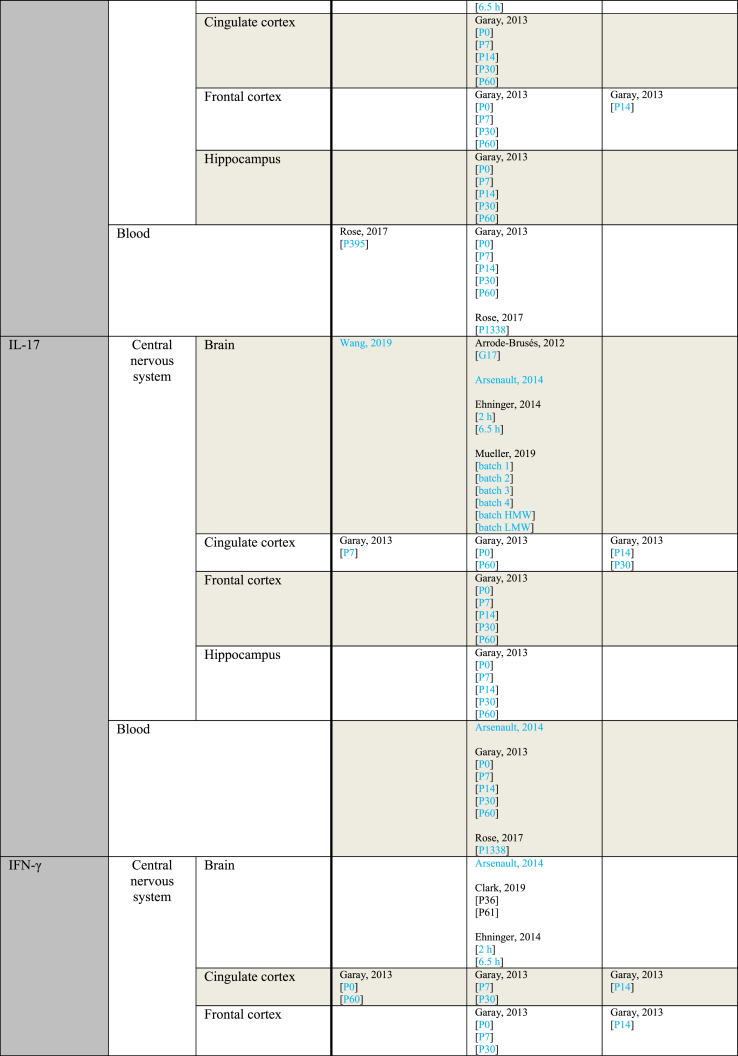

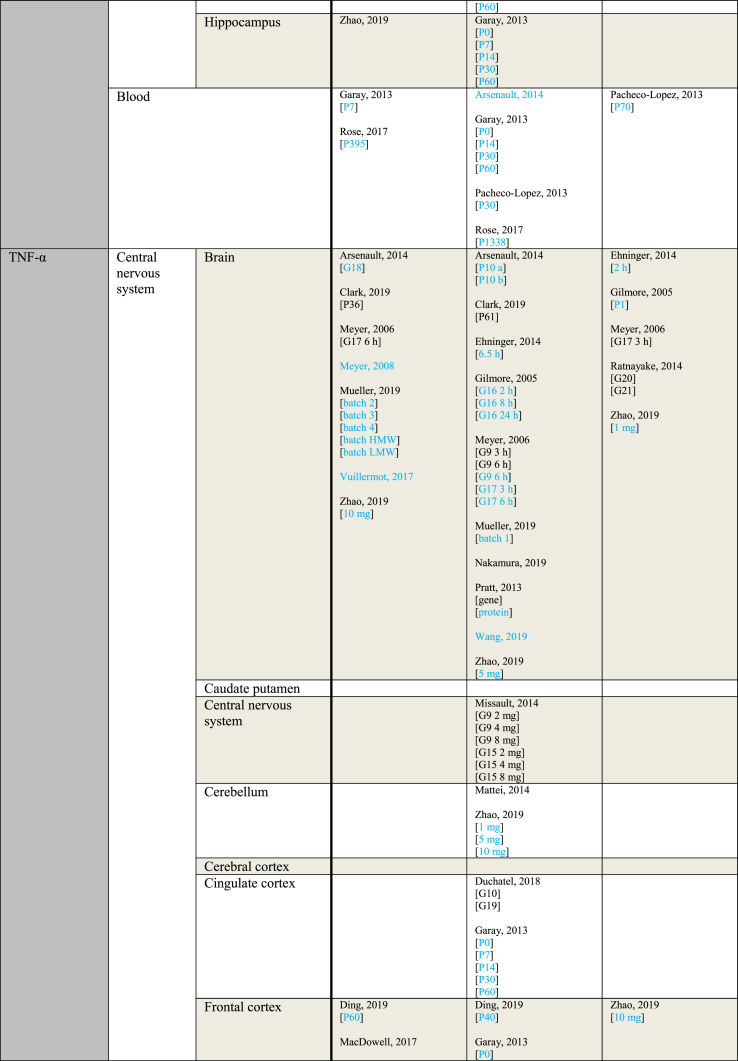

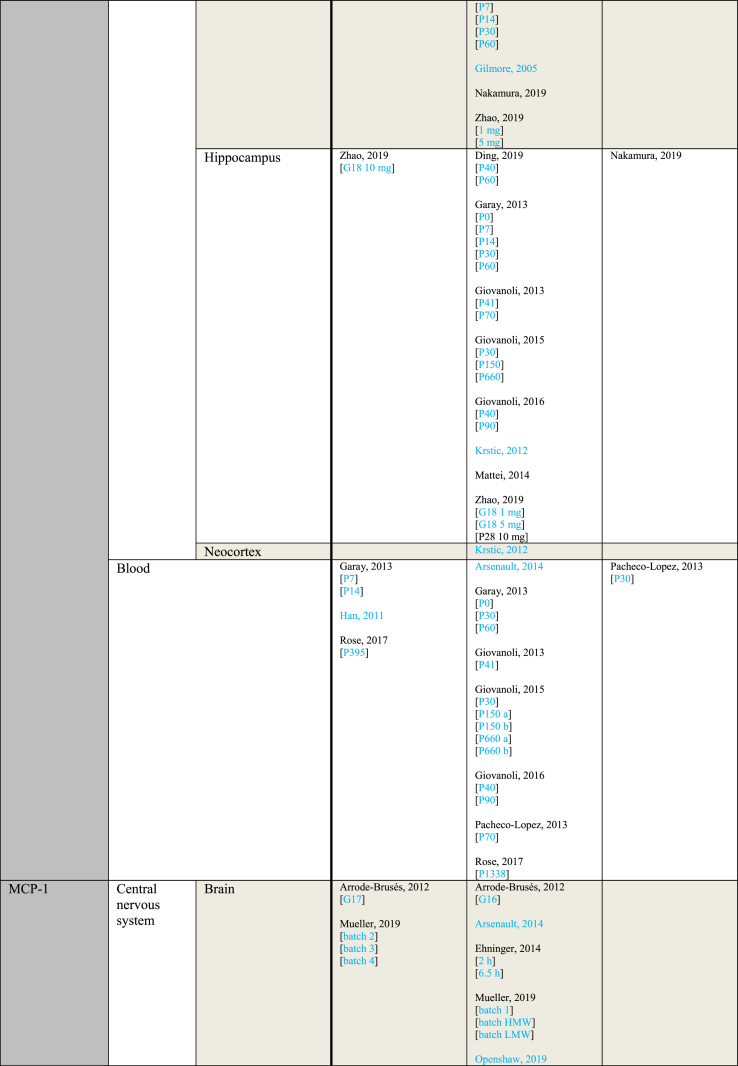

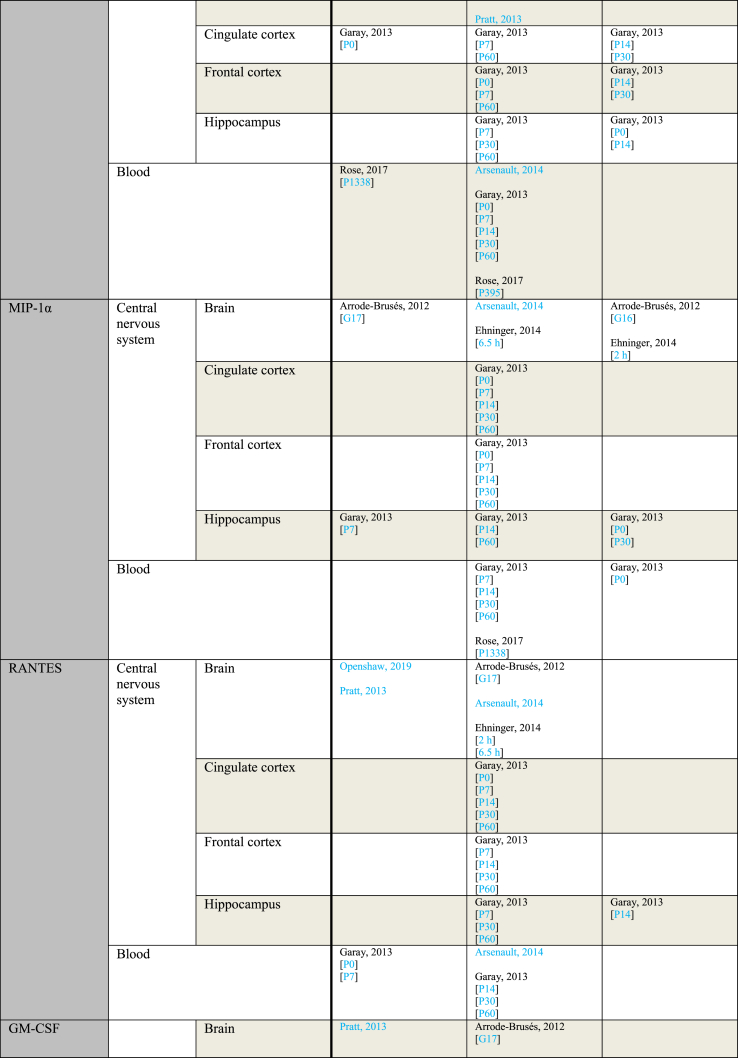

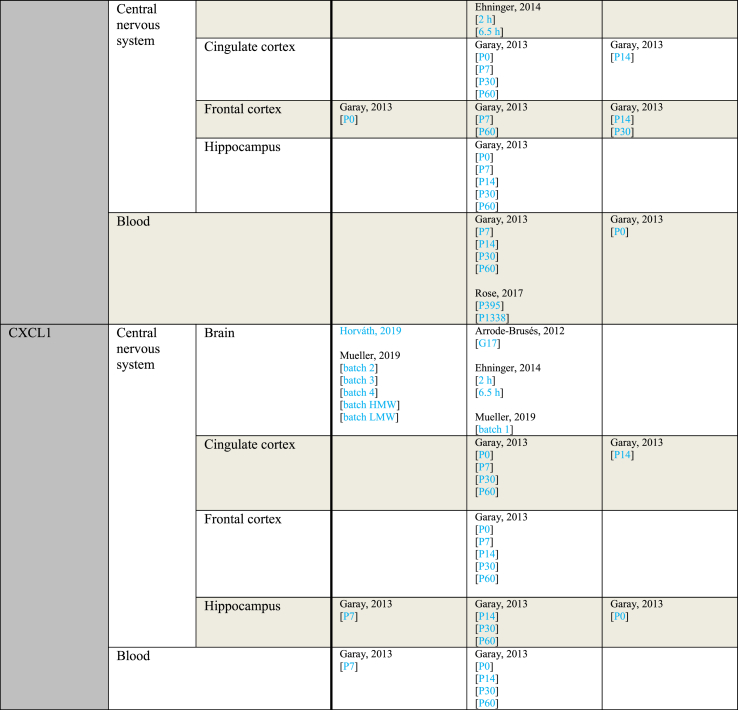
Immune parameters Outcome measurements sorted by sampling location.

Entries indicated in blue represent protein expression data, all other entries represent gene expression data.

CXCL = Chemokine (C-X-C motif) ligand; F = Female; G ​= ​Gestational day; GM-CSF ​= ​Granulocyte-macrophage colony-stimulating factor; HMW ​= ​high molecular weight; IFN = Interferon; IL = Interleukin; LMW ​= ​low molecular weight; M ​= ​Male; MCP ​= ​Monocyte chemoattractant protein; MIP ​= ​Macrophage inflammatory protein; P = Postnatal day; RANTES ​= ​Regulated on activation normal T cell expressed and secreted; TNF ​= ​Tumor necrosis factor.

As shown in Table 3, most papers reported no significant effect of maternal immune activation on cytokines in the offspring. Regarding outcomes that were significantly changed, there was an overall equal amount of significant increases and decreases. The most frequently studied locations of interest were whole brain, blood, frontal cortex and hippocampus.

Table 4 provides an overview of outcome measurements sorted by the gestational period during which poly(I:C) was administered to the mother. When a study reported using multiple injections, the gestational period during which the first injection was administered is considered the gestational period from which the outcome originates. An exception is made for the only study that contains data from both early and mid gestation, which is grouped under “mid gestation” for clarity (Rose et al., 2017).

**Table 4:** Outcome measurements sorted by the gestational period of poly(I:C) administration.

Parameter	Gestational period (GP)	Increased	No effect	Decreased
IL-1α	Mid gestation	Garay, 2013 [P14 brain] [P60 FC] Pratt, 2013	Ehninger, 2014 [2 ​h] [6.5 ​h] Garay, 2013 [P0 blood] [P0 CiC] [P0 FC] [P0 Hip] [P14 blood] [P14 CiC] [P30 blood] [P30 CiC] [P30 FC] [P30 Hip] [P60 blood] [P60 CiC] [P60 Hip] Horváth, 2019	Garay, 2013 [P7 blood] [P14 FC]
	Late gestation	Krstic, 2012 [Hip]	Arsenault, 2014 [blood] [brain] Krstic, 2012 [NeC]	
IL-1β	Mid gestation	Corradini, 2018 [6 ​h Mg2+ run] [6 ​h] Ding, 2019 [P40 FC] [P40 Hip] Garay, 2013 [P0 FC] [P7 blood] [P30 blood] Giovanoli, 2016 [P90 Hip] Hui, 2018 [M CeC] Meyer, 2006 [6 ​h] Meyer, 2008 [brain] Vuillermot, 2017	Corradini, 2018 [3 ​h] Ding, 2019 [P60 FC] [P60 Hip] Duchatel, 2018 Ehninger, 2014 [2 ​h] [6.5 ​h] Garay, 2013 [P0 blood] [P0 CiC] [P14 blood] [P14 Hip] [P30 Hip] [P60 blood] [P60 CiC] [P60 FC] [P60 Hip] Giovanoli, 2013 [P41 Hip] [P70 Hip] Giovanoli, 2016 [P40 blood] [P40 Hip] [P90 blood] Horváth, 2019 Hui, 2018 [F CeC] [F Cer] [F Hip] [M Hip] MacDowell, 2017 Meyer, 2005 [2.5 ​mg] [5 ​mg] Meyer, 2006 [6 ​h] Meyer, 2008 [CPu] [FC] [Hip dorsal] [Hip ventral] Missault, 2014 [2 ​mg] [4 ​mg] [8 ​mg] Mueller, 2019 [batch 1] [batch 2] [batch 3] [batch 4] [batch HMW] [batch LMW] Pratt, 2013 [gene] [protein] Rose, 2017a [P395] [P1338] Volk, 2015 Wang, 2019	Corradini, 2018 [24 ​h] Garay, 2013 [P0 Hip] [P14 CiC] [P14 FC] [P30 CiC] [P30 FC] Hui, 2018 [M Cer] Meyer, 2006 [3 ​h] [3 ​h]
	Late gestation	Arrode-Brusés, 2012 [G17] Krstic, 2012 [P21 blood] [P90-150 blood] [P450 blood] [P450 Hip] Mattei, 2014 [Hip] Meyer, 2006 [3 ​h] Zhao, 2019	Arrode-Brusés, 2012 [G16] Arsenault, 2014 [blood] [brain] Clark, 2019 [P36] [P61] Duchatel, 2018 Giovanoli, 2015 [P30 blood] [P30 Hip] [P150 a blood] [P150 a Hip] [P150 b blood] [P660 a blood] [P660 a Hip] [P660 b blood] Mattei, 2014 [Cer] Meyer, 2006 [6 ​h] [6 ​h] Missault, 2014 [2 ​mg] [4 ​mg] [8 ​mg] Nakamura, 2019 [brain] [FC] [Hip] Volk, 2015	Meyer, 2006 [3 ​h]
IL-2	Mid gestation	Rose, 2017a [P395]	Ehninger, 2014 [2 ​h] [6.5 ​h] Garay, 2013 [P0 blood] [P0 CiC] [P0 FC] [P7 Hip] [P14 blood] [P14 CiC] [P30 blood] [P30 CiC] [P30 FC] [P30 Hip] [P60 blood] [P60 CiC] [P60 FC] [P60 Hip] Pacheco-Lopez, 2013 [P30] Rose, 2017a [P1338]	Garay, 2013 [P0 Hip] [P7 blood] [P7 CiC] [P7 FC] [P14 FC] [P14 Hip] Pacheco-Lopez, 2013 [P70]
	Late gestation	Arsenault, 2014 [blood]	Arsenault, 2014 [brain]	
IL-4	Mid gestation	Pratt, 2013	Ehninger, 2014 [6 ​h] Garay, 2013 [P0 blood] [P0 CiC] [P0 FC] [P7 blood] [P14 blood [P14 CiC] [P14 FC] [P14 Hip] [P30 blood] [P30 FC] [P30 Hip] [P60 blood] [P60 CiC] [P60 FC] [P60 Hip] Giovanoli, 2016 [P40 blood] [P40 Hip] [P90 blood] [P90 Hip]	Ehninger, 2014 [2 ​h] Garay, 2013 [P0 Hip] [P7 FC] [P7 Hip] [P30 CiC]
	Late gestation		Arsenault, 2014 [blood] [brain] Clark, 2019 [P61] Giovanoli, 2015 [P30 blood] [P30 Hip] [P150 blood] [P150 Hip] [P660 blood] [P660 Hip] Zhao, 2019	Clark, 2019 [P36]
IL-5	Second trimester		Ehninger, 2014 [6.5 ​h] Garay, 2013 [P0 blood] [P0 CiC] [P0 FC] [P0 Hip] [P14 blood] [P14 CiC] [P30 blood] [P30 Hip] [P60 blood] [P60 CiC] [P60 FC] [P60 Hip] Rose, 2017a [P395] [1338]	Ehninger, 2014 [2 ​h] Garay, 2013 [P7 CiC] [P7 FC] [P7 Hip] [P14 FC] [P14 Hip] [P30 CiC] [P30 FC]
	Late gestation	Arsenault, 2014 [blood]	Arsenault, 2014 [brain]	
IL-6	Mid gestation	Connor, 2012 [3 ​h] Corradini, 2018 [6 ​h Mg2+ run] [6 ​h] Ding, 2019 [P40 FC] [P40 Hip] [P60 FC] Garay, 2013 [P0 Hip] [P7 blood] [P14 Hip] [P30 blood] [P60 FC] Hollins, 2018 [P7] Horváth, 2019 Lipina, 2012 [2.5 ​mg] [5 ​mg] MacDowell, 2017 Meyer, 2006 [3 ​h] [6 ​h] Meyer, 2008 [brain] Mueller, 2019 [batch 2] [batch 3] [batch 4] [batch HMW] [batch LMW] Pratt, 2013 [gene] Vuillermot, 2017 Wang, 2019 Wu, 2015 [a] [b]	Connor, 2012 [6 ​h] Corradini, 2018 [3 ​h] [24 ​h] Ding, 2019 [P60 Hip] Ehninger, 2014 [2 ​h] [6.5 ​h] Garay, 2013 [P0 blood] [P0 CiC] [P0 FC] [P7 FC] [P7 Hip] [P14 blood] [P14 CiC] [P14 FC] [P60 blood] [P60 CiC] [P60 Hip] Giovanoli, 2013 [P41 blood] Giovanoli, 2016 [P40 blood] [P40 Hip] [P90 blood] [P90 Hip] Hui, 2018 [F CeC] [F Cer] [F Hip] [M CeC] [M Cer] [M Hip] Mandal, 2013 Meyer, 2006 [3 ​h] [6 ​h] Missault, 2014 [2 ​mg] [4 ​mg] [8 ​mg] Mueller, 2019 [batch 1] Pacheco-Lopez, 2013 [P70] Pratt, 2013 [protein] Ratnayake, 2014 [G20] [G21] Rose, 2017a [P1338] Volk, 2015	Garay, 2013 [P7 CiC] [P30 CiC] [P30 FC] [P30 Hip] Pacheco-Lopez, 2013 [P30]
	Late gestation	Arsenault, 2014 [blood] Krstic, 2012 [Hip] Meyer, 2006 [6 ​h] [6 ​h] Nakamura, 2019 [brain] [brain] Zhao, 2019 [G18 10 ​mg brain] [G18 10 ​mg Hip] [P28 10 ​mg Hip] [P28 10 ​mg Hip]	Arsenault, 2014 [brain] Clark, 2019 [P36] [P61] Connor, 2012 [3 ​h] [6 ​h] Giovanoli, 2015 [P30 blood] [P30 Hip] [P150 a blood] [P150 a Hip] [P150 b blood] [P660 a blood] [P660 a Hip] [P660 b blood] Krstic, 2012 [NeC] Meyer, 2006 [3 ​h] [3 ​h] Missault, 2014 [2 ​mg] [4 ​mg] [8 ​mg] Murray, 2019 [F] [M] Nakamura, 2019 [FC] [Hip] Volk, 2015 Yee, 2011 Zhao, 2019 [G18 1 ​mg brain] [G18 1 ​mg Cer] [G18 1 ​mg FC] [G18 1 ​mg Hip] [G18 5 ​mg brain] [G18 5 ​mg Cer] [G18 5 ​mg FC] [G18 5 ​mg Hip] [G18 10 ​mg Cer] [G18 10 ​mg FC]	
IL-10	Mid gestation	Garay, 2013 [P0 FC] [P60 CiC] [P60 FC]	Ehninger, 2014 [2 ​h] [6.5 ​h] Garay, 2013 [P0 blood] [P0 CiC] [P0 Hip] [P7 blood] [P14 blood [P14 Hip] [P30 blood] [P30 Hip] [P60 blood] [P60 Hip] Horváth, 2019 MacDowell, 2017 Mandal, 2013 Meyer, 2005 [2.5 ​mg] [5 ​mg] Meyer, 2006 [3 ​h] [6 ​h] Meyer, 2008 [brain] [CPu] [FC] [Hip dorsal] [Hip ventral] Missault, 2014 [2 ​mg] [4 ​mg] [8 ​mg] Mueller, 2019 [batch 1] [batch 2] [batch 3] [batch 4] [batch HMW] [batch LMW] Pacheco-Lopez, 2013 [P30] [P70] Pratt, 2013 Rose, 2017a [P395] [P1338]	Garay, 2013 [P7 CiC] [P7 FC] [P7 Hip] [P14 CiC] [P14 FC] [P30 CiC] [P30 FC] Meyer, 2006 [3 ​h] [6 ​h]
	Late gestation	Meyer, 2006 [3 ​h] [6 ​h]	Arrode-Brusés, 2012 [G16] [G17] Arsenault, 2014 [brain] Clark, 2019 [P61] Meyer, 2006 [3 ​h] [6 ​h] Missault, 2014 [2 ​mg] [4 ​mg] [8 ​mg]	Clark, 2019 [P36] Zhao, 2019
IL-12 (p40)	Mid gestation	Garay, 2013 [P0 blood] [P7 blood]	Ehninger, 2014 [2 ​h] [6.5 ​h] Garay, 2013 [P0 CiC] [P0 FC] [P0 Hip] [P7 Cic] [P7 Hip] [P14 blood] [P14 CiC] [P14 FC] [P14 Hip] [P30 blood] [P30 Hip] [P60 blood] [P60 CiC] [P60 FC] [P60 Hip] Rose, 2017a [P395] [P1338]	Garay, 2013 [P7 FC] [P30 CiC] [P30 FC]
IL-13	Mid gestation	Rose, 2017a [P395]	Ehninger, 2014 [2 ​h] [6.5 ​h] Garay, 2013 [P0 blood] [P0 CiC] [P0 FC] [P0 Hip] [P7 blood] [P7 CiC] [P7 FC] [P7 Hip] [P14 blood] [P14 CiC] [P14 Hip] [P30 blood] [P30 CiC] [P30 FC] [P30 Hip] [P60 blood] [P60 CiC] [P60 FC] [P60 Hip] Rose, 2017a [P1338]	Garay, 2013 [P14 FC]
	Late gestation	Arrode-Brusés, 2012 [G16]	Arrode-Brusés, 2012 [G17]	
IL-17	Mid gestation	Garay, 2013 [P7 CiC] Wang, 2019	Corradini, 2018 Ehninger, 2014 [2 ​h] [6.5 ​h] Garay, 2013 [P0 blood] [P0 CiC] [P0 FC] [P0 Hip] [P7 blood] [P7 FC] [P7 Hip] [P14 blood] [P14 FC] [P14 Hip] [P30 blood] [P30 FC] [P30 Hip] [P60 blood] [P60 CiC] [P60 FC] [P60 Hip] Mueller, 2019 [batch 1] [batch 2] [batch 3] [batch 4] [batch HMW] [batch LMW] Rose, 2017a [P1338]	Garay, 2013 [P14 CiC] [P30 CiC]
	Late gestation		Arrode-Brusés, 2012 [G17] Arsenault, 2014 [blood] [brain]	
IFN-γ	Mid gestation	Garay, 2013 [P0 CiC] [P7 blood] [P60 CiC] Rose, 2017a [P395]	Ehninger, 2014 [2 ​h] [6.5 ​h] Garay, 2013 [P0 blood] [P0 FC] [P0 Hip] [P7 CiC] [P7 FC] [P7 Hip] [P14 blood] [P14 Hip] [P30 blood] [P30 CiC] [P30 FC] [P30 Hip] [P60 blood] [P60 FC] [P60 Hip] Pacheco-Lopez, 2013 [P30] Rose, 2017a [P1338]	Garay, 2013 [P14 CiC] [P14 FC] Pacheco-Lopez, 2013 [P70]
	Late gestation	Zhao, 2019	Arsenault, 2014 [blood] [brain] Clark, 2019 [P36] [P61]	
TNF-α	Mid gestation	Ding, 2019 [P60 FC] Garay, 2013 [P7 blood] [P14 blood] MacDowell, 2017 Meyer, 2008 [brain] Mueller, 2019 [batch 2] [batch 3] [batch 4] [batch HMW] [batch LMW] Rose, 2017a [P395] Vuillermot, 2019	Ding, 2019 [P40 FC] [P40 Hip] [P60 Hip] Duchatel, 2018 Ehninger, 2014 [6.5 ​h] Garay, 2013 [P0 blood] [P0 CiC] [P0 FC] [P0 Hip] [P7 CiC] [P7 FC] [P7 Hip] [P14 CiC] [P14 FC] [P14 Hip] [P30 blood] [P30 CiC] [P30 FC] [P30 Hip] [P60 blood] [P60 CiC] [P60 FC] [P60 Hip] Giovanoli, 2013 [P41 blood] [P41 Hip] [P70 Hip] Giovanoli, 2016 [P40 blood] [P40 Hip] [P90 blood] [P90 Hip] Hollins, 2018 [P7] [P84] Meyer, 2006 [3 ​h] [6 ​h] [6 ​h] Missault, 2014 [2 ​mg] [4 ​mg] [8 ​mg] Mueller, 2019 [batch 1] Pacheco-Lopez, 2013 [P70] Pratt, 2013 [gene] [protein] Rose, 2017a [P1338] Wang, 2019	Ehninger, 2014 [2 ​h] Pacheco-Lopez, 2013 [P30] Ratnayake, 2014 [G20] [G21]
	Late gestation	Arsenault, 2014 [G18] Clark, 2019 [P36] Han, 2011 Meyer, 2006 [6 ​h] Zhao, 2019 [G18 10 ​mg brain] [G18 10 ​mg Hip]	Arsenault, 2014 [P10 a] [P10 b] [P10 blood] Clark, 2019 [P61] Duchatel, 2018 Gilmore, 2005 [2 ​h brain] [8 ​h brain] [8 ​h liver & spleen] [24 ​h brain] [24 ​h liver & spleen] [P7] Giovanoli, 2015 [P30 blood] [P30 Hip] [P150 a blood] [P150 a Hip] [P150 b blood] [P660 a blood] [P660 a Hip] [P660 b blood] Krstic, 2012 [Hip] [NeC] Mattei, 2014 [Cer] [Hip] Meyer, 2006 [3 ​h] [6 ​h] Missault, 2014 [2 ​mg] [4 ​mg] [8 ​mg] Nakamura, 2019 [brain] [FC] Zhao, 2019 [G18 1 ​mg Cer] [G18 1 ​mg FC] [G18 1 ​mg Hip] [G18 5 ​mg brain] [G18 5 ​mg Cer] [G18 5 ​mg FC] [G18 5 ​mg Hip] [G18 10 ​mg Cer] [P28 10 ​mg Hip]	Gilmore, 2005 [P1] [2 ​h liver & spleen] Meyer, 2006 [3 ​h] Nakamura, 2019 [Hip] Zhao, 2019 [G18 1 ​mg brain] [G18 10 ​mg FC]
MCP-1	Mid gestation	Garay, 2013 [P0 CiC] Mueller, 2019 [batch 2] [batch 3] [batch 4] Rose, 2017a [P1338]	Ehninger, 2014 [2 ​h] [6.5 ​h] Garay, 2013 [P0 blood] [P0 FC] [P7 blood] [P7 CiC] [P7 FC] [P7 Hip] [P14 blood] [P30 blood] [P30 Hip] [P60 blood] [P60 CiC] [P60 FC] [P60 Hip] Mueller, 2019 [batch 1] [batch HMW] [batch LMW] Openshaw, 2019 Pratt, 2013 Rose, 2017a [P395]	Garay, 2013 [P0 Hip] [P14 CiC] [P14 FC] [P14 Hip] [P30 CiC] [P30 FC]
	Late gestation	Arrode-Brusés, 2012 [G17]	Arrode-Brusés, 2012 [G16] Arsenault, 2014 [blood] [brain]	
MIP-1α	Mid gestation	Garay, 2013 [P7 Hip]	Ehninger, 2014 [6.5 ​h] Garay, 2013 [P0 CiC] [P0 FC] [P7 blood] [P7 CiC] [P7 FC] [P14 blood] [P14 CiC] [P14 FC] [P14 Hip] [P30 blood] [P30 CiC] [P30 FC] [P60 blood] [P60 CiC] [P60 FC] [P60 Hip] Rose, 2017a [P1338]	Ehninger, 2014 [2 ​h] Garay, 2013 [P0 blood] [P0 Hip] [P30 Hip]
	Late gestation	Arrode-Brusés, 2012 [G17]	Arsenault, 2014 [blood] [brain]	Arrode-Brusés, 2012 [G16]
RANTES	Mid gestation	Garay, 2013 [P0 blood] [P7 blood] Openshaw, 2019 Pratt, 2013	Ehninger, 2014 [2 ​h] [6.5 ​h] Garay, 2013 [P0 CiC] [P7 CiC] [P7 FC] [P7 Hip] [P14 blood] [P14 CiC] [P14 FC] [P30 blood] [P30 CiC] [P30 FC] [P30 Hip] [P60 blood] [P60 CiC] [P60 FC] [P60 Hip]	Garay, 2013 [P14 Hip]
	Late gestation		Arrode-Brusés, 2012 [G17] Arsenault, 2014 [blood] [brain]	
GM-CSF	Mid gestation	Garay, 2013 [P0 FC] Pratt, 2013	Ehninger, 2014 [2 ​h] [6.5 ​h] Garay, 2013 [P0 CiC] [P0 Hip] [P7 blood] [P7 CiC] [P7 FC] [P7 Hip] [P14 blood] [P14 Hip] [P30 blood] [P30 CiC] [P30 Hip] [P60 blood] [P60 CiC] [P60 FC] [P60 Hip] Rose, 2017a [P395] [P1338]	Garay, 2013 [P0 blood] [P14 CiC] [P14 FC] [P30 FC]
	Late gestation		Arrode-Brusés, 2012 [G17]	
CXCL1	Mid gestation	Garay, 2013 [P7 blood] [P7 Hip] Horváth, 2019 Mueller, 2019 [batch 2] [batch 3] [batch 4] [batch HMW] [batch LMW]	Ehninger, 2014 [2 ​h] [6.5 ​h] Garay, 2013 [P0 blood] [P0 CiC] [P0 FC] [P7 CiC] [P7 FC] [P14 blood] [P14 FC] [P14 Hip] [P30 blood] [P30 CiC] [P30 FC] [P30 Hip] [P60 blood] [P60 CiC] [P60 FC] [P60 Hip] Mueller, 2019 [batch 1]	Garay, 2013 [P0 Hip] [P14 CiC]
	Late gestation		Arrode-Brusés, 2012 [G17]	

Entries indicated in blue represent protein expression data, all other entries represent gene expression data.

a Outcomes derived from a combination of animals exposed to poly(I:C) in either early or mid gestation.

CeC = Cerebral cortex; Cer = Cerebellum; CiC = Cingulate cortex; CPu = Caudate putamen; CXCL = Chemokine (C-X-C motif) ligand; F= Female; FC = Frontal cortex; G = Gestational day; GM-CSF = Granulocyte-macrophage colony-stimulating factor; Hip = Hippocampus; HMW = high molecular weight; IFN = Interferon; IL = Interleukin; LMW = low molecular weight; M = Male; MCP = Monocyte chemoattractant protein; MIP = Macrophage inflammatory protein; NeC = Neocortex; P = Postnatal day; RANTES = Regulated on activation normal T cell expressed and secreted; TNF = Tumor necrosis factor.

Notably more data originated from poly(I:C) induced maternal immune activation in the mid than the late gestational period, providing 977 and 315 measurements respectively over all different parameters.

Table 5 provides an overview of outcomes sorted by subject age at the time of sampling. Every sample taken at a gestational day rather than a postnatal day was considered prenatal. For studies using rats and mice, P0–P20 was considered pre-weaning. Postnatal day 21 and onwards were considered post-weaning. For rhesus monkeys, the length of weaning seems to vary, but generally does not exceed 10 months (Reitsema et al., 2015). Since the earliest sampling time of the study using rhesus monkeys was 1 year, samples from this study were considered post-weaning (Rose et al., 2017).

**Table 5:** Outcome measurements sorted by age of outcome assessment.

Parameter	Age of outcome assessment	Increased	No effect	Decreased
IL-1α	Prenatal	Pratt, 2013	Ehninger, 2014 [2 ​h] [6.5 ​h] Horváth, 2019	
	Pre-weaning	Garay, 2013 [P14 Hip]	Arsenault, 2014 [blood] [brain] Garay, 2013 [P0 blood] [P0 CiC] [P0 FC] [P0 Hip] [P14 blood] [P14 CiC]	Garay, 2013 [P7 blood] [P14 FC]
	Post-weaning	Garay, 2013 [P60 FC] Krstic, 2012 [Hip]	Garay, 2013 [P30 blood] [P30 CiC] [P30 FC] [P30 Hip] [P60 blood] [P60 CiC] [P60 Hip] Krstic, 2012 [NeC]	
IL-1β	Prenatal	Arrode-Brusés, 2012 [G17] Corradini, 2018 [6 ​h Mg2+ run] [6 ​h] Meyer, 2006 [G9 6 ​h] [G17 3 ​h] Meyer, 2008 [brain] Vuillermot, 2017	Arrode-Brusés, 2012 [G16] Corradini, 2018 [3 ​h] Ehninger, 2014 [2 ​h] [6.5 ​h] Horváth, 2019 Meyer, 2005 [2.5 ​mg] [5 ​mg] Meyer, 2006 [G9 6 ​h] [G17 6 ​h] [G17 6 ​h] Missault, 2014 [G9 2 ​mg] [G9 4 ​mg] [G9 8 ​mg] [G15 2 ​mg] [G15 4 ​mg] [G15 8 ​mg] Mueller, 2019 [batch 1] [batch 2] [batch 3] [batch 4] [batch HMW] [batch LMW] Nakamura, 2019 [brain] Pratt, 2013 [gene] [protein] Wang, 2019	Corradini, 2018 [24 ​h] Meyer, 2006 [G9 3 ​h] [G9 3 ​h] [G17 3 ​h]
	Pre-weaning	Garay, 2013 [P0 FC] [P7 blood] Krstic, 2012	Arsenault, 2014 [blood] [brain] Garay, 2013 [P0 blood] [P0 CiC] [P7 CiC] [P7 FC] [P7 Hip] [P14 blood] [P14 Hip] Hollins, 2018	Garay, 2013 [P0 Hip] [P14 CiC] [P14 FC]
	Post-weaning	Ding, 2019 [P40 FC] [P40 Hip] Garay, 2013 [P30 blood] Giovanoli, 2016 [P90 Hip] Hui, 2018 [M CeC] Krstic, 2012 [P90-150 blood] [P450 blood] [P450 Hip] Mattei, 2014 [Hip] Zhao, 2019	Clark, 2019 [P36] [P61] Ding, 2019 [P60 FC] [P60 Hip] Duchatel, 2018 [G10] [G19] Garay, 2013 [P30 Hip] [P60 blood] [P60 CiC] [P60 FC] [P60 Hip] Giovanoli, 2013 [P41 Hip] [P70 Hip] Giovanoli, 2015 [P30 blood] [P30 Hip] [P150 a blood] [P150 a Hip] [P150 b blood] [P660 a blood] [P660 a Hip] [P660 b blood] Giovanoli, 2016 [P40 blood] [P40 Hip] [P90 blood] Hui, 2018 [F CeC] [F Cer] [F Hip] [M Hip] MacDowell, 2017 Mattei, 2014 [Cer] Meyer, 2008 [CPu] [FC] [Hip dorsal] [Hip ventral] Nakamura, 2019 [FC] [Hip] Rose, 2017 [P395] [P1338] Volk, 2015 [G11-13] [G15-17]	Garay, 2013 [P30 CiC] [P30 FC] Hui, 2018 [M Cer]
IL-2	Prenatal		Ehninger, 2014 [2 ​h] [6.5 ​h]	
	Pre-weaning	Arsenault, 2014 [blood]	Arsenault, 2014 [brain] Garay, 2013 [P0 blood] [P0 CiC] [P0 FC] [P7 Hip] [P14 blood] [P14 CiC]	Garay, 2013 [P0 Hip] [P7 blood] [P7 CiC] [P7 FC] [P14 FC] [P14 Hip]
	Post-weaning	Rose, 2017 [P395]	Garay, 2013 [P30 blood] [P30 CiC] [P30 FC] [P30 Hip] [P60 blood] [P60 CiC] [P60 FC] [P60 Hip] Pacheco-Lopez, 2013 [P30] Rose, 2017 [P1338]	Pacheco-Lopez, 2013 [P70]
IL-4	Prenatal	Pratt, 2013	Ehninger, 2014 [6.5 ​h]	Ehninger, 2014 [2 ​h]
	Pre-weaning		Arsenault, 2014 [blood] [brain] Garay, 2013 [P0 blood] [P0 CiC] [P0 FC] [P7 blood] [P14 blood] [P14 CiC] [P14 FC] [P14 Hip]	Garay, 2013 [P0 Hip] [P7 FC] [P7 Hip]
	Post-weaning		Clark, 2019 [P61] Garay, 2013 [P30 blood] [P30 FC] [P30 Hip] [P60 blood] [P60 CiC] [P60 FC] [P60 Hip] Giovanoli, 2015 [P30 blood] [P30 Hip] [P150 blood] [P150 Hip] [P660 blood] [P660 Hip] Giovanoli, 2016 [P40 blood] [P40 Hip] [P90 blood] [P90 Hip] Zhao, 2019	Clark, 2019 [P36] Garay, 2013 [P30 CiC]
IL-5	Prenatal		Ehninger, 2014 [6.5 ​h]	Ehninger, 2014 [2 ​h]
	Pre-weaning	Arsenault, 2014 [blood]	Arsenault, 2014 [brain] Garay, 2013 [P0 blood] [P0 CiC] [P0 FC] [P0 Hip] [P14 blood] [P14 CiC]	Garay, 2013 [P7 CiC] [P7 FC] [P7 Hip] [P14 FC] [P14 Hip]
	Post-weaning		Garay, 2013 [P30 blood] [P30 Hip] [P60 blood] [P60 CiC] [P60 FC] [P60 Hip] Rose, 2017 [P395] [P1338]	Garay, 2013 [P30 CiC] [P30 FC]
IL-6	Prenatal	Connor, 2012 [G12.5 3 ​h] Corradini, 2018 [6 ​h Mg2+ run] [6 ​h] Horváth, 2019 Lipina, 2013 [2.5 ​mg] [5 ​mg] Meyer, 2006 [G9 3 ​h] [G9 6 ​h] [G17 6 ​h] [G17 6 ​h] Meyer, 2008 Mueller, 2019 [batch 2] [batch 3] [batch 4] [batch HMW] [batch LMW] Nakamura, 2019 [gene] [protein] Pratt, 2013 [gene] Vuillermot, 2017 Wang, 2019 Wu, 2015 [a] [b] Zhao, 2019 [10 ​mg brain] [10 ​mg Hip]	Connor, 2012 [G12.5 6 ​h] [G17.5 3 ​h] [G17.5 6 ​h] Corradini, 2018 [3 ​h] [24 ​h] Ehninger, 2014 [2 ​h] [6.5 ​h] Meyer, 2006 [G9 3 ​h] [G9 6 ​h] [G17 3 ​h] [G17 3 ​h] Missault, 2014 [G9 2 ​mg] [G9 4 ​mg] [G9 8 ​mg] [G15 2 ​mg] [G15 4 ​mg] [G15 8 ​mg] Mueller, 2019 [batch 1] Pratt, 2013 [protein] Ratnayake, 2014 [G20] [G21] Zhao, 2019 [1 ​mg brain] [1 ​mg Cer] [1 ​mg FC] [1 ​mg Hip] [5 ​mg brain] [5 ​mg Cer] [5 ​mg FC] [5 ​mg Hip] [10 ​mg Cer] [10 ​mg FC]	
	Pre-weaning	Arsenault, 2014 [blood] Garay, 2013 [P0 Hip] [P7 blood] [P14 Hip]	Arsenault, 2014 [brain] Garay, 2013 [P0 blood] [P0 CiC] [P0 FC] [P7 FC] [P7 Hip] [P14 blood] [P14 CiC] [P14 FC] Murray, 2019 [F] [M]	Garay, 2013 [P7 CiC]
	Post-weaning	Ding, 2019 [P40 FC] [P40 Hip] [P60 FC] Garay, 2013 [P30 blood] [P60 FC] Krstic, 2012 [Hip] MacDowell, 2017 Zhao, 2019 [gene] [protein]	Clark, 2019 [P36] [P61] Ding, 2019 [P60 Hip] Garay, 2013 [P60 blood] [P60 CiC] [P60 Hip] Giovanoli, 2013 [P41 blood] Giovanoli, 2015 [P30 blood] [P30 Hip] [P150 a blood] [P150 a Hip] [P150 b blood] [P660 a blood] [P660 a Hip] [P660 b blood] Giovanoli, 2016 [P40 blood] [P40 Hip] [P90 blood] [P90 Hip] Hollins, 2018 [P84] Hui, 2018 [F CeC] [F Cer] [F Hip] [M CeC] [M Cer] [M Hip] Krstic, 2012 [NeC] Mandal, 2013 Murray, 2019 Nakamura, 2019 [FC] [Hip] Pacheco-Lopez, 2013 [P70] Rose, 2017 [P1338] Volk, 2015 [G11-13] [G15-17] Yee, 2011	Garay, 2013 [P30 CiC] [P30 FC] [P30 Hip] Pacheco-Lopez, 2013 [P30]
IL-10	Prenatal	Meyer, 2006 [G17 3 ​h] [G17 6 ​h]	Arrode-Brusés, 2012 [G16] [G17] Ehninger, 2014 [2 ​h] [6.5 ​h] Horváth, 2019 Meyer, 2005 [2.5 ​mg] [5 ​mg] Meyer, 2006 [G9 3 ​h] [G9 6 ​h] [G17 3 ​h] [G17 6 ​h] Meyer, 2008 Missault, 2014 [G9 2 ​mg] [G9 4 ​mg] [G9 8 ​mg] [G15 2 ​mg] [G15 4 ​mg] [G15 8 ​mg] Mueller, 2019 [batch 1] [batch 2] [batch 3] [batch 4] [batch HMW] [batch LMW] Pratt, 2013	Meyer, 2006 [G9 3 ​h] [G9 6 ​h]
	Pre-weaning	Garay, 2013 [P0 FC]	Arsenault, 2014 [brain] Garay, 2013 [P0 blood] [P0 CiC] [P0 Hip] [P7 blood] [P14 blood] [P14 Hip]	Garay, 2013 [P7 CiC] [P7 FC] [P7 Hip] [P14 CiC] [P14 FC]
	Post-weaning	Garay, 2013 [P60 CiC] [P60 FC]	Clark, 2019 [P61] Garay, 2013 [P30 blood] [P30 Hip] [P60 blood] [P60 Hip] MacDowell, 2017 Mandal, 2013 Meyer, 2008 [CPu] [FC] [Hip dorsal] [Hip ventral] Pacheco-Lopez, 2013 [P30] [P70] Rose, 2017 [P395] [P1338]	Clark, 2019 [P36] Garay, 2013 [P30 CiC] [P30 FC] Zhao, 2019
IL-12 (p40)	Prenatal		Ehninger, 2014 [2 ​h] [6.5 ​h]	
	Pre-weaning	Garay, 2013 [P0 blood] [P7 blood]	Garay, 2013 [P0 CiC] [P0 FC] [P0 Hip] [P7 CiC] [P7 Hip] [P14 blood] [P14 CiC] [P14 FC] [P14 Hip]	Garay, 2013 [P7 FC]
	Post-weaning		Garay, 2013 [P30 blood] [P30 Hip] [P60 blood] [P60 CiC] [P60 FC] [P60 Hip] Rose, 2017 [P395] [P1338]	Garay, 2013 [P30 CiC] [P30 FC]
IL-13	Prenatal	Arrode-Brusés, 2012 [G16]	Arrode-Brusés, 2012 [G17] Ehninger, 2014 [2 ​h] [6.5 ​h]	
	Pre-weaning		Garay, 2013 [P0 blood] [P0 CiC] [P0 FC] [P0 Hip] [P7 blood] [P7 CiC] [P7 FC] [P7 Hip] [P14 blood] [P14 CiC] [P14 Hip]	Garay, 2013 [P14 FC]
	Post-weaning	Rose, 2017 [P395]	Garay, 2013 [P30 blood] [P30 CiC] [P30 FC] [P30 Hip] [P60 blood] [P60 CiC] [P60 FC] [P60 Hip] Rose 2017 [P1338]	
IL-17	Prenatal	Wang, 2019	Arrode-Brusés, 2012 [G17] Corradini, 2018 Ehninger, 2014 [2 ​h] [6.5 ​h] Mueller, 2019 [batch 1] [batch 2] [batch 3] [batch 4] [batch HMW] [batch LMW]	
	Pre-weaning	Garay, 2013 [P7 CiC]	Arsenault, 2014 [blood] [brain] Garay, 2013 [P0 blood] [P0 CiC] [P0 FC] [P0 Hip] [P7 blood] [P7 FC] [P7 Hip] [P14 blood] [P14 FC] [P14 Hip]	Garay, 2013 [P14 CiC]
	Post-weaning		Garay, 2013 [P30 blood] [P30 FC] [P30 Hip] [P60 blood] [P60 CiC] [P60 FC] [P60 Hip] Rose, 2017 [P1338]	Garay, 2013 [P30 CiC]
IFN-γ	Prenatal		Ehninger, 2014 [2 ​h] [6.5 ​h]	
	Pre-weaning	Garay, 2013 [P0 CiC] [P7 blood]	Arsenault, 2014 [blood] [brain] Garay, 2013 [P0 blood] [P0 FC] [P0 Hip] [P7 CiC] [P7 FC] [P7 Hip] [P14 blood] [P14 Hip]	Garay, 2013 [P14 CiC] [P14 FC]
	Post-weaning	Garay, 2013 [P60 CiC] Rose, 2017 [P395] Zhao, 2019	Clark, 2019 [P36] [P61] Garay, 2013 [P30 blood] [P30 CiC] [P30 FC] [P30 Hip] [P60 blood] [P60 FC] [P60 Hip] Pacheco-Lopez, 2013 [P30] Rose, 2017 [P1338]	Pacheco-Lopez, 2013 [P70]
TNF-α	Prenatal	Arsenault, 2014 Meyer, 2006 [G17 6 ​h] Meyer, 2008 Mueller, 2019 [batch 2] [batch 3] [batch 4] [batch HMW] [batch LMW] Vuillermot, 2017 Zhao, 2019 [10 ​mg brain] [10 ​mg Hip]	Ehninger, 2014 [6.5 ​h] Gilmore, 2005 [2 ​h brain] [8 ​h brain] [8 ​h liver & spleen] [24 ​h brain] [24 ​h liver & spleen] Meyer, 2006 [G9 3 ​h] [G9 6 ​h] [G9 6 ​h] [G17 3 ​h] [G17 6 ​h] Missault, 2014 [G9 2 ​mg] [G9 4 ​mg] [G9 8 ​mg] [G15 2 ​mg] [G15 4 ​mg] [G15 8 ​mg] Mueller, 2019 [batch 1] Nakamura, 2019 Pratt, 2013 [gene] [protein] Wang, 2019 Zhao, 2019 [1 ​mg Cer] [1 ​mg FC] [1 ​mg Hip] [5 ​mg brain] [5 ​mg Cer] [5 ​mg FC] [5 ​mg Hip] [10 ​mg Cer]	Ehninger, 2014 [2 ​h] Gilmore, 2005 [2 ​h liver & spleen] Meyer, 2006 [G17 3 ​h] Ratnayake, 2014 [G20] [G21] Zhao, 2019 [1 ​mg brain] [10 ​mg FC]
	Pre-weaning	Garay, 2013 [P7 blood] [P14 blood]	Arsenault, 2014 [P10 a] [P10 b] [P10 blood] Garay, 2013 [P0 blood] [P0 CiC] [P0 FC] [P0 Hip] [P7 CiC] [P7 FC] [P7 Hip] [P14 CiC] [P14 FC] [P14 Hip] Gilmore, 2005 [P7] Hollins, 2018 [P7]	Gilmore, 2005 [P1]
	Post-weaning	Clark, 2019 [P36] Ding, 2019 [P60 FC] Han, 2011 MacDowell, 2017 Rose, 2017 [P395]	Clark, 2019 [P61] Ding, 2019 [P40 FC] [P40 Hip] [P60 Hip] Duchatel, 2018 [G10] [G19] Garay, 2013 [P30 blood] [P30 CiC] [P30 FC] [P30 Hip] [P60 blood] [P60 CiC] [P60 FC] [P60 Hip] Giovanoli, 2013 [P41 blood] [P41 Hip] [P70 Hip] Giovanoli, 2015 [P30 blood] [P30 Hip] [P150 a blood] [P150 a Hip] [P150 b blood] [P660 a blood] [P660 a Hip] [P660 b blood] Giovanoli, 2016 [P40 blood] [P40 Hip] [P90 blood] [P90 Hip] Hollins, 2018 [P84] Krstic, 2012 [Hip] [NeC] Mattei, 2014 [Cer] [Hip] Nakamura, 2019 [FC] Pacheco-Lopez, 2013 [P70] Rose, 2017 [P1338] Zhao, 2019	Nakamura, 2019 [Hip] Pacheco-Lopez, 2013 [P30]
MCP-1	Prenatal	Arrode-Brusés, 2012 [G17] Mueller, 2019 [batch 2] [batch 3] [batch 4]	Arrode-Brusés, 2012 [G16] Ehninger, 2014 [2 ​h] [6.5 ​h] Mueller, 2019 [batch 1] [batch HMW] [batch LMW] Openshaw, 2019 Pratt, 2013	
	Pre-weaning	Garay, 2013 [P0 CiC]	Arsenault, 2014 [blood] [brain] Garay, 2013 [P0 blood] [P0 FC] [P7 blood] [P7 CiC] [P7 FC] [P7 Hip] [P14 blood]	Garay, 2013 [P0 Hip] [P14 CiC] [P14 FC] [P14 Hip]
	Post-weaning	Rose, 2017 [P1338]	Garay, 2013 [P30 blood] [P30 Hip] [P60 blood] [P60 CiC] [P60 FC] [P60 Hip] Rose, 2017 [P395]	Garay, 2013 [P30 CiC] [P30 FC]
MIP-1α	Prenatal	Arrode-Brusés, 2012 [G17]	Ehninger, 2014 [6.5 ​h]	Arrode-Brusés, 2012 [G16] Ehninger, 2014 [2 ​h]
	Pre-weaning	Garay, 2013 [P7 Hip]	Arsenault, 2014 Garay, 2013 [P0 CiC] [P0 FC] [P7 blood] [P7 CiC] [P7 FC] [P14 blood] [P14 CiC] [P14 FC] [P14 Hip]	Garay, 2013 [P0 blood] [P0 Hip]
	Post-weaning		Garay, 2013 [P30 blood] [P30 CiC] [P30 FC] [P60 blood] [P60 CiC] [P60 FC] [P60 Hip] Rose, 2017 [P1338]	Garay, 2013 [P30 Hip]
RANTES	Prenatal	Openshaw, 2019 Pratt, 2013	Arrode-Brusés, 2012 [G17] Ehninger, 2014 [2 ​h] [6.5 ​h]	
	Pre-weaning	Garay, 2013 [P0 blood] [P7 blood]	Arsenault, 2014 [blood] [brain] Garay, 2013 [P0 CiC] [P7 CiC] [P7 FC] [P7 Hip] [P14 blood] [P14 CiC] [P14 FC]	Garay, 2013 [P14 Hip]
	Post-weaning		Garay, 2013 [P30 blood] [P30 CiC] [P30 FC] [P30 Hip] [P60 blood] [P60 CiC] [P60 FC] [P60 Hip]	
GM-CSF	Prenatal	Pratt, 2013	Arrode-Brusés, 2012 [G17] Ehninger, 2014 [2 ​h] [6.5 ​h]	
	Pre-weaning	Garay, 2013 [P0 FC]	Garay, 2013 [P0 CiC] [P0 Hip] [P7 blood] [P7 CiC] [P7 FC] [P7 Hip] [P14 blood] [P14 Hip]	Garay, 2013 [P0 blood] [P14 CiC] [P14 FC]
	Post-weaning		Garay, 2013 [P30 blood] [P30 CiC] [P30 Hip] [P60 blood] [P60 CiC] [P60 FC] [P60 Hip] Rose, 2017 [P395] [P1338]	Garay, 2013 [P30 FC]
CXCL1	Prenatal	Horváth, 2019 Mueller, 2019 [batch 2] [batch 3] [batch 4] [batch HMW] [batch LMW]	Arrode-Brusés, 2012 [G17] Ehninger, 2014 [2 ​h] [6.5 ​h] Mueller, 2019 [batch 1]	
	Pre-weaning	Garay, 2013 [P7 blood] [P7 Hip]	Garay, 2013 [P0 blood] [P0 CiC] [P0 FC] [P7 CiC] [P7 FC] [P14 blood] [P14 FC] [P14 Hip]	Garay, 2013 [P0 Hip] [P14 CiC]
	Post-weaning		Garay, 2013 [P30 blood] [P30 CiC] [P30 FC] [P30 Hip] [P60 blood] [P60 CiC] [P60 FC] [P60 Hip]	

Entries indicated in blue represent protein expression data, all other entries represent gene expression data.

CeC = Cerebral cortex; Cer ​= ​Cerebellum; CiC ​= ​Cingulate cortex; CPu = Caudate putamen; CXCL = Chemokine (C-X-C motif) ligand; F= Female; FC = Frontal cortex; G ​= ​Gestational day; GM-CSF ​= ​Granulocyte-macrophage colony-stimulating factor; Hip ​= ​Hippocampus; HMW ​= ​high molecular weight; IFN = Interferon; IL = Interleukin; LMW ​= ​low molecular weight; M ​= ​Male; MCP ​= ​Monocyte chemoattractant protein; MIP ​= ​Macrophage inflammatory protein; NeC = Neocortex; P = Postnatal day; RANTES ​= ​Regulated on activation normal T cell expressed and secreted; TNF ​= ​Tumor necrosis factor.

### 3.5 Meta-analysis

Based on the criteria defined in the protocol, only four immune outcome parameters were eligible for meta-analysis: IL-1β, IL-6, IL-10 and TNF-α. As shown in Table 6, Table 7, Table 8, Table 9, 16 articles reported on the effects of maternal poly(I:C) on IL-1β protein concentrations in the offspring. These included 53 experiments using 785 animals. Twenty two articles reported on IL-6 protein concentrations, comprising 62 experiments using 902 animals. For IL-10, protein concentrations in offspring from mothers treated with poly(I:C) effect sizes were calculated for 32 experiments from 10 articles using a total of 422 animals. Analysis of TNF-α protein concentrations in the offspring was based on 17 articles, reporting 56 separate experiments using 826 animals.

#### 3.5.1 Forest plots

**Fig. 5:**
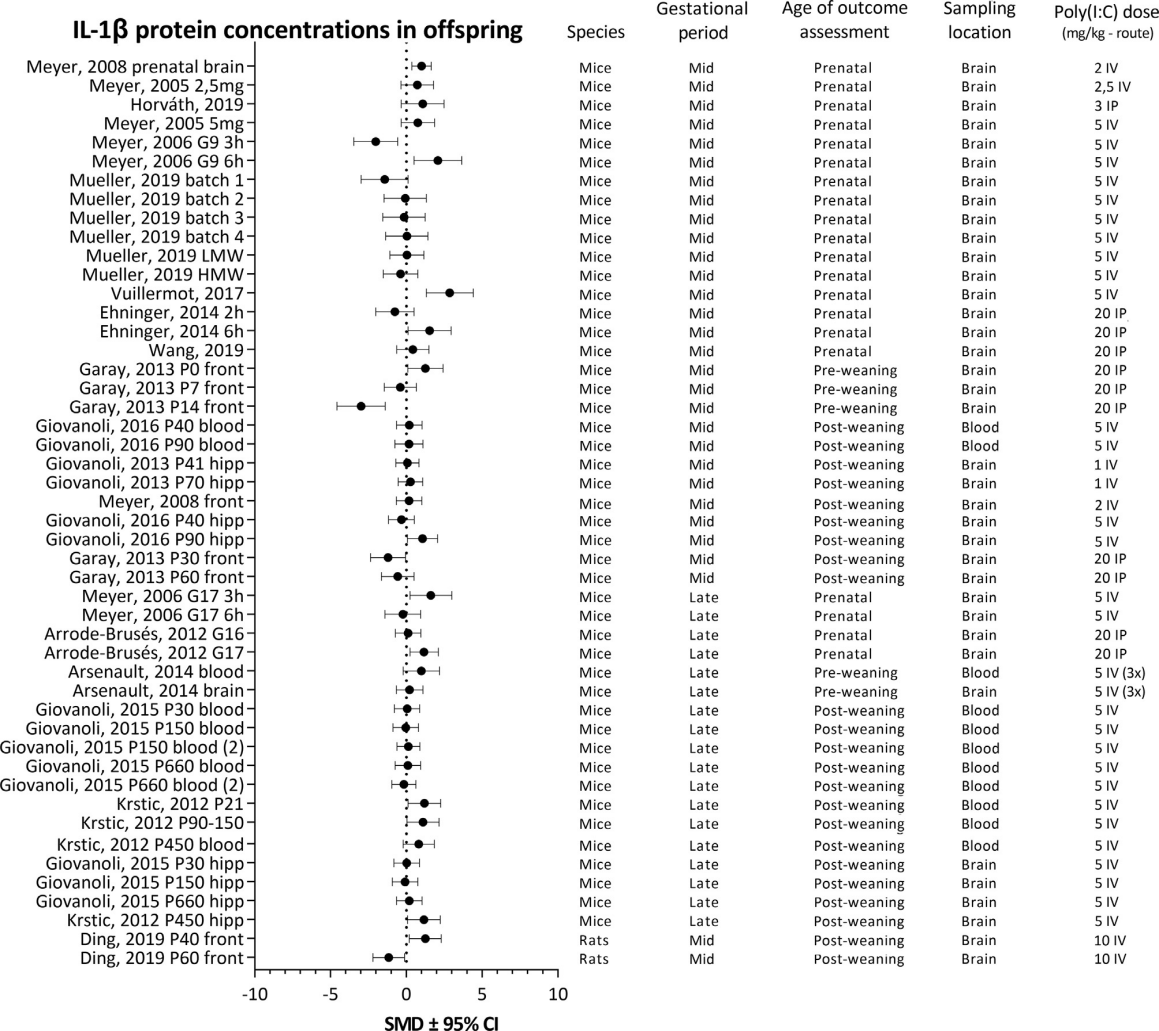
Forest plot of IL-1β protein concentrations with study characteristics.

**Fig. 6:**
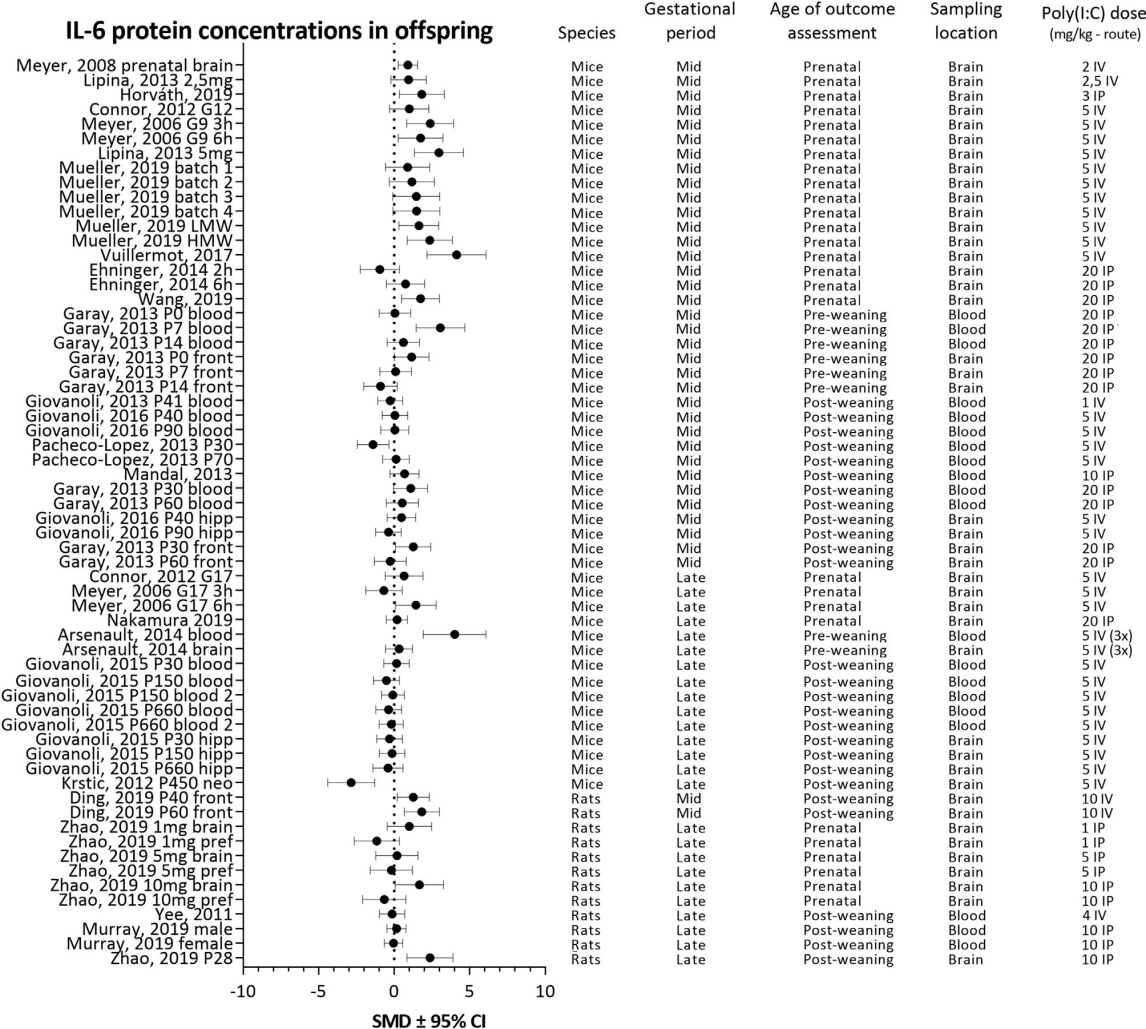
Forest plot of IL-6 protein concentrations with study characteristics.

**Fig. 7:**
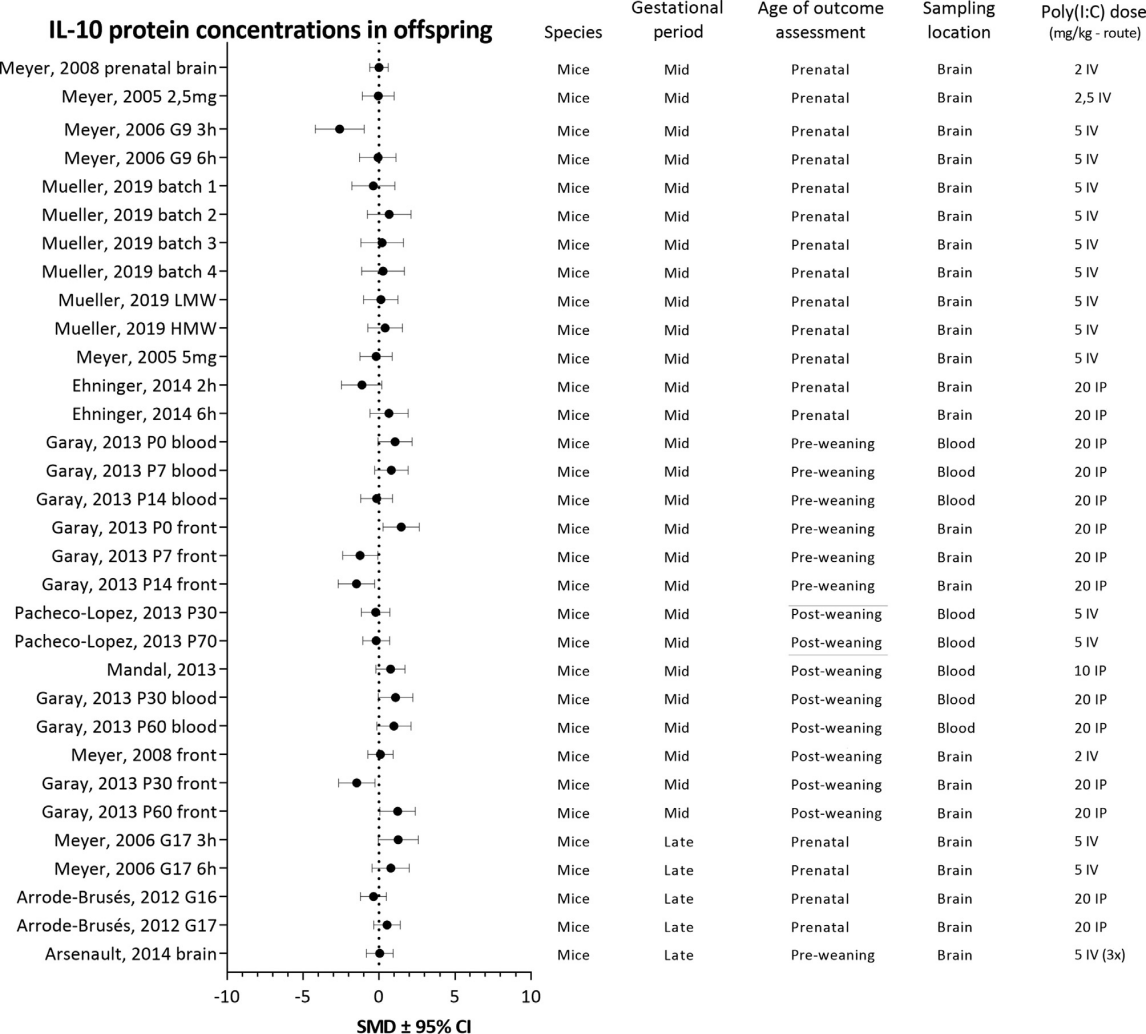
Forest plot of IL-10 protein concentrations with study characteristics.

**Fig. 8:**
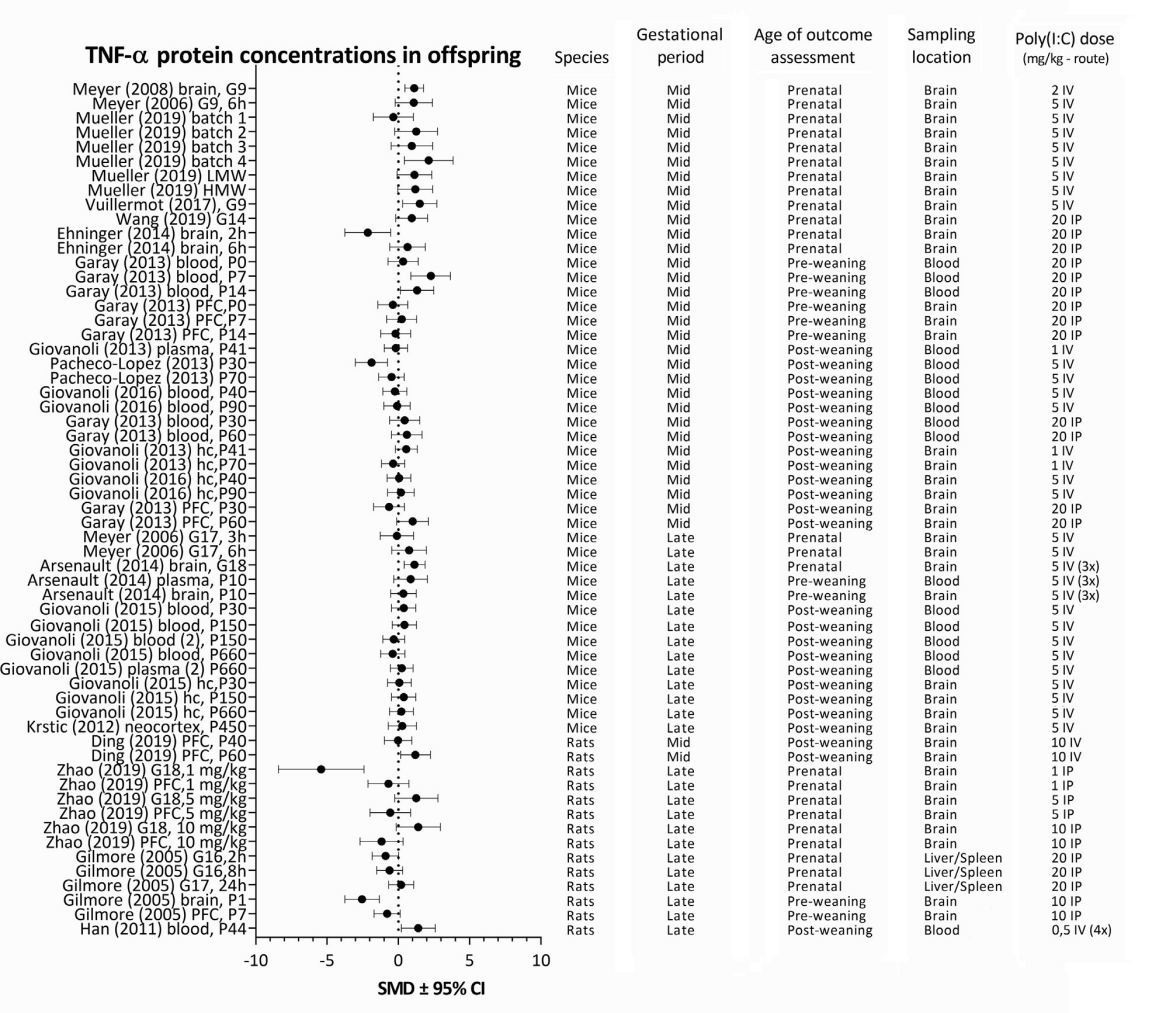
Forest plot of TNF-α protein concentrations with study characteristics.

For each analyzed cytokine, forest plots are shown with the addition of study characteristics in Fig. 5, Fig. 6, Fig. 7, Fig. 8. The heterogeneity for each parameter varied between I^2^ ​= ​50,4 and I^2^ ​= ​67,2%.

#### 3.5.2 Subgroup analyses

**Fig. 9:**
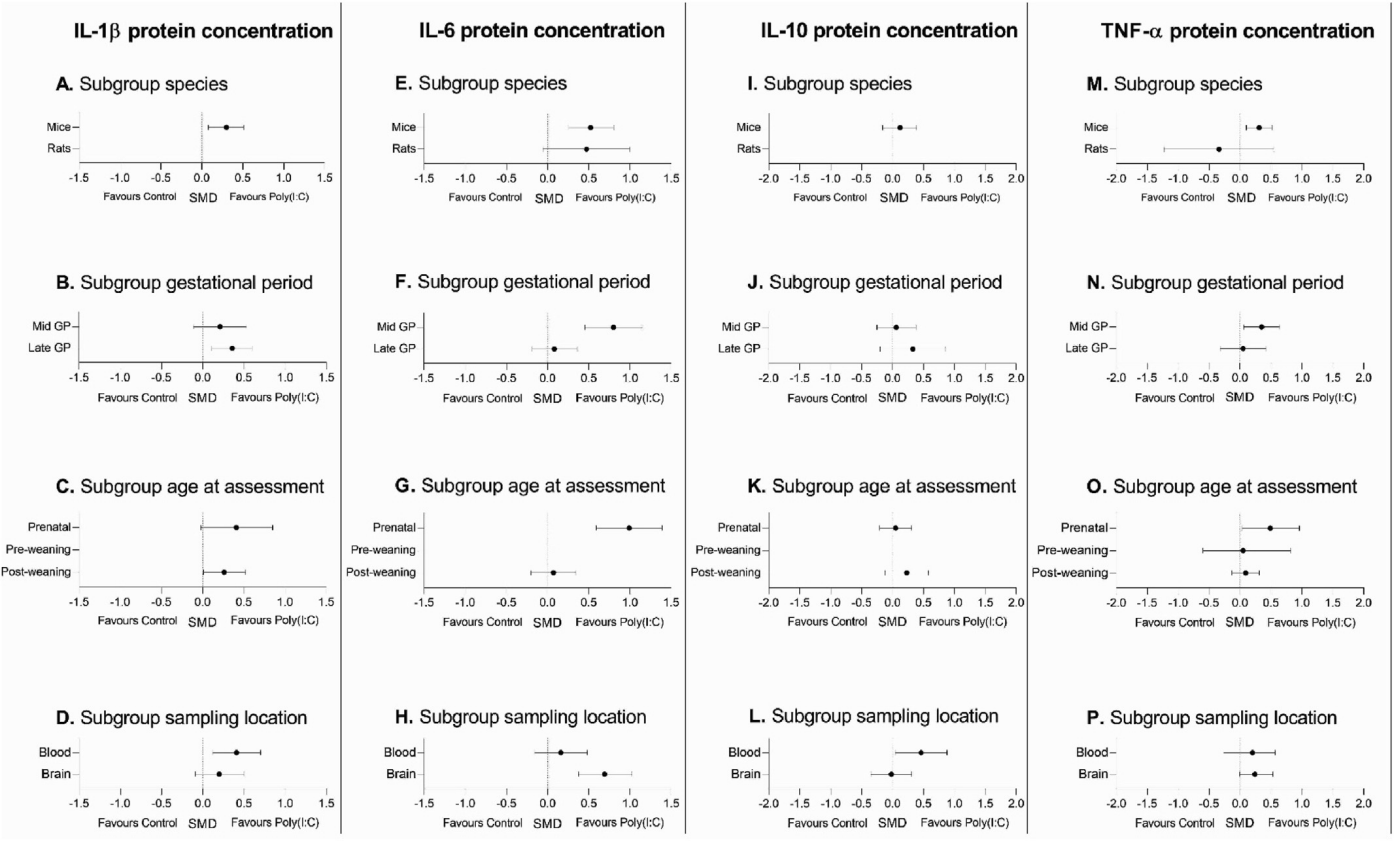
Visual representation of the SMD ±95% confidence interval for each subgroup analysis for each of the analyzed parameters. Missing dots represent subgroups that were not eligible for analysis.

Subgroup analyses were performed to check for significant effects within each of the predetermined methodological characteristics: species, gestational period, age of outcome assessment and sampling location. Fig. 9 visually represents the outcomes for each of the subgroup analyses.

##### 3.5.2.1 IL-1β protein concentrations

Pooling of all available experiments showed that maternal poly(I:C) injection resulted in an increase in IL-1β concentrations in the offspring (SMD 0.29 [0.07, 0.5]. Between study heterogeneity was I^2^ ​= ​58.2% (see Table 6).

**Table 6:** Subgroup analyses of IL-1β protein concentrations.

Subgroup	Articles (n)	Experiments (n)	Animals (n)	SMD [95% CI], I^2^
All studies	16	53	785	0.29 [0.07, 0.5], 58.2%
**Species**				
Mice	15	51	755	0.30 [0.08, 0.51], 50.4%
Rat	1	2	30	Not pooled
**Gestational period**				
Mid gestation	12	35	476	0.21 [-0.11, 0.53], 66.9%
Late gestation	5	18	309	0.36 [0.11, 0.60], 19.6%
**Ageatoutcome assessment**				
Prenatal	9	20	244	0.41 [-0.02, 0.85], 63.0%
Pre-weaning	2	7	88	Not pooled
Post-weaning	7	26	453	0.26 [0.01, 0.52], 47.0%
**Sampling location**				
Blood	5	16	256	0.41 [0.12, 0.70], 31.1%
Brain	16	37	529	0.20 [-0.09, 0.50], 64.6%

Subgroup analysis comparing species showed an effect of maternal poly(I:C) on IL-1β concentrations in mouse studies (See Fig. 9A). The data originating from the rat studies were not pooled, as this subgroup contained too few individual papers to be eligible for processing.

Poly(I:C) injection during late, but not mid gestation significantly increased IL-1β concentrations in the offspring. Subgroup analysis further indicated that the treatment effect in the subgroups did not differ significantly (see Fig. 9B).

Subgroup analysis comparing effects of maternal poly(I:C) in prenatal and post-weaning offspring showed a significant increase in IL-1β concentrations in the post-weaning, but not the prenatal subgroup (see Fig. 9C). However, the difference in treatment effect in these subgroups was not significantly different. There were insufficient articles to pool data of the pre-weaning subgroup.

When differences in sampling locations were assessed, subgroup analysis showed an increase in IL-1β concentrations in blood, but not in brain tissue of the offspring (see Fig. 9D). IL-1β concentrations however, did not differ significantly between sampling locations.

##### 3.5.2.2 IL-6 protein concentrations

Pooling data from all included experiments showed that maternal poly(I:C) injection enhanced IL-6 protein concentrations (SMD 0.51 [0.27, 0.75]. Between study heterogeneity was I^2^ ​= ​67.2% (see Table 7).

**Table 7:** Subgroup analyses of IL-6 protein concentration.

Subgroup	Articles (n)	Experiments (n)	Animals (n)	SMD [95% CI], I^2^
All studies	22	62	902	0.51 [0.27, 0.75], 67.2%
**Species**				
Mice	18	50	725	0.52 [0.25, 0.80], 69.3%
Rat	4	12	177	0.47 [-0.06, 1.00], 62%
**Gestational period**				
Mid gestation	15	37	490	0.8 [0.45, 1.15], 70.8%
Late gestation	9	25	412	0.08 [-0.19, 0.36], 46.6%
**Age at outcome assessment**				
Prenatal	11	27	300	0.99 [ 0.59, 1.39], 60.5%
Pre-weaning	2	8	100	Not pooled
Post-weaning	11	27	502	0.07 [-0.20, 0.34], 57.9%
**Sampling location**				
Blood	9	20	374	0.16 [-0.16, 0.48], 58.4%
Brain	17	42	528	0.69 [0.37, 1.02], 68.6%

Subgroup analysis for species showed an effect of maternal poly(I:C) on IL-6 concentrations in mice, but not rats. The effects on IL-6 concentrations in mice did not differ significantly from those in rats (see Fig. 9E).

Subgroup analysis for gestational period during which poly(I:C) was injected to the mothers showed that maternal poly(I:C) injection during mid gestation, but not late gestation, resulted in significant increases in IL-6 concentrations in the offspring (see Fig. 9F). Further analysis showed that the effect in the mid gestation group was significantly different from that in the late gestation group (ΔSMD 0.72 [0.27, 1.17], p ​= ​0.005).

Maternal poly(I:C) injection significantly increased IL-6 concentrations in the offspring before birth. Prenatal IL-6 concentrations were significantly higher when compared to IL-6 concentrations post-weaning (ΔSMD 0.92 [0.44, 1.40], p ​= ​0.0005). In the post-weaning group no increase in IL-6 concentrations was observed (see Fig. 9G). The number of articles in the pre-weaning group (n ​= ​2) was insufficient to conduct meaningful subgroup analyses.

Subgroup analyses showed a significant increase in IL-6 concentrations in brain tissue but not in blood. The difference between the two sampling locations in the offspring was not significantly different (see Fig. 9H).

##### 3.5.2.3 IL-10 protein concentrations

Pooling of all available data showed that overall maternal poly(I:C) treatment had no effect on IL-10 concentrations in the offspring (SMD 0.12 [-0.16, 0.39]). Heterogeneity was I^2^ ​= ​50.4% (see Table 8).

**Table 8:** Subgroup analyses of IL-10 protein concentrations.

Subgroup	Articles (n)	Experiments (n)	Animals (n)	SMD [95% CI], I^2^
All studies	10	32	422	0.12 [-0.16, 0.39], 50.4%
**Species**				
Mice	10	32	422	0.12 [-0.16, 0.39], 50.4%
Rats	0	0	0	Not pooled
**Gestational period**				
Mid gestation	8	27	374	0.06 [-0.25, 0.38], 53.7%
Late gestation	3	5	75	0.33 [-0.20, 0.85], 28.6%
**Ageatoutcome assessment**				
Prenatal	6	17	214	0.05 [-0.21, 0.32], 22.9%
Pre-weaning	2	7	90	Not pooled
Post-weaning	4	8	118	0.23 [-0.12, 0.58], 16.3%
**Sampling location**				
Blood	3	8	110	0.46 [0.04, 0.88], 24.8%
Brain	8	24	312	−0.02 [-0.35, 0.31], 53.2%

Subgroup analysis comparing species showed that maternal poly(I:C) had no significant effect on IL-10 concentrations in mice (see Fig. 9I). There were not enough studies to pool the data of the rat studies.

Subgroup analyses demonstrated no effect of maternal poly(I:C) on IL-10 protein concentrations for the subgroups gestational period of maternal poly(I:C) injection and age at outcome assessment (see Fig. 9J and K). There were insufficient articles to reliably analyze data of the pre-weaning subgroup.

Subgroup analysis showed an increase in IL-10 concentrations in blood, but not in brain tissue of the offspring. IL-10 concentrations however, did not differ significantly between these subgroups (see Fig. 9L).

##### 3.5.2.4 Subgroup analyses for TNF-a concentrations in the offspring

Pooling results from all available experiments showed that overall maternal poly(I:C) treatment enhanced TNF-α concentrations in the offspring (SMD 0.23 [0.002, 0.46]). Heterogeneity was 61.9% (see Table 9).

**Table 9:** Subgroup analyses of TNF-α protein concentrations.

Subgroup	Articles (n)	Experiments (n)	Animals (n)	SMD [95% CI], I^2^
All studies	17	56	826	0.23 [0.002, 0.46], 61.9%
**Species**				
Mouse	13	45	700	0.31 [0.10, 0.52], 49.8%
Rat	4	11	126	−0.34 [-1.23, 0.55], 80.6%
**Gestational period**				
Mid gestation	12	33	470	0.35 [0.06, 0.64], 59.1%
Late gestation	7	23	356	0.05 [-0.32, 0.42], 65.8%
**Ageatoutcome assessment**				
Prenatal	8	21	244	0.49 [0.03, 0.96], 63.9%
Pre-weaning	3	10	136	0.05[-0.60, 0.82], 76.8%
Post-weaning	8	25	446	0.09 [-0.13, 0.31], 33.3%
**Sampling location**				
Blood	7	17	281	0.20 [-0.27, 0.57], 59.4%
Brain	15	39	545	0.24 [-0.005, 0.53], 63.4%

Maternal poly(I:C) resulted in a significant increase in TNF-α concentrations in mouseoffspring, whereas no effect was found in rats. The effects in the two subgroups did not differ significantly (see Fig 9M).

TNF-α concentrations were enhanced in offspring from mothers injected during mid gestion, but not late gestation. The effects in these subgroups did however not differ significantly (see Fig 9N).

Subgroup analyses showed an increase in TNF-α concentrations in offspring when measured before birth, but not pre- or post-weaning. The effect observed in the prenatal subgroup did not differ significantly from the effect in pre-weaning and post-weaning subgroups (see Fig 9O).

Subgroup analyses showed no alteration in TNF-α concentrations in the subgroups for sampling location, nor an effect between these subgroups (see Fig 9P).

### 3.6 Sensitivity analysis

To assess the robustness of our findings, we performed two sensitivity analyses. Firstly, we excluded studies measuring cytokines in blood samples to test for confounding by sampling locations. Secondly, we controlled for studies that used a deviant poly(I:C) dosing regimen. To this purpose, studies that injected relatively low doses of poly(I:C), defined as 5 ​mg/kg IP or less and 2 ​mg/kg IV or less, as well as studies in which poly(I:C) was administered repeatedly were excluded.

The sensitivity analysis excluding blood samples had no significant effect on the magnitude or direction of the effect for any of the cytokines analyzed. The sensitivity analysis for dosing regimen had no significant effects on the effect sizes calculated for IL-6 and IL-10 in any of the subgroups. For IL-1β and TNF-α exclusion of these studies resulted in loss of significance for respectively the increase in IL-1β observed post-weaning, and the increase in TNF-α protein concentrations before birth.

### 3.7 Publication bias

**Fig. 10:**
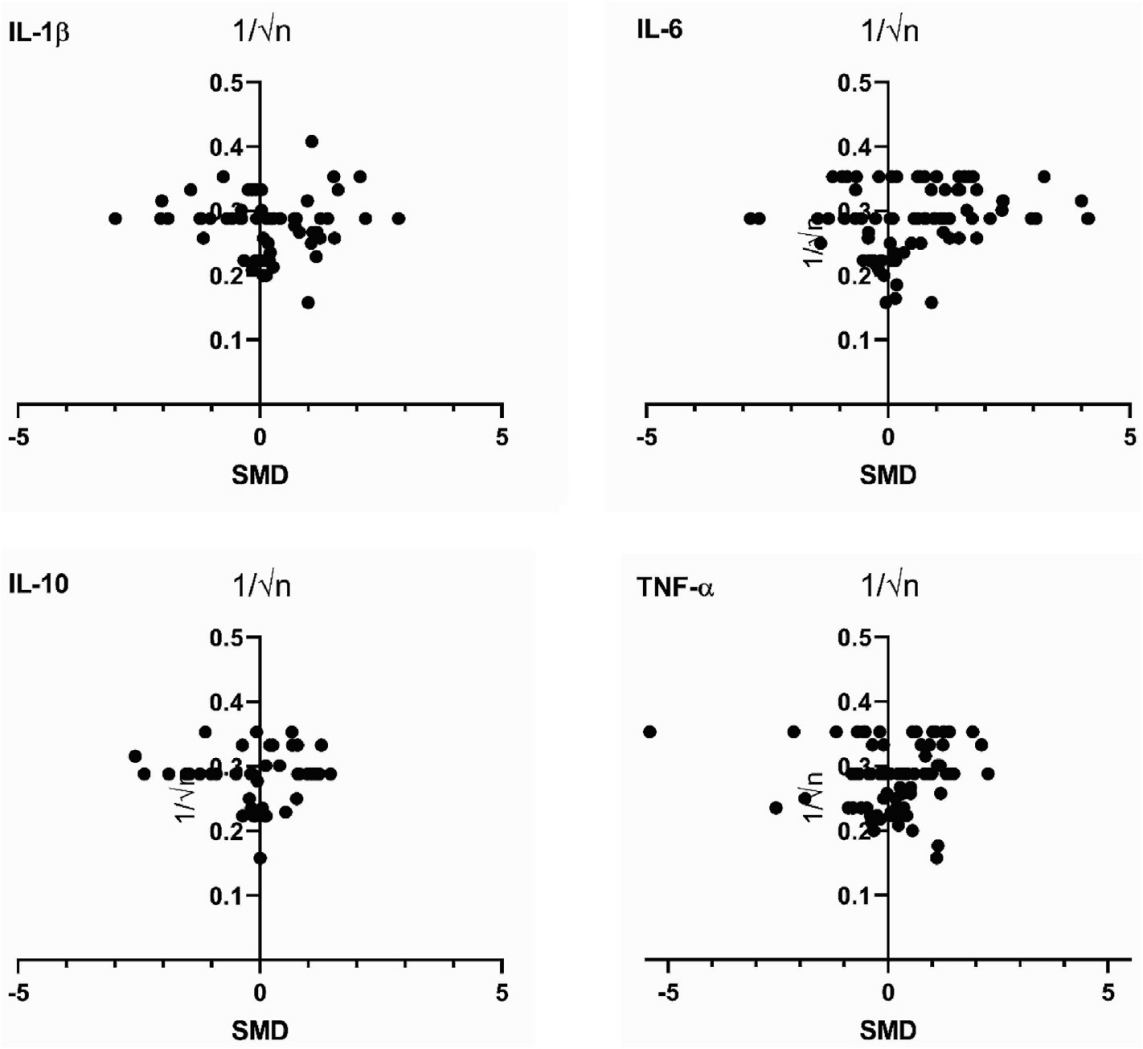
Funnel plots for the protein concentrations reported for IL-1β, IL-6, IL-10 and TNF-α.

Fig. 10 shows funnel plots for the parameters that were eligible for meta-analysis. Visual inspection of these funnel plots revealed varying shapes. IL-1β, IL10 and TNF-α showed no distinct funnel shape, symmetry or asymmetry. Funnel plots with sufficient data are expected to assume a funnel shape so that they may be interpreted. The lack of any discernible shapes for IL-1β, IL10 and TNF-α may indicate that their shapes are a product of chance rather than any form of publication bias and prevents them from being reliably interpreted. The funnel plot of IL-6 showed a distinct funnel shape with a small degree of asymmetry due to a lack of small studies with negative outcomes. This observation indicates the possibility of publication bias for IL-6.

## 4 Discussion

### 4.1 Effect of maternal poly(I:C) on offspring immune mediators

This systematic review is the first to analyze and compare immunological outcomes and study characteristics of the maternal poly(I:C) model. The descriptive tables and meta-analysis show an increase in IL-6 concentrations in offspring of mothers exposed to poly(I:C), which is in line with the current consensus in the field. It is understood that changes in cyto- and chemokine concentrations are a driving factor behind the effects of maternal immune activation on neurodevelopment (Estes and McAllister, 2016). Indeed, Smith and coworkers showed that injecting pregnant dams with IL-6 induced symptoms similar to those of maternal immune activation, while the administration of anti-IL-6 antibody could alleviate symptoms caused by poly(I:C) (Smith et al., 2007). This review confirms the notion of IL-6 playing a central role in the effects of maternal immune activation.

The observed increase in IL-6 protein concentrations was significantly more pronounced prenatally than post-weaning. Since the time between poly(I:C) exposure and sampling is, by definition, shorter in the prenatal group than it is in the pre- or post-weaning groups, this may suggest that the observed IL-6 response is a short-term rather than a long-term effect. This, in turn, raises the question if the observed immune response is of maternal or fetal origin. Maternal IL-6 is known to be able to reach the fetus in rats, but fetal cells are also capable of mounting a response of their own (Dahlgren et al., 2006). Either one, or both, could be responsible for increased IL-6 concentrations in the offspring following poly(I:C) injection.

An alternative explanation for the strong increase in IL-6 concentrations before birth is that the age of outcome assessment is confounded by sampling location. Unlike included pre- and post-weaning measurements, prenatal measurements were performed exclusively in brain, possibly due to technical difficulties involving fetal blood sampling. As such, it cannot be excluded that this difference in sampling locations may have contributed to the larger increase in IL-6 concentrations in prenatal samples. This notion is however not supported by the sensitivity analysis.

Another interesting observation is the lack of an effect in the remaining cytokines. Poly(I:C) is well known to trigger the release of several immune mediators, such as IL-1β, IL-6, TNF-α and IFN-ɣ (Alexopoulou et al., 2001; Gilmore et al., 2005; Voss et al., 2006). As a result, one would expect a short-term inflammatory response to poly(I:C) to constitute more immune mediators than just IL-6. One explanation for this discrepancy could be that the changes in cytokine concentrations occurring in the offspring are very brief and small. Since cytokines such as IL-1β, TNF-α and IFN-ɣ can stimulate the expression of IL-6 by neurons, astrocytes and microglia, IL-6 may locally reach relatively high concentrations and stand out (Gadient and Otten, 1997). Alternatively, the remaining cytokines might have actually been affected, but only under specific conditions. As is evident in our results and often the case in meta-analyses of animal studies, the data has a large degree of heterogeneity. For this reason, caution is warranted when drawing conclusions from the pooled overall effects. If a cytokine were to be increased under one condition and decreased under another, this effect would have been lost in the pooling of the outcomes. In addition, the number of included studies is limited. Therefore the subgroup analyses may not be sufficiently powered to identify subgroup differences. Additional research would provide the data required to elucidate the sources of this heterogeneity and help prove or disprove the effects that the experimental set-up may have on cytokine concentrations.

The absence of effect in immune mediators in pre- and post-weaning samples from offspring of poly(I:C)-exposed animals is at odds with the current literature on cytokines in human patients suffering from neurodevelopmental disorders. It is reported that schizophrenia patients with a first-episode psychosis were observed to have increased blood concentrations of IL-1β, IL-6, IL-12, IFN-ɣ, TNF-α, TGF-β and sIL-2R compared to healthy controls (Miller et al., 2011). In another meta-analysis patients with an autism spectrum disorder were reported to have increased blood concentrations of IL-1β, IL-6, IFN-ɣ and TNF-α (Saghazadeh et al., 2019). These findings suggest that neurodevelopmental disorders have a persistent immune component in humans. The fact that outcomes of the poly(I:C) model, which is used to model neurodevelopmental disorders, only points towards a limited and prenatal increase in cytokine concentrations is therefore noteworthy. There are several possible explanations for this discrepancy.

First and foremost, meta-analyses in humans measure in blood and cerebrospinal fluid, while studies using the poly(I:C) model tend to sample the brain. This means that the outcomes of this study and human meta-analyses cannot be directly compared. Secondly, the poly(I:C) model uses a very specific intervention to affect neurodevelopment, namely maternal immune activation. The human situation may be much more complex than this, involving genetic susceptibility and environmental stimuli as well, with maternal immune activation playing a role in only a fraction of the patients with neurodevelopmental disorders. If the inflammatory profile varies depending on the etiology of a case, then this may explain why the immune outcomes of the poly(I:C) model are not similar to those found in human meta-analyses. Indeed, there is some evidence that suggests the existence of inflammatory subgroups among patients with neurodevelopmental disorders, with approximately 40% of the schizophrenia patients exhibiting a high inflammatory status (Boerrigter et al., 2017; Martinuzzi et al., 2019; Purves-Tyson et al., 2019). Additional research is necessary to determine if maternal immune activation plays a significant role in the development of such subtypes and whether these subtypes correlate with psychopathological or neuropathological outcomes.

Lastly, the poly(I:C) model is a simplified model of neurodevelopmental disorders. Not only does this refer to the fact that the human nervous system is more complex than that of most other animals, but the model also lacks intricacies such as genetic susceptibility, dysbiosis and late-life environmental stimuli, also referred to as a “second hit”. It is likely that the development of a neurodevelopmental disorder is driven by a plethora of cumulative factors, of which maternal immune activation is only one possible example. As a result, changes induced by maternal immune activation alone may not fully represent the complex neurodevelopmental changes that result in mental disorders in humans.

Certain adjustments may be made to the model to further refine it and more closely represent the human situation, such as the inclusion of a second hit for animals primed by maternal immune activation. Examples of such factors could be late-life stress, late-life infection or genetic susceptibility (Estes and McAllister, 2016). The combination of poly(I:C) priming and juvenile stress has been attempted, though with varying results (Monte et al., 2017; Yee et al., 2011). Additional research into the type and timing of second hits may help move the field forward. It may also be worth broadening the scope of the field beyond the immune parameters that have been investigated by the studies included in this analysis. For example, the descriptive tables show that IFN-ɣ has only occasionally been studied in the context of the poly(I:C) model, even though meta-analyses in humans have identified IFN-ɣ as a cytokine of interest. Similarly, an increasing amount of evidence points towards a role for the complement system in neurodevelopmental disorders, yet only one study reported a measurement related to the complement system (Druart and Le Magueresse, 2019; Han et al., 2017). Finally, sampling blood and cerebrospinal fluid samples for cytokines could facilitate comparisons between preclinical and human studies.

### 4.2 Methodological characteristics

There were considerable differences in the methods used by the studies included in this systematic review. Since this diversity resulted in a limited amount of data for each individual characteristic, subgroup analysis was only possible for species, gestational period of poly(I:C) injection, sampling location and age of outcome assessment. Of these study characteristics, only prenatal versus post-weaning IL-6 concentrations and mid versus late gestation IL-6 concentrations showed significant differences and were identified as a source of heterogeneity, while no noteworthy differences were found in the remaining subgroups.

The lack and diversity of the data leaves a lot of uncertainty about the influence of a number of other characteristics that may still be partially responsible for the perceived heterogeneity, such as strains, poly(I:C) dose, administration route, animal vendor, maternal microbiome and analytical techniques. As more data becomes available in the field, future research may be able to explore the impact of these characteristics on the outcomes of the poly(I:C) model.

There were no measurements from animals which received the poly(I:C) injection during early gestation. Only one study reported injecting in this period, but the authors ultimately combined the results from early and mid-gestation (Rose et al., 2017). The lack of studies in which poly(I:C) is injected during early gestation could be due to the fact that the majority of the studies used mice and rats. These are stress-sensitive species with short pregnancies that are likely to terminate when exposed to a severe stressor early in a pregnancy (Arck et al., 1995; Joachim et al., 2001). This might explain why the one study to successfully inject during early gestation was also the only study using rhesus monkeys. Alternatively, researchers may have made the conscious choice to avoid the early gestation because the entirety of the murine neurodevelopment occurring *in utero* corresponds with the first and parts of the second trimester in humans (Workman et al., 2013). As a result, there would be relatively little practical use in studying the effects of poly(I:C) injection during the very first days of murine pregnancy and risking terminations, since the complete murine pregnancy represents the earlier human pregnancy. Regardless, there remains a gap in the data with respect to the effects of maternal immune activation during the earliest days of pregnancy.

Another gap lies in sampling from pre-weaning animals, from which there is very little data available compared to prenatal and post-weaning animals. This seems to be at least partially due to the simple fact that pre-weaning is a short period of time compared to post-weaning. Since the pre-weaning period is a crucial moment for neurodevelopment, this gap creates a lot of uncertainty about the effects of maternal poly(I:C) on immune mediators in the offspring during a developmental period in which alterations of immune mediators may be very impactful.

### 4.3 Strengths and limitations

To our knowledge, this is the first study that systematically and objectively assessed all published data on immune parameters in the offspring from mothers exposed to poly(I:C). The study protocol was preregistered with CAMARADES and a high degree of transparency is attained by providing open access to our data files (Supplementary files S1, S2). As such, this study provides a reliable basis for determining the direction of future research.

Unfortunately, the amount of available evidence was limited, the quality of reporting in included papers was generally low and heterogeneity between studies was too high to draw strong conclusions about the effects of maternal poly(I:C). The study was, however, able to successfully assess the diversity of study characteristics and elucidate some sources of heterogeneity in the field. Given the limited amount of data available, certain concessions had to be made to process the data as well, such as pooling different brain areas. With these concessions, valuable insights might have been lost.

The risk of bias analysis showed that the quality of reporting is low and measures taken to prevent bias in the included papers are largely unclear. Since this analysis is based on what is reported in the papers, it is unknown whether this rating shows if no measures were taken to reduce bias, or if authors merely failed to report their measures to reduce bias. Both bias prevention as well as reporting are essential to good scientific practice, however, which makes this outcome a reason for concern either way. The most commonly observed bias was the unit-of-analysis error, which always concerned a risk of litter effect due to using individual animals instead of litters as a unit of analysis.

In addition, visual inspection of the funnel plots revealed a small degree of asymmetry for IL-6 concentrations, which might indicate the underreporting of negative results. This could be partially caused by the already widely accepted idea that IL-6 is the driving factor behind the effects of maternal immune activation. It is worth noting that these funnel plots only represent a small and heterogenous segment of the total body of evidence, not including gene expression and outcomes below the detection limits of analysis techniques. As a result, they may not be representative for the body of evidence as a whole. Despite this limitation, the possible presence of publication bias weakens the reliability of conclusions based on pooled SMDs.

Finally, not all missing data and information could be retrieved from the respective authors. This, in combination with the aforementioned poor reporting and possible bias, may lead to misconceptions and the loss of valuable data, which in turn may lead to redundant studies or unnecessary duplications. We therefore strongly suggest the use of preregistration and publication guidelines to optimize study quality, such as the recently issued guidelines specific to maternal immune activation studies (Kentner et al., 2019).

### 4.4 Conclusion

The currently available evidence points towards increased IL-6 protein concentrations in offspring of mothers exposed to poly(I:C) which are most pronounced prenatally. Results further imply that effects on IL-6 concentrations are strongest if poly(I:C) is administered during mid gestation. Maternal poly(I:C) induced changes in IL-1β, Il-10 and TNF-α concentrations could not be associated with age of offspring, gestational period or sampling location. These findings imply that maternal poly(I:C) triggers a short-term immune response mediated by IL-6, while not causing consistent long-term changes in cytokine concentrations. Given the identified paucity of data regarding cyto- and chemokines other than IL-1β, IL-6 IL-10 or TNF-α, we strongly encourage future research to also address other immune mediators that could be involved in maternal immune activation. As such, the systematic map that this review provides may assist in the design and direction of future research and may help prevent unnecessary duplications of studies.

## Declaration of competing interest

M.M. Tiemessen is an employee at Danone Nutricia Research. The other authors declare no conflict of interest.

## References

[bib1] Alexopoulou L., Holt A.C., Medzhitov R., Flavell R.A. (2001 Oct 18). Recognition of double-stranded RNA and activation of NF-kappaB by Toll-like receptor 3. Nature.

[bib2] Arck P.C., Merali F.S., Stanisz A.M., Stead R.H., Chaouat G., Manuel J., Clark D.A. (1995 Oct). Stress-induced murine abortion associated with substance P-dependent alteration in cytokines in maternal uterine decidua. Biol. Reprod..

[bib3] Arrode-Brusés G., Brusés J.L. (2012 Apr 30). Maternal immune activation by poly(I:C) induces expression of cytokines IL-1β and IL-13, chemokine MCP-1 and colony stimulating factor VEGF in fetal mouse brain. J. Neuroinflammation.

[bib4] Arsenault D., St-Amour I., Cisbani G., Rousseau L., Cicchetti F. (2014 May). The different effects of LPS and poly I:C prenatal immune challenges on the behavior, development and inflammatory responses in pregnant mice and their offspring. Brain Behav. Immun..

[bib6] Boerrigter D., Weickert T.W., Lenroot R., O’Donnell M., Galletly C., Liu D., Burgess M., Cadiz R., Jacomb I., Catts V.S., Fillman S.G., Weickert C.S. (2017 Sep 18). Using blood cytokine measures to define high inflammatory biotype of schizophrenia and schizoaffective disorder. J. Neuroinflammation.

[bib7] Bradbury T.N., Miller G.A. (1985). Season of birth in schizophrenia: a review of evidence, methodology, and etiology. Psychol. Bull..

[bib8] Brown A.S., Cohen P., Greenwald S., Susser E. (2000 Mar). Nonaffective psychosis after penatal exposure to rubella. Am. J. Psychiatr..

[bib9] Brown A.S., Begg M.D., Gravenstein S., Schaefer C.A., Wyatt R.J., Bresnahan M., Babulas V.P., Susser E.S. (2004). Serologic evidence of prenatal influenza in the etiology of schizophrenia. Arch. Gen. Psychiatr..

[bib10] Brown A.S., Schaefer C.A., Quesenberry C.P., Liu L., Babulas V.P., Susser E.S. (2005 Apr). Maternal exposure to toxoplasmosis and risk of schizophrenia in adult offspring. Am. J. Psychiatr..

[bib11] Brown A.S., Sourander A., Hinkka-Yli-Salomäki S., McKeague I.W., Sundvall J., Surcel H.M. (2014 Feb). Elevated maternal C-reactive protein and autism in a national birth cohort. Mol. Psychiatr..

[bib12] CAMARADES (2014). http://www.dcn.ed.ac.uk/camarades/research.html#protocols.

[bib13] Canetta S., Sourander A., Surcel H.M., Hinkka-Yli-Salomäki S., Leiviskä J., Kellendonk C., McKeague I.W., Brown A.S. (2014 Sep). Elevated maternal C-reactive protein and increased risk of schizophrenia in a national birth cohort. Am. J. Psychiatr..

[bib14] Clark S.M., Notarangelo F.M., Li X., Chen S., Schwarcz R., Tonelli L.H. (2019 Mar 8). Maternal immune activation in rats blunts brain cytokine and kynurenine pathway responses to a second immune challenge in early adulthood. Prog. Neuro-Psychopharmacol. Biol. Psychiatry.

[bib15] Connor C.M., Dincer A., Straubhaar J., Galler J.R., Houston I.B., Akbarian S. (2012 Sep). Maternal immune activation alters behavior in adult offspring, with subtle changes in the cortical transcriptome and epigenome. Schizophr. Res..

[bib16] Corradini I., Focchi E., Rasile M., Morini R., Desiato G., Tomasoni R., Lizier M., Ghirardini E., Fesce R.G., Morone D., Barajon I., Antonucci F., Pozzi D., Matteoli M. (2018 Apr 15). Maternal immune activation delays excitatory-to-inhibitory gamma-aminobutyric acid switch in offspring. Biol. Psychiatr..

[bib17] Dahlgren J., Samuelsson A., Jansson T., Holmäng A. (2006). Interleukin-6 in the maternal circulation reaches the rat fetus in mid-gestation. Pediatr. Res..

[bib18] Deverman B.E., Patterson P.H. (2009 Oct 15). Cytokines and CNS development. Neuron.

[bib19] Ding S., Hu Y., Luo B., Cai Y., Hao K., Yang Y., Zhang Y., Wang X., Ding M., Zhang H., Li W., Lv L. (2019 Mar 5). Age-related changes in neuroinflammation and prepulse inhibition in offspring of rats treated with Poly I:C in early gestation. Behav. Brain Funct..

[bib20] Druart M., Le Magueresse C. (2019). Emerging roles of complement in psychiatric disorders. Front. Psychiatr..

[bib21] Duchatel R.J., Meehan C.L., Harms L.R., Michie P.T., Bigland M.J., Smith D.W., Walker F.R., Jobling P., Hodgson D.M., Tooney P.A. (2018 Aug). Late gestation immune activation increases IBA1-positive immunoreactivity levels in the corpus callosum of adult rat offspring. Psychiatr. Res..

[bib22] Ehninger D. (2014). Tsc2 haploinsufficiency has limited effects on fetal brain cytokine levels during gestational immune activation. Autism Res Treat.

[bib23] Estes M.L., McAllister A.K. (2016). Maternal immune activation: implications for neuropsychiatric disorders. Science.

[bib24] Gadient R.A., Otten U.H. (1997). Interleukin-6 (IL-6)--a molecule with both beneficial and destructive potentials. Prog. Neurobiol..

[bib25] Garay P.A., Hsiao E.Y., Patterson P.H., McAllister A.K. (2013 Jul). Maternal immune activation causes age- and region-specific changes in brain cytokines in offspring throughout development. Brain Behav. Immun..

[bib26] German Centre for the Protection of Laboratory Animals (26th September 2019). https://www.animalstudyregistry.org/asr_web/index.action.

[bib27] Gilmore J.H., Jarskog L.F., Vadlamudi S. (2005). Maternal poly I:C exposure during pregnancy regulates TNFα, BDNF, and NGF expression in neonatal brain and the maternal–fetal unit of the rat. J. Neuroimmunol..

[bib28] Giovanoli S., Engler H., Engel A., Richetto J., Voget M., Willi R., Winter C., Riva M.A., Mortensen P.B., Feldon J., Schedlowski M., Meyer U. (2013 Mar 1). Stress in puberty unmasks latent neuropathological consequences of prenatal immune activation in mice. Science.

[bib29] Giovanoli S., Notter T., Richetto J., Labouesse M.A., Vuillermot S., Riva M.A., Meyer U. (2015 Nov 25). Late prenatal immune activation causes hippocampal deficits in the absence of persistent inflammation across aging. J. Neuroinflammation.

[bib30] Giovanoli S., Weber-Stadlbauer U., Schedlowski M., Meyer U., Engler H. (2016 Jul). Prenatal immune activation causes hippocampal synaptic deficits in the absence of overt microglia anomalies. Brain Behav. Immun..

[bib31] Han X., Li N., Meng Q., Shao F., Wang W. (2011). Maternal immune activation impairs reversal learning and increases serum tumor necrosis factor-α in offspring. Neuropsychobiology.

[bib32] Han M., Zhang J., Hashimoto K. (2017 Feb 28). Increased levels of C1q in the prefrontal cortex of adult offspring after maternal immune activation: prevention by 7,8-dihydroxyflavone. Clin. Psychopharmacol. Neurosci..

[bib33] Hollins S.L., Brock L., Barreto R., Harms L., Dunn A., Garcia-Sobrinho P., Bruce J., Dickson P.W., Walker M.M., Keely S., Hodgson D.M. (2018). A rodent model of anxiety: the effect of perinatal immune challenges on gastrointestinal inflammation and integrity. Neuroimmunomodulation.

[bib34] Hooijmans C.R., Rovers M.M., de Vries R.B.M., Leenaars M., Ritskes-Hoitinga M., Langendam M.W. (2014). SYRCLE’s risk of bias tool for animal studies. BMC Med. Res. Methodol..

[bib35] Horváth G., Otrokocsi L., Beko K., Baranyi M., Kittel A., Fritz-Ruenes P.A., Sperlágh B. (2019 Mar 27). P2X7 receptors drive poly(I:C) induced autism-like behavior in mice. J. Neurosci..

[bib36] Hu Y., Hong X., Yang X., Ma R., Wang X., Zhang J., Feng Q., Li X., Sun D., Li X., Wan H., Li T., Wang Q., Ke D., Wang J., Liu G. (2019 Jun 1). Inflammation-dependent ISG15 upregulation mediates MIA-induced dendrite damages and depression by disrupting NEDD4/Rap2A signalling. Biochim. Biophys. Acta (BBA) - Mol. Basis Dis..

[bib37] Hui C.W., St-Pierre A., El Hajj H., Remy Y., Hébert S.S., Luheshi G.N., Srivastava L.K., Tremblay M. (2018 Feb 8). Prenatal immune challenge in mice leads to partly sex-dependent behavioral, microglial, and molecular abnormalities associated with schizophrenia. Front. Mol. Neurosci..

[bib38] Joachim R.A., Hildebrandt M., Oder J., Klapp B.F., Arck P.C. (2001). Murine stress-triggered abortion is mediated by increase of CD8^+^ TNF-α^+^ decidual cells via substance P. Am. J. Reprod. Immunol..

[bib39] Kentner A.C., Bilbo S.D., Brown A.S., Hsiao E.Y., McAllister A.K., Meyer U., Pearce B.D., Pletnikov M.V., Yolken R.H., Bauman M.D. (2019). Maternal immune activation: reporting guidelines to improve the rigor, reproducibility, and transparency of the model. Neuropsychopharmacology.

[bib40] Krstic D., Madhusudan A., Doehner J., Vogel P., Notter T., Imhof C., Manalastas A., Hilfiker M., Pfister S., Schwerdel C., Riether C., Meyer U., Knuesel I. (2012 Jul 2). Systemic immune challenges trigger and drive Alzheimer-like neuropathology in mice. J. Neuroinflammation.

[bib86] Linehan M.M., Dickey T.H., Molinari E.S., Fitzgerald M.E., Potapova O., Iwasaki A, Pyle A.M. (2018 Feb 21). A minimal RNA ligand for potent RIG-I activation in living mice. Science advances.

[bib41] Lipina T.V., Zai C., Hlousek D., Roder J.C., Wong A.H.C. (2013 May 1). Maternal immune activation during gestation interacts with Disc1 point mutation to exacerbate schizophrenia-related behaviors in mice. J. Neurosci..

[bib42] Mandal M., Donelly R., Elkabes S., Zhang P., Davini D., David B.T., Ponzio N.M. (2013 Oct). Maternal immune stimulation during pregnancy shapes the immunological phenotype of offspring. Brain Behav. Immun..

[bib43] Martinuzzi E., Barbosa S., Daoudlarian D., Wafa Bel Haj A., Gilet C., Fillatre L., Khalfallah O., Troudet R., Jamain S., Fond G., Sommer I., Leucht S., Dazzan P., McGuire P., Arango C., Diaz-Caneja C.M., Fleischhacker W., Rujescu D., Glenthøj B., Winter I., Kahn R.S., Yolken R., Lewis S., Drake R., Davidovic L., Leboyer M., Glaichenhaus N., OPTiMiSE Study Group (2019 Jan 17). Stratification and prediction of remission in first-episode psychosis patients: the OPTiMiSE cohort study. Transl. Psychiatry.

[bib44] Mattei D., Djodari-Irani A., Hadar R., Pelz A., Fernandez de Cossío L., Goetz T., Matyash M., Kettenmann H., Winter C., Wolf S.A. (2014 May). Minocycline rescues decrease in neurogenesis, increase in microglia cytokines and deficits in sensorimotor gating in an animal model of schizophrenia. Brain Behav. Immun..

[bib45] McAfoose J., Baune B.T. (2009 Mar). Evidence for a cytokine model of cognitive function. Neurosci. Biobehav. Rev..

[bib46] Meyer U., Feldon J., Schedlowski M., Yee B.K. (2005). Towards an immuno-precipitated neurodevelopmental animal model of schizophrenia. Neurosci. Biobehav. Rev..

[bib47] Meyer U., Nyffeler M., Engler A., Urwyler A., Schedlowski M., Knuesel I., Yee B.K., Feldon J. (2006 May 3). The time of prenatal immune challenge determines the specificity of inflammation-mediated brain and behavioral pathology. J. Neurosci..

[bib48] Meyer U., Murray P.J., Urwyler A., Yee B.K., Schedlowski M., Feldon J. (2008 Feb). Adult behavioral and pharmacological dysfunctions following disruption of the fetal brain balance between pro-inflammatory and IL-10-mediated anti-inflammatory signalling. Mol. Psychiatr..

[bib49] Miller B.J., Buckley P., Seabolt W., Mellor A., Kirkpatrick B. (2011). Meta-analysis of cytokine alterations in schizophrenia: clinical status and antipsychotic effects. Biol. Psychiatr..

[bib50] Missault S., Van den Eynde K., Vanden Berghe W., Fransen E., Weeren A., Timmermans J.P., Kumar-Singh S., Dedeurwaerdere S. (2014 Nov). The risk for behavioural deficits is determined by the maternal immune response to prenatal immune challenge in a neurodevelopmental model. Brain Behav. Immun..

[bib51] Mortensen P.B., Nørgaard-Pedersen B., Waltoft B.L., Sørensen T.L., Hougaard D., Torrey E.F., Yolken R.H. (2007 Mar). Toxoplasma gondii as a risk factor for early-onset schizophrenia: analysis of filter paper blood samples obtained at birth. Biol. Psychiatr..

[bib52] Mortensen P.B., Pedersen C.B., Hougaard D.M., Nørgaard-Petersen B., Mors O., Børglum A.D., Yolken R.H. (2010 Sep). A Danish national birth cohort study of maternal HSV-2 antibodies as a risk factor for schizophrenia in their offspring. Schizophr. Res..

[bib53] Mueller F.S., Richetto J., Hayes L.N., Zambon A., Pollak D.D., Sawa A., Meyer U., Weber-Stadlbauer U. (2019 Aug). Influence of poly(I:C) variability on thermoregulation, immune responses and pregnancy outcomes in mouse models of maternal immune activation. Brain Behav. Immun..

[bib54] Murray K.N., Edye M.E., Manca M., Vernon A.C., Oladipo J.M., Fasolino V., Harte M.K., Mason V., Grayson B., McHugh P.C., Knuesel I., Prinssen E.P., Hager R., Neill J.C. (2019 Jan). Evolution of a maternal immune activation (mIA) model in rats: early developmental effects. Brain Behav. Immun..

[bib55] Nakamura J.P., Schroeder A., Hudson M., Jones N., Gillespie B., Du X., Notaras M., Swaminathan V., Reay W.R., Atkins J.R., Green M.J., Carr V.J., Cairns M.J., Sundram S., Hill R.A. (2019 Oct). The maternal immune activation model uncovers a role for the Arx gene in GABAergic dysfunction in schizophrenia. Brain Behav. Immun..

[bib56] Oh-Nishi A., Koga K., Maeda T., Suhara T. (2016 Dec). A possible serologic biomarker for maternal immune activation-associated neurodevelopmental disorders found in the rat models. Neurosci. Res..

[bib57] Openshaw R.L., Kwon J., McColl A., Penninger J.M., Cavanagh J., Pratt J.A., Morris B.J. (2019). JNK signalling mediates aspects of maternal immune activation: importance of maternal genotype in relation to schizophrenia risk. J. Neuroinflammation.

[bib58] Ozawa K., Hashimoto K., Kishimoto T., Shimizu E., Ishikura H., Iyo M. (2006). Immune activation during pregnancy in mice leads to dopaminergic hyperfunction and cognitive impairment in the offspring: a neurodevelopmental animal model of schizophrenia. Biol. Psychiatr..

[bib59] Pacheco-López G., Giovanoli S., Langhans W., Meyer U. (2013 Mar). Priming of metabolic dysfunctions by prenatal immune activation in mice: relevance to schizophrenia. Schizophr. Bull..

[bib60] Parboosing R., Bao Y., Shen L., Schaefer C.A., Brown A.S. (2013 Jul). Gestational influenza and bipolar disorder in adult offspring. JAMA Psychiatry.

[bib61] Pratt L., Ni L., Ponzio N.M., Jonakait G.M. (2013 Jul 22). Maternal inflammation promotes fetal microglial activation and increased cholinergic expression in the fetal basal forebrain: role of interleukin-6. Pediatr. Res..

[bib62] Preclinicaltrials.eu (26th September 2019). https://preclinicaltrials.eu.

[bib63] Purves-Tyson T.D., Weber-Stadlbauer U., Richetto J., Rothmond D.A., Labouesse M.A., Polesel M., Robinson K., Weickert C.S., Meyer U. (2019 Jun 5). Increased levels of midbrain immune-related transcripts in schizophrenia and in murine offspring after maternal immune activation. Mol. Psychiatr..

[bib64] Ratnayake U., Quinn T., LaRosa D.A., Dickinson H., Walker D.W. (2014). Prenatal exposure to the viral mimetic poly I:C alters fetal brain cytokine expression and postnatal behaviour. Dev. Neurosci..

[bib65] Reitsema L.J., Partrick K.A., Muir A.B. (2015). Inter-individual variation in weaning among rhesus macaques (macaca mulatta): serum stable isotope indicators of suckling duration and lactation. Am. J. Primatol..

[bib66] Rose D.R., Careaga M., van de Water J., McAllister K., Bauman M.D., Ashwood P. (2017 Jul). Long-term altered immune responses following fetal priming in a non-human primate model of maternal immune activation. Brain Behav. Immun..

[bib67] Saghazadeh A., Ataeinia B., Keynejad K., Abdolalizadeh A., Hirbod-Mobarakeh A., Rezaei N. (2019). A meta-analysis of pro-inflammatory cytokines in autism spectrum disorders: effects of age, gender, and latitude. J. Psychiatr. Res..

[bib68] Santos Monte A., Ferreira Mello B.S., Moreira Borella V.C., da Silva Araujo T., Rodrigues da Silva F.S., de Sousa F.C.S., Pinheiro de Oliveira A.C., Severino Gama C., Seeman M.V., Mendes Vasconcelos S.M., Freitas De Lucena D., Macêdo D. (2017 Jul 28). Two-hit model of schizophrenia induced by neonatal immune activation and peripubertal stress in rats: study of sex differences and brain oxidative alterations. Behav. Brain Res..

[bib69] Smith S.E.P., Li J., Garbett K., Mirnics K., Patterson P.H. (2007). Maternal immune activation alters fetal brain development through interleukin-6. J. Neurosci..

[bib70] Solek C.M., Farooqi N., Verly M., Lim T.K., Ruthazer E.S. (2018 Apr). Maternal immune activation in neurodevelopmental disorders. Dev. Dynam..

[bib71] Takeuchi O., Akira S. (2007). Recognition of viruses by innate immunity. Immunol. Rev..

[bib72] Torrey E.F., Miller J., Rawlings R., Yolken R.H. (1997 Nov 7). Seasonability of births in schizophrenia and bipolar disorder: a review of the literature. Schizophr. Res..

[bib73] Tsukada T., Simamura E., Shimada H., Arai T., Higashi N., Akai T., Iizuka H., Hatta T. (2015 Jun 4). The suppression of maternal-fetal leukemia inhibitory factor signal relay pathway by maternal immune activation impairs brain development in mice. PLoS One.

[bib74] Volk D.W., Chitrapu A., Edelson J.R., Roman K.M., Moroco A.E., Lewis D.A. (2015 Nov 1). Molecular mechanisms and timing of cortical immune activation in schizophrenia. Am. J. Psychiatr..

[bib75] Volk D.W., Moroco A.E., Roman K.M., Edelson J.R., Lewis D.A. (2019 Jan 1). The role of the nuclear factor-κb transcriptional complex in cortical immune activation in schizophrenia. Biol. Psychiatr..

[bib76] Voss T., Rummel C., Gerstberger R., Hübschle T., Roth J. (2006). Fever and circulating cytokines induced by double-stranded RNA in Guinea pigs: dependence on the route of administration and effects of repeated injections. Acta Physiol..

[bib77] Vuillermot S., Luan W., Meyer U., Eyles D. (2017 Mar 7). Vitamin D treatment during pregnancy prevents autism-related phenotypes in a mouse model of maternal immune activation. Mol. Autism..

[bib78] Wang X., Yang J., Zhang H., Yu J., Yao Z. (2019 Apr). Oral probiotic administration during pregnancy prevents autism-related behaviors in offspring induced by maternal immune activation via anti-inflammation in mice. Autism Res..

[bib79] Workman A.D., Charvet C.J., Clancy B., Darlington R.B., Finlay B.L. (2013 Apr 24). Modeling transformations of neurodevelopmental sequences across mammalian species. J. Neurosci..

[bib80] Wu W., Adams C.E., Stevens K.E., Chow K., Freedman R., Patterson P.H. (2015 May). The interaction between maternal immune activation and alpha 7 nicotinic acetylcholine receptor in regulating behaviors in the offspring. Brain Behav. Immun..

[bib81] Yee N., Ribic A., Coenen de Roo C., Fuchs E. (2011 Oct 10). Differential effects of maternal immune activation and juvenile stress on anxiety-like behaviour and physiology in adult rats: No evidence for the “double-hit hypothesis”. Behav. Brain Res..

[bib82] Zhao Q., Wang Q., Wang J., Tang M., Huang S., Peng K., Han Y., Zhang J., Liu G., Fang Q., You Z. (2019 May). Maternal immune activation-induced PPARγ-dependent dysfunction of microglia associated with neurogenic impairment and aberrant postnatal behaviors in offspring. Neurobiol. Dis..

[bib83] Zuckerman L., Weiner I. (2005). Maternal immune activation leads to behavioral and pharmacological changes in adult offspring. J. Psychiatr. Res..

[bib84] Zuckerman L., Rehavi M., Nachman R., Weiner I. (2003). Immune activation during pregnancy in rats leads to a postpubertal emergence of disrupted latent inhibition, dopaminergic hyperfunction, and altered limbic morphology in the offspring: a novel neurodevelopmental model of schizophrenia. Neuropsychopharmacology.

[bib85] Zwetsloot P.P., van der Naald M., Sena E.S., Howells D.W., IntHout J., de Groot J.A.H., Chamuleau S.A.J., Macleod M.R., Wever K.E. (2017). Standardized mean differences cause funnel plot distortion in publication bias assessments. Elife.

