# Supplementary material for: The poly(I:C)-induced maternal immune activation model; a systematic review and meta-analysis of cytokine levels in the offspring
Source: Brain Behav Immun Health. 2020 Dec 19;11:100192. doi: 10.1016/j.bbih.2020.100192 (PMC8474626; doi:10.1016/j.bbih.2020.100192)
Supplement: Multimedia component 1 [file mmc1.doc]

| *Textual ~~deletions~~ and additions to the original protocol published on 1-Nov-2014. All changes were made prior to the extraction phase.* | | |
| --- | --- | --- |
| **Item#** | **Section/topic** | **Description** |
|  | Review title and timescale |  |
| 1. | Title of the review | The maternal poly I:C model, a systematic review and meta-analysis |
| 2. | Original Language | English |
| 3. | Actual or Anticipated start date | 01/02/2015 |
| 4. | Anticipated completion date | ~~31/12/2015~~ 31/07/2020 |
| 5. | Stage of review at time of submission | | Review stage | Started | Completed | | --- | --- | --- | | Preliminary searches | Yes | Yes | | Piloting of the study selection process | Yes | No | | Formal screening of search results against eligibility criteria | No | No | | Data extraction | No | No | | Risk of bias (quality) assessment | No | No | | Data analysis | No | No | |
|  | Review team details |  |
| 6. | Named contact | Lucianne Groenink |
| 7. | Named contact email | l.groenink@uu.nl |
| 8. | Organisational affiliation | Utrecht University |
| 9. | Authors (name, affiliation, contribution) | L Groenink, Utrecht Institute of Pharmaceutical Sciences, Utrecht University, NL  Postdoctoral researcher (to be appointed), Utrecht Institute of Pharmaceutical Sciences, Utrecht University, NL  TBA |
| 10. | Funding sources/ sponsors | ZonMw project MKMD 114024106, NWO-TTW project 16518. |
| 11. | Conflicts of Interest | None to declare |
| 12. | External collaborators | Z Bahor, CAMARADES, University of Edinburgh, UK  M Macleod, CAMARADES, University of Edinburgh, UK  Rob de Vries, SYRCLE, Radboud University Medical Center, NL |
| 13. | Date of protocol registration | March 9, 2015 (protocol overall review) |
|  | Background |  |
| 6. | What is already known about this disease/ model/ intervention? Why is it important to do this review? | *Mental health and infection during pregnancy*  There is a growing body of evidence suggesting that the prenatal environment plays an important role in shaping neurocognitive development (Bale 2014; Brown 2011). Epidemiological studies have shown that disturbance of this environment by infections, but also specific inflammatory factors increases the risk of neuropsychiatric disorders in the offspring (Abdallah 2013; Atladottir 2010; Brown 2004, 2014; Brown and Patterson 2011; Croen 2008). Furthermore, it was shown in animals that prenatal exposure to specific pro-inflammatory factors, in the absence of viral or bacterial infection, is sufficient to cause long-lasting neurocognitive dysfunction (Meyer 2013; Ratyanake 2012, 2013]. These findings indicate that a common activation of the maternal immune system, rather than a direct infection of the fetus, underlies these behavioural changes after prenatal infection. How activation of the maternal immune system affects fetal development is not entirely clear yet. During maternal immune activation, pro-inflammatory cytokines are present in maternal serum, plasma, placenta and amniotic fluid (Green 2014), as well as in fetal plasma and brain (Ratyanake 2013]. These observed increases in pro-inflammatory cytokines in the fetus are probably due to their transfer from the maternal circulation across the placenta (Ashdown 2006; Dahlgren 2006) or alternatively result from fetal uptake of maternal cytokines in the amniotic fluid (Oskvig 2012). Importantly, maternal immune activation may have lasting effects on the immune system in the offspring (Meyer 2012).  Of note, the immune system is not only involved in host defense against pathogens, but is also crucial for certain neurodevelopmental processes, including neurogenesis and pruning of neuronal dendritic spines (Deverman and Patterson 2009; Harry 2013). These processes are partly mediated and regulated by the interaction between specific neurons and glia cells, including both microglia, the resident immune cells of the brain, and astrocytes.  An approach frequently applied in the study of long-term effects of maternal infection during pregnancy in experimental animals is the poly(I:C) model. Administration of Poly(I:C) (polyriboinosinic-polyribocytidilic acid), a synthetic double-stranded RNA, mimics the acute-phase inflammatory response to viral infection (for review Meyer 2012). Maternal poly(I:C) injection during pregnancy alters expression of certain cytokines in the fetal and adult brain of the offspring (Garaya 2013). The expression pattern of these cytokines is related to cognitive, behavioural and neurochemical changes in later life (Ashdown 2006; Bilbo 2006; Meyer 2012; Samuelsson 2006; Ratyanake 2013).  The aim of this review and synthesis of pre-clinical evidence is to systematically assess the effect of maternal poly I:C treatment on ~~behavior and~~ the immune system in the offspring and to gain a deeper understanding of mechanisms that may contribute to the development of ~~the behavioural~~ these changes. |
|  | Objectives of this SR |  |
| 7. | Specify the disease / health problem of interest | Neurodevelopmental deficits in the offspring, associated with maternal viral infection |
| 8. | Specify the population /species studied | All animal species (mammals) |
| 9. | Specify the intervention/exposure | Poly I:C injection(s) during any stage of pregnancy |
| 10. | Specify the control population | - For model characterizing studies a suitable control is an animal of which the mother has not been exposed to poly I:C, but has received a sham equivalent.  - For treatment intervention studies aimed at prevention a  suitable control is recognized as an animal of which the  mother has had the same exposure to poly I:C as those that  are given a treatment, but has received a sham equivalent  of the intervention tested.  - For treatment intervention studies aimed at reducing  symptoms a suitable control is recognized as an animal  which has had the same exposure to poly I:C as those that  are given a treatment, but has received a sham equivalent  of the intervention tested. |
| 11. | Specify the outcome measures | ~~Behavioural, anatomical,~~ immunological, ~~electrophysiological and neurochemical~~ outcomes |
| 12. | State your research question (based on point 7-11) | ~~1. What is the effect of maternal poly I:C treatment on behaviour in the offspring?~~  2. What is the effect of maternal poly I:C treatment on immunological~~, anatomical, neurochemical and electrophysiological~~ measures in the offspring?  ~~3. What is the effect of treatment intervention on~~  ~~maternal poly I:C induced changes?~~  ~~4. How do the effects of maternal poly I:C on~~  ~~biological measures (pt 2) relate to the behavioural~~  ~~changes?~~  5. How do these experimental models compare in  terms of efficiency in modelling the human  condition?  ~~6. How well do results in this domain translate to~~  ~~results in human studies especially in terms of~~  ~~predicting drug efficacy?~~  7. What are the most commonly used study characteristics of the Poly(I:C) model? |
|  | Methods: |  |
|  | Search and study identification |  |
| 13. | Identify literature databases to search (*e.g.* Pubmed, Embase, Web of science) | Pubmed  Web of Science  SCOPUS  EMBASE  Other, namely [type here]  Specific journal(s), namely [type here]  Papers only retrieved in Embase will be labelled as such |
| 14. | Define electronic search strategies (*e.g.* use the [step by step search guide [1]](http://www.ncbi.nlm.nih.gov/pmc/articles/PMC3265183/pdf/LA-11-087.pdf) and animals search filters [[2,](http://www.ncbi.nlm.nih.gov/pmc/articles/PMC3104815/pdf/LA-09-117.pdf) [3](http://www.ncbi.nlm.nih.gov/pmc/articles/PMC3175570/pdf/LA-11-056.pdf)]) | See Page 10-13. |
| 15. | Identify other sources for study identification | Reference lists of included studies  Books  Reference lists of relevant reviews  Conference proceedings, namely [type here]  Contacting authors/ organisations, namely [type here]  Other, namely [type here] |
| 16. | Define search strategy for these other sources | NA |
|  | Study selection procedure |  |
| 17. | Define screening phases (*e.g.* pre-screening based on title/abstract, full text screening, both) | 1. Pre-screening based on title and abstract 2. Full-text screening ~~{{simultaneously performed with data extraction}}~~ to decide |
| 18. | Specify number of observers per screening phase | Two independent observers for each screening phase |
|  | Study selection criteria. Define all inclusion and exclusion criteria based on: |  |
| 19. | Criteria relating to study design | Inclusion criteria: primary research articles  Exclusion criteria: case reports, human studies, letters or comments, reviews (not included, but check for primary data) and conference or seminar abstracts without data, or instances where data being referred to is not clear from publication and studies where there is no appropriate control group. |
| 20. | Type of animals/ population (*e.g.* age, gender, disease model) | Inclusion criteria:   - *in vivo*, poly I:C administration to pregnant dams (with suitable control), with behavioural or biological read-outs being assessed in the offspring (both pre- and postnatally). - ~~Includes genetically modified animals~~ - using animals of all ages, sex and species.   Exclusion criteria:   - genetically modified animals - human, *ex vivo*, *in vitro* studies - administration of poly I:C other than to pregnant dams |
| 21. | Type of intervention (*e.g.* dosage, timing, frequency) | Inclusion criteria:   - all types of studies which characterize the effect of maternal poly I:C on the offspring regardless whether drug is being tested - all types of studies that test a treatment intervention (with suitable control) in the maternal poly I:C model regardless of dosage, time, frequency   Exclusion criteria: |
| 22. | Outcome measures | Inclusion criteria: studies measuring   - ~~Behavioural outcomes~~ - Immunological outcomes - ~~Anatomical outcomes~~ - ~~Electrophysiological outcomes~~ - ~~Neurochemical outcomes~~   Exclusion criteria:   - any other measures (e.g. metabolic outcomes, genetic analyses, proteomics). - Outcome measures assessed in dams. |
| 23. | Language restrictions | Inclusion criteria: all languages  Exclusion criteria: none |
| 24. | Publication date restrictions | Inclusion criteria: all dates  Exclusion criteria: none |
| 25. | Other | NA |
| 26. | Sort and prioritize your exclusion criteria per selection phase | Selection phase: screening on basis of title and abstract   1. Not a primary research article *(review*, comment, editorial, letter to the editor) 2. Study in humans 3. *In vitro, ex vivo* model 4. No poly I:C administered to pregnant dams 5. No appropriate outcome measure (behavioural, anatomical, electrophysiological, neurochemical)   Selection phase: full text screening   1. Criteria above (1 to 5) 2. Absence of appropriate control group 3. Absence of data or inability to retrieve data from paper required for meta-analysis (*n* number, effect size, variance) |
|  | Study characteristics to be extracted (for assessment of external validity, reporting quality) |  |
| 27. | Study meta-data (*e.g.* authors, year) | Authors, Year, Title, Journal Name |
| 28. | Study design characteristics (*e.g.* experimental groups, number of animals) | Number of animals in control and experimental groups (if *n* numbers are given as a range, the most conservative estimate will be extracted) |
| 29. | Animal model characteristics (*e.g.* species, gender, disease induction) | - Species  - Strain  - Sex  - Age  - Weight  - Disorder model (schizophrenia/ psychosis, other)  - Method of disorder induction  Dosage of poly I:C  Mode of delivery  Time (embryonal day) of polyI:C administration  Length of polyI:C administration  Time between induction and outcome measurement  - Sham group  - Co-morbidities (including double hit)  - Whether genetically modified |
| 30. | Intervention characteristics (*e.g.* intervention, timing, duration) | ~~- Treatment tested~~  ~~- Dosage of drug tested~~  ~~- Mode of delivery~~  ~~- Time of treatment administration~~  ~~- Length of treatment~~  ~~- Time between treatment and outcome measurement~~ |
| 31. | Outcome measures | - Type of immunological outcome measurement  ~~- For each outcome: mean, SD or SEM and n for both~~  ~~control and experimental groups~~  ~~- Where a single control group serves multiple treatment~~  ~~groups, the size of the control group used in the metaanalysis will be adjusted by dividing it by the number of treatment groups it serves.~~ |
| 32. | Other (*e.g.* drop-outs) | - Number of excluded animals and reason for exclusion |
|  | Risk of bias assessment (internal validity) |  |
| 33. | Define criteria to assess the internal validity of included studies (*e.g.* selection, performance, detection and attrition bias) | 1. Randomisation:    1. Sequence generation (selection bias)    2. Random housing (performance bias)    3. Random outcome assessment (detection bias) 2. Blinded conduct of experiment    1. Allocation concealment (selection bias)    2. Blinding (performance bias) 3. Blinded assessment of outcome    1. Blinding (detection bias) 4. Statement of inclusion and exclusion criteria    1. Incomplete outcome data (attrition bias)    2. Baseline characteristics (selection bias) 5. ~~Sample size calculation~~ 6. ~~Statement of possible conflict of interest~~ 7. ~~Statement of compliance with animal welfare regulations~~ 8. Availability of study protocol    1. Selective outcome reporting (reporting bias) 9. Other sources of bias: unit-of-analysis errors and design-specific bias   (Hooijmans, C. R., et al. (2014). "SYRCLE's risk of bias tool for animal studies." BMC Med Res Methodol 14: 43.)  Risk of bias will be assessed by two independent researchers |
|  | Collection of outcome data |  |
| 34. | For each outcome measure, define the type of data to be extracted (*e.g.* continuous/ dichotomous, unit of measurement) | ~~- Behavioural outcomes: continuous~~  - Immunological outcomes: continuous  ~~- Anatomical outcomes: continuous~~  ~~- Electrophysiological outomes: continuous~~  ~~- Neurochemical outcomes: continuous~~  - Descriptive outcomes (significantly increased/no effect/significantly decreased/non-detectable)  - Mean, SD or SEM and *n* numbers will be extracted for all outcome measures for both experimental and control groups.  - Where a single control group serves multiple treatment groups, the size of the control group used in the meta-analysis will be adjusted by dividing it by the number of treatment groups it serves. |
| 35. | Methods for data extraction/retrieval (*e.g.* extraction from graphs, contacting authors) | 1. Extraction of numerical data from text of publication 2. In studies where data is only presented graphically, the software Universal Desktop Ruler will be used to extract numerical data. 3. If any data missing: contact authors 4. In the absence of a response from authors, data will be excluded from analysis |
|  | Data analysis/synthesis |  |
| 36. | Specify how you are planning to combine/compare the data (*e.g.* descriptive summary, meta-analysis) | Descriptive summary of all immunological data.  Meta-analysis for cytokine protein levels when available from at least 10 independent studies, with subgroup analyses. |
| 37. | Specify how the decision as to whether a meta-analysis is appropriate will be made | A global search on maternal poly I:C yielded 150 papers. Based on previous systematic reviews we expect to include about 10% of the overall dataset. Potential sources of heterogeneity will be examined through the performance of a meta-analysis with the aggregated data from studies. |
|  | If a meta-analysis seems feasible/sensible: |  |
|  | Primary outcome measure | Estimate of pooled effect size for ~~behavioural~~ immunological outcomes |
|  | Secondary outcome measures | 1. ~~Estimate of pooled effect sizes for each behavioural outcome reported in 5 experiments or more~~ 2. ~~Estimate of pooled effect sizes for immunological outcomes, and for each immunological outcome reported in 5 experiments or more~~ 3. ~~Estimate of pooled effect sizes for anatomical outcomes, and for each anatomical outcome reported in 5 experiments or more~~ 4. ~~Estimate of pooled effect sizes for electrophysiological outcomes, and for each electrophysiological outcome reported in 5 experiments or more~~ 5. ~~Estimate of pooled effect sizes for neurochemical outcomes, and for each neurochemical outcome reported in 5 experiments or more~~ 6. ~~Estimates of pooled effect size for drug efficacy for each category of outcome~~ 7. ~~Estimates of pooled effect sizes for each drug class reported in 5 experiments or more~~ 8. Subgroup analyses as described in (41) below |
|  | Tertiary outcome measures | 1. ~~Estimates of pooled efficacy for each drug for each category of outcome measure~~ 2. ~~Sample size curves for each outcome measure based on observed variance (median, and upper and lower interquartile range boundaries~~ 3. ~~Inter-observer agreement for the application of the inclusion and exclusion criteria~~ |
| 38. | Specify the effect measure to be used (*e.g.* mean difference, standardized mean difference, risk ratio, odds ratio) | ~~For outcome measures where the performance of a normal animal is known or can be inferred in at least 80% of experiments we will use normalised mean difference meta-analysis as the primary outcome, with standardised mean difference as a sensitivity analysis. Where the performance of a normal animal is known or can be inferred in less than 80% of experiments we will use standardised mean difference meta-analysis as the primary outcome, with normalised mean difference as a sensitivity analysis.~~  ~~Within each outcome domain (behavioural, anatomical, neurochemical, electrophysiological, immunological) multiple outcomes from experimental cohorts will be combined (nested) using fixed random effects meta-analysis~~  We will use standardised mean differences (SMD) as effect size measure because of the varying units of measurements and species included. |
| 39. | Specify statistical model of analysis (*e.g.* random or fixed effects model) | Random effects model (because of expected heterogeneity) |
| 40. | Specify statistical methods to assess heterogeneity (*e.g.* I^2^, Q) | ~~Cochrane’s Q for partitioning of heterogeneity;~~ I^2^ to report heterogeneity |
| 41. | Specify which study characteristics will be examined as potential source of heterogeneity (subgroup analysis) | Differences between the following subgroups will be assessed (when at least 5 comparisons from 3 independent studies can be made):  ~~Quality assessment subgroups~~  ~~- each individual quality item~~  ~~- Number of study quality checklist items scored~~  Study characteristics assessment subgroups  - Species of animals used  ~~- Strain of animals used~~  ~~- Gender of animals used~~  ~~- Poly I:C dosage~~  - Time (embryonic stage) of poly I:C challenge  - Age of outcome assessment  - Region of interest  ~~- Route of poly I:C administration~~  ~~- In drug studies, the treatment given~~  ~~- Time treatment is given (pre- vs. post-model induction~~  ~~- Presence of co-treatments (stress, immunological, genetic, pharmacological)~~ |
| 42. | Statistical method to assess differences between subgroups and correction for multiple testing | For between subgroup analyses, each subgroup will be treated as a separate study. Summary effects will be compared using a t-test, with Holm-Bonferroni correction for multiple testing.  ~~We will use meta-regression as the primary measure to assess differences between subgroups, with partitioning of heterogeneity used as a sensitivity analysis.~~  ~~In addition, we will perform meta-regression of immunological outcome versus behavioural outcome; one data point for each cohort of animals where both have been measured~~ |
| 43. | Specify the method for assessment of risk of publication bias | Funnel plot assessment by visual inspection of asymmetry (plotting SMD *vs* 1/√n) ~~Egger’s regression, and trim and fill analysis. These will be conducted independently~~ for each outcome measure using non-nested data. |

**Search strings for PubMed and EMBASE (Elsevier)**

PuBMed

A.

(("Poly I:C"[tiab] OR "Poly(I:C)"[tiab] OR "Poly (I:C)"[tiab] OR "Poly I-C"[Mesh] OR "Poly(IC)"[tiab] OR "Polyinosinic-polycytidylic acid"[tiab] OR "Polyriboinosinic polyribocytidylic acid"[tiab] OR "polyriboinosinic:polyribocytidylic acid"[tiab] OR "polyriboinosinic-polyribocytidylic acid"[tiab])

B.

("Maternal exposure"[Mesh] OR "maternal"[tiab] OR "mother"[tiab] OR "prenatal"[tiab] OR "pregnancy"[tiab] OR "pregnant"[tiab] OR "in utero"[tiab] OR "intrauterine"[tiab] OR "fetal"[tiab] OR "maternal immune activation"[tiab]))

C.

(("animal experimentation"[MeSH Terms] OR "models, animal"[MeSH Terms] OR "invertebrates"[MeSH Terms] OR "Animals"[Mesh:noexp] OR "animal population groups"[MeSH Terms] OR "chordata"[MeSH Terms:noexp] OR "chordata, nonvertebrate"[MeSH Terms] OR "vertebrates"[MeSH Terms:noexp] OR "amphibians"[MeSH Terms] OR "birds"[MeSH Terms] OR "fishes"[MeSH Terms] OR "reptiles"[MeSH Terms] OR "mammals"[MeSH Terms:noexp] OR "primates"[MeSH Terms:noexp] OR "artiodactyla"[MeSH Terms] OR "carnivora"[MeSH Terms] OR "cetacea"[MeSH Terms] OR "chiroptera"[MeSH Terms] OR "elephants"[MeSH Terms] OR "hyraxes"[MeSH Terms] OR "insectivora"[MeSH Terms] OR "lagomorpha"[MeSH Terms] OR "marsupialia"[MeSH Terms] OR "monotremata"[MeSH Terms] OR "perissodactyla"[MeSH Terms] OR "rodentia"[MeSH Terms] OR "scandentia"[MeSH Terms] OR "sirenia"[MeSH Terms] OR "xenarthra"[MeSH Terms] OR "haplorhini"[MeSH Terms:noexp] OR "strepsirhini"[MeSH Terms] OR "platyrrhini"[MeSH Terms] OR "tarsii"[MeSH Terms] OR "catarrhini"[MeSH Terms:noexp] OR "cercopithecidae"[MeSH Terms] OR "hylobatidae"[MeSH Terms] OR "hominidae"[MeSH Terms:noexp] OR "gorilla gorilla"[MeSH Terms] OR "pan paniscus"[MeSH Terms] OR "pan troglodytes"[MeSH Terms] OR "pongo pygmaeus"[MeSH Terms]) OR ((animals[tiab] OR animal[tiab] OR mice[Tiab] OR mus[Tiab] OR mouse[Tiab] OR murine[Tiab] OR woodmouse[tiab] OR rats[Tiab] OR rat[Tiab] OR murinae[Tiab] OR muridae[Tiab] OR cottonrat[tiab] OR cottonrats[tiab] OR hamster[tiab] OR hamsters[tiab] OR cricetinae[tiab] OR rodentia[Tiab] OR rodent[Tiab] OR rodents[Tiab] OR pigs[Tiab] OR pig[Tiab] OR swine[tiab] OR swines[tiab] OR piglets[tiab] OR piglet[tiab] OR boar[tiab] OR boars[tiab] OR "sus scrofa"[tiab] OR ferrets[tiab] OR ferret[tiab] OR polecat[tiab] OR polecats[tiab] OR "mustela putorius"[tiab] OR "guinea pigs"[Tiab] OR "guinea pig"[Tiab] OR cavia[Tiab] OR callithrix[Tiab] OR marmoset[Tiab] OR marmosets[Tiab] OR cebuella[Tiab] OR hapale[Tiab] OR octodon[Tiab] OR chinchilla[Tiab] OR chinchillas[Tiab] OR gerbillinae[Tiab] OR gerbil[Tiab] OR gerbils[Tiab] OR jird[Tiab] OR jirds[Tiab] OR merione[Tiab] OR meriones[Tiab] OR rabbits[Tiab] OR rabbit[Tiab] OR hares[Tiab] OR hare[Tiab] OR diptera[Tiab] OR flies[Tiab] OR fly[Tiab] OR dipteral[Tiab] OR drosphila[Tiab] OR drosophilidae[Tiab] OR cats[Tiab] OR cat[Tiab] OR carus[Tiab] OR felis[Tiab] OR nematoda[Tiab] OR nematode[Tiab] OR nematoda[Tiab] OR nematode[Tiab] OR nematodes[Tiab] OR sipunculida[Tiab] OR dogs[Tiab] OR dog[Tiab] OR canine[Tiab] OR canines[Tiab] OR canis[Tiab] OR sheep[Tiab] OR sheeps[Tiab] OR mouflon[Tiab] OR mouflons[Tiab] OR ovis[Tiab] OR goats[Tiab] OR goat[Tiab] OR capra[Tiab] OR capras[Tiab] OR rupicapra[Tiab] OR chamois[Tiab] OR haplorhini[Tiab] OR monkey[Tiab] OR monkeys[Tiab] OR anthropoidea[Tiab] OR anthropoids[Tiab] OR saguinus[Tiab] OR tamarin[Tiab] OR tamarins[Tiab] OR leontopithecus[Tiab] OR hominidae[Tiab] OR ape[Tiab] OR apes[Tiab] OR pan[Tiab] OR paniscus[Tiab] OR "pan paniscus"[Tiab] OR bonobo[Tiab] OR bonobos[Tiab] OR troglodytes[Tiab] OR "pan troglodytes"[Tiab] OR gibbon[Tiab] OR gibbons[Tiab] OR siamang[Tiab] OR siamangs[Tiab] OR nomascus[Tiab] OR symphalangus[Tiab] OR chimpanzee[Tiab] OR chimpanzees[Tiab] OR prosimians[Tiab] OR "bush baby"[Tiab] OR prosimian[Tiab] OR bush babies[Tiab] OR galagos[Tiab] OR galago[Tiab] OR pongidae[Tiab] OR gorilla[Tiab] OR gorillas[Tiab] OR pongo[Tiab] OR pygmaeus[Tiab] OR "pongo pygmaeus"[Tiab] OR orangutans[Tiab] OR pygmaeus[Tiab] OR lemur[Tiab] OR lemurs[Tiab] OR lemuridae[Tiab] OR horse[Tiab] OR horses[Tiab] OR pongo[Tiab] OR equus[Tiab] OR cow[Tiab] OR calf[Tiab] OR bull[Tiab] OR chicken[Tiab] OR chickens[Tiab] OR gallus[Tiab] OR quail[Tiab] OR bird[Tiab] OR birds[Tiab] OR quails[Tiab] OR poultry[Tiab] OR poultries[Tiab] OR fowl[Tiab] OR fowls[Tiab] OR reptile[Tiab] OR reptilia[Tiab] OR reptiles[Tiab] OR snakes[Tiab] OR snake[Tiab] OR lizard[Tiab] OR lizards[Tiab] OR alligator[Tiab] OR alligators[Tiab] OR crocodile[Tiab] OR crocodiles[Tiab] OR turtle[Tiab] OR turtles[Tiab] OR amphibian[Tiab] OR amphibians[Tiab] OR amphibia[Tiab] OR frog[Tiab] OR frogs[Tiab] OR bombina[Tiab] OR salientia[Tiab] OR toad[Tiab] OR toads[Tiab] OR "epidalea calamita"[Tiab] OR salamander[Tiab] OR salamanders[Tiab] OR eel[Tiab] OR eels[Tiab] OR fish[Tiab] OR fishes[Tiab] OR pisces[Tiab] OR catfish[Tiab] OR catfishes[Tiab] OR siluriformes[Tiab] OR arius[Tiab] OR heteropneustes[Tiab] OR sheatfish[Tiab] OR perch[Tiab] OR perches[Tiab] OR percidae[Tiab] OR perca[Tiab] OR trout[Tiab] OR trouts[Tiab] OR char[Tiab] OR chars[Tiab] OR salvelinus[Tiab] OR "fathead minnow"[Tiab] OR minnow[Tiab] OR cyprinidae[Tiab] OR carps[Tiab] OR carp[Tiab] OR zebrafish[Tiab] OR zebrafishes[Tiab] OR goldfish[Tiab] OR goldfishes[Tiab] OR guppy[Tiab] OR guppies[Tiab] OR chub[Tiab] OR chubs[Tiab] OR tinca[Tiab] OR barbels[Tiab] OR barbus[Tiab] OR pimephales[Tiab] OR promelas[Tiab] OR "poecilia reticulata"[Tiab] OR mullet[Tiab] OR mullets[Tiab] OR seahorse[Tiab] OR seahorses[Tiab] OR mugil curema[Tiab] OR atlantic cod[Tiab] OR shark[Tiab] OR sharks[Tiab] OR catshark[Tiab] OR anguilla[Tiab] OR salmonid[Tiab] OR salmonids[Tiab] OR whitefish[Tiab] OR whitefishes[Tiab] OR salmon[Tiab] OR salmons[Tiab] OR sole[Tiab] OR solea[Tiab] OR "sea lamprey"[Tiab] OR lamprey[Tiab] OR lampreys[Tiab] OR pumpkinseed[Tiab] OR sunfish[Tiab] OR sunfishes[Tiab] OR tilapia[Tiab] OR tilapias[Tiab] OR turbot[Tiab] OR turbots[Tiab] OR flatfish[Tiab] OR flatfishes[Tiab] OR sciuridae[Tiab] OR squirrel[Tiab] OR squirrels[Tiab] OR chipmunk[Tiab] OR chipmunks[Tiab] OR suslik[Tiab] OR susliks[Tiab] OR vole[Tiab] OR voles[Tiab] OR lemming[Tiab] OR lemmings[Tiab] OR muskrat[Tiab] OR muskrats[Tiab] OR lemmus[Tiab] OR otter[Tiab] OR otters[Tiab] OR marten[Tiab] OR martens[Tiab] OR martes[Tiab] OR weasel[Tiab] OR badger[Tiab] OR badgers[Tiab] OR ermine[Tiab] OR mink[Tiab] OR minks[Tiab] OR sable[Tiab] OR sables[Tiab] OR gulo[Tiab] OR gulos[Tiab] OR wolverine[Tiab] OR wolverines[Tiab] OR minks[Tiab] OR mustela[Tiab] OR llama[Tiab] OR llamas[Tiab] OR alpaca[Tiab] OR alpacas[Tiab] OR camelid[Tiab] OR camelids[Tiab] OR guanaco[Tiab] OR guanacos[Tiab] OR chiroptera[Tiab] OR chiropteras[Tiab] OR bat[Tiab] OR bats[Tiab] OR fox[Tiab] OR foxes[Tiab] OR iguana[Tiab] OR iguanas[Tiab] OR xenopus laevis[Tiab] OR parakeet[Tiab] OR parakeets[Tiab] OR parrot[Tiab] OR parrots[Tiab] OR donkey[Tiab] OR donkeys[Tiab] OR mule[Tiab] OR mules[Tiab] OR zebra[Tiab] OR zebras[Tiab] OR shrew[Tiab] OR shrews[Tiab] OR bison[Tiab] OR bisons[Tiab] OR buffalo[Tiab] OR buffaloes[Tiab] OR deer[Tiab] OR deers[Tiab] OR bear[Tiab] OR bears[Tiab] OR panda[Tiab] OR pandas[Tiab] OR "wild hog"[Tiab] OR "wild boar"[Tiab] OR fitchew[Tiab] OR fitch[Tiab] OR beaver[Tiab] OR beavers[Tiab] OR jerboa[Tiab] OR jerboas[Tiab] OR capybara[Tiab] OR capybaras[Tiab]) NOT medline[subset]))

~~A + B + C = 203 hits (on February 10, 2015)~~

A + B + C = 373 hits (on August 6, 2019)

EMBASE (Elsevier)

A.

('polyinosinic polycytidylic acid'/exp or 'polyinosinic polycytidylic acid':ab,ti or 'polyinosinicpolycytidylic acid' or 'poly i:c':ab,ti or 'poly(i:c)':ab,ti or 'poly (i:c)':ab,ti or 'poly i-c':ab,ti or 'poly (i-c)':ab,ti or 'poly(ic)':ab,ti or 'polyinosinic-polycytidylic acid':ab,ti or 'polyriboinosinic polyribocytidylic acid':ab,ti or 'polyriboinosinic:polyribocytidylic acid':ab,ti or 'polyriboinosinic-polyribocytidylic acid':ab,ti)

B.

('exposure'/exp and 'maternal' or 'mother':ab,ti or 'prenatal':ab,ti or 'pregnancy':ab,ti or 'pregnant':ab,ti or 'in utero':ab,ti or 'intrauterine':ab,ti or 'fetal':ab,ti or 'maternal immune activation':ab,ti or 'maternal':ab,ti)

C.

('animal experiment'/exp or 'animal model'/exp or 'experimental animal'/exp or 'transgenic animal'/exp or 'male animal'/exp or 'female animal'/exp or 'juvenile animal'/exp or 'animal'/de or 'chordata'/de or 'vertebrate'/de or 'tetrapod'/de or 'fish'/exp or 'amniote'/de or 'amphibia'/exp or 'mammal'/de or 'reptile'/exp or 'sauropsid'/exp or 'therian'/de or 'monotremate'/exp or 'placental mammals'/de or 'marsupial'/exp or 'euarchontoglires'/de or 'afrotheria'/exp or 'boreoeutheria'/exp or 'laurasiatheria'/exp or 'xenarthra'/exp or 'primate'/de or 'dermoptera'/exp or 'glires'/exp or 'scandentia'/exp or 'haplorhini'/de or 'prosimian'/exp or 'simian'/de or 'tarsiiform'/exp or 'catarrhini'/de or 'platyrrhini'/exp or 'ape'/de or 'cercopithecidae'/exp or 'hominid'/de or 'hylobatidae'/exp or 'chimpanzee'/exp or 'gorilla'/exp or 'orang utan'/exp or animal:ab,ti or animals:ab,ti or pisces:ab,ti or fish:ab,ti or fishes:ab,ti or catfish:ab,ti or catfishes:ab,ti or sheatfish:ab,ti or silurus:ab,ti or arius:ab,ti or heteropneustes:ab,ti or clarias:ab,ti or gariepinus:ab,ti or 'fathead minnow':ab,ti or 'fathead minnows':ab,ti or pimephales:ab,ti or promelas:ab,ti or cichlidae:ab,ti or trout:ab,ti or trouts:ab,ti or char:ab,ti or chars:ab,ti or salvelinus:ab,ti or salmo:ab,ti or oncorhynchus:ab,ti or guppy:ab,ti or guppies:ab,ti or millionfish:ab,ti or poecilia:ab,ti or goldfish:ab,ti or goldfishes:ab,ti or carassius:ab,ti or auratus:ab,ti or mullet:ab,ti or mullets:ab,ti or mugil:ab,ti or curema:ab,ti or shark:ab,ti or sharks:ab,ti or cod:ab,ti or cods:ab,ti or gadus:ab,ti or morhua:ab,ti or carp:ab,ti or carps:ab,ti or cyprinus:ab,ti or carpio:ab,ti or killifish:ab,ti or eel:ab,ti or eels:ab,ti or anguilla:ab,ti or zander:ab,ti or sander:ab,ti or lucioperca:ab,ti or stizostedion:ab,ti or turbot:ab,ti or turbots:ab,ti or psetta:ab,ti or flatfish:ab,ti or flatfishes:ab,ti or plaice:ab,ti or pleuronectes:ab,ti or platessa:ab,ti or tilapia:ab,ti or tilapias:ab,ti or oreochromis:ab,ti or sarotherodon:ab,ti or 'common sole':ab,ti or 'dover sole':ab,ti or solea:ab,ti or zebrafish:ab,ti or zebrafishes:ab,ti or danio:ab,ti or rerio:ab,ti or seabass:ab,ti or dicentrarchus:ab,ti or labrax:ab,ti or morone:ab,ti or lamprey:ab,ti or lampreys:ab,ti or petromyzon:ab,ti or pumpkinseed:ab,ti or pumpkinseeds:ab,ti or lepomis:ab,ti or gibbosus:ab,ti or herring:ab,ti or clupea:ab,ti or harengus:ab,ti or amphibia:ab,ti or amphibian:ab,ti or amphibians:ab,ti or anura:ab,ti or salientia:ab,ti or frog:ab,ti or frogs:ab,ti or rana:ab,ti or toad:ab,ti or toads:ab,ti or bufo:ab,ti or xenopus:ab,ti or laevis:ab,ti or bombina:ab,ti or epidalea:ab,ti or calamita:ab,ti or salamander:ab,ti or salamanders:ab,ti or newt:ab,ti or newts:ab,ti or triturus:ab,ti or reptilia:ab,ti or reptile:ab,ti or reptiles:ab,ti or 'bearded dragon':ab,ti or pogona:ab,ti or vitticeps:ab,ti or iguana:ab,ti or iguanas:ab,ti or lizard:ab,ti or lizards:ab,ti or 'anguis fragilis':ab,ti or turtle:ab,ti or turtles:ab,ti or snakes:ab,ti or snake:ab,ti or aves:ab,ti or bird:ab,ti or birds:ab,ti or quail:ab,ti or quails:ab,ti or coturnix:ab,ti or bobwhite:ab,ti or colinus:ab,ti or virginianus:ab,ti or poultry:ab,ti or poultries:ab,ti or fowl:ab,ti or fowls:ab,ti or chicken:ab,ti or chickens:ab,ti or gallus:ab,ti or 'zebra finch':ab,ti or taeniopygia:ab,ti or guttata:ab,ti or canary:ab,ti or canaries:ab,ti or serinus:ab,ti or canaria:ab,ti or parakeet:ab,ti or parakeets:ab,ti or grasskeet:ab,ti or parrot:ab,ti or parrots:ab,ti or psittacine:ab,ti or psittacines:ab,ti or shelduck:ab,ti or tadorna:ab,ti or goose:ab,ti or geese:ab,ti or branta:ab,ti or leucopsis:ab,ti or woodlark:ab,ti or lullula:ab,ti or flycatcher:ab,ti or ficedula:ab,ti or hypoleuca:ab,ti or dove:ab,ti or doves:ab,ti or geopelia:ab,ti or cuneata:ab,ti or duck:ab,ti or ducks:ab,ti or greylag:ab,ti or graylag:ab,ti or anser:ab,ti or harrier:ab,ti or 'circus pygargus':ab,ti or 'red knot':ab,ti or 'great knot':ab,ti or calidris:ab,ti or canutus:ab,ti or godwit:ab,ti or limosa:ab,ti or lapponica:ab,ti or meleagris:ab,ti or gallopavo:ab,ti or jackdaw:ab,ti or corvus:ab,ti or monedula:ab,ti or ruff:ab,ti or philomachus:ab,ti or pugnax:ab,ti or lapwing:ab,ti or peewit:ab,ti or plover:ab,ti or vanellus:ab,ti or swan:ab,ti or cygnus:ab,ti or columbianus:ab,ti or bewickii:ab,ti or gull:ab,ti or chroicocephalus:ab,ti or ridibundus:ab,ti or albifrons:ab,ti or 'great tit':ab,ti or parus:ab,ti or aythya:ab,ti or fuligula:ab,ti or streptopelia:ab,ti or risoria:ab,ti or spoonbill:ab,ti or platalea:ab,ti or leucorodia:ab,ti or blackbird:ab,ti or turdus:ab,ti or merula:ab,ti or 'blue tit':ab,ti or cyanistes:ab,ti or pigeon:ab,ti or pigeons:ab,ti or columba:ab,ti or pintail:ab,ti or anas:ab,ti or starling:ab,ti or sturnus:ab,ti or owl:ab,ti or 'athene noctua':ab,ti or pochard:ab,ti or ferina:ab,ti or cockatiel:ab,ti or nymphicus:ab,ti or hollandicus:ab,ti or skylark:ab,ti or alauda:ab,ti or tern:ab,ti or sterna:ab,ti or teal:ab,ti or crecca:ab,ti or oystercatcher:ab,ti or haematopus:ab,ti or ostralegus:ab,ti or shrew:ab,ti or shrews:ab,ti or sorex:ab,ti or araneus:ab,ti or crocidura:ab,ti or russula:ab,ti or 'european mole':ab,ti or talpa:ab,ti or chiroptera:ab,ti or bat:ab,ti or bats:ab,ti or eptesicus:ab,ti or serotinus:ab,ti or myotis:ab,ti or dasycneme:ab,ti or daubentonii:ab,ti or pipistrelle:ab,ti or pipistrellus:ab,ti or cat:ab,ti or cats:ab,ti or felis:ab,ti or catus:ab,ti or feline:ab,ti or dog:ab,ti or dogs:ab,ti or canis:ab,ti or canine:ab,ti or canines:ab,ti or otter:ab,ti or otters:ab,ti or lutra:ab,ti or badger:ab,ti or badgers:ab,ti or meles:ab,ti or fitchew:ab,ti or fitch:ab,ti or foumart:ab,ti or foulmart:ab,ti or ferrets:ab,ti or ferret:ab,ti or polecat:ab,ti or polecats:ab,ti or mustela:ab,ti or putorius:ab,ti or weasel:ab,ti or weasels:ab,ti or fox:ab,ti or foxes:ab,ti or vulpes:ab,ti or 'common seal':ab,ti or phoca:ab,ti or vitulina:ab,ti or 'grey seal':ab,ti or halichoerus:ab,ti or horse:ab,ti or horses:ab,ti or equus:ab,ti or equine:ab,ti or equidae:ab,ti or donkey:ab,ti or donkeys:ab,ti or mule:ab,ti or mules:ab,ti or pig:ab,ti or pigs:ab,ti or swine:ab,ti or swines:ab,ti or hog:ab,ti or hogs:ab,ti or boar:ab,ti or boars:ab,ti or porcine:ab,ti or piglet:ab,ti or piglets:ab,ti or sus:ab,ti or scrofa:ab,ti or llama:ab,ti or llamas:ab,ti or lama:ab,ti or glama:ab,ti or deer:ab,ti or deers:ab,ti or cervus:ab,ti or elaphus:ab,ti or cow:ab,ti or cows:ab,ti or 'bos taurus':ab,ti or 'bos indicus':ab,ti or bovine:ab,ti or bull:ab,ti or bulls:ab,ti or cattle:ab,ti or bison:ab,ti or bisons:ab,ti or sheep:ab,ti or sheeps:ab,ti or 'ovis aries':ab,ti or ovine:ab,ti or lamb:ab,ti or lambs:ab,ti or mouflon:ab,ti or mouflons:ab,ti or goat:ab,ti or goats:ab,ti or capra:ab,ti or caprine:ab,ti or chamois:ab,ti or rupicapra:ab,ti or leporidae:ab,ti or lagomorpha:ab,ti or lagomorph:ab,ti or rabbit:ab,ti or rabbits:ab,ti or oryctolagus:ab,ti or cuniculus:ab,ti or laprine:ab,ti or hares:ab,ti or lepus:ab,ti or rodentia:ab,ti or rodent:ab,ti or rodents:ab,ti or murinae:ab,ti or mouse:ab,ti or mice:ab,ti or mus:ab,ti or musculus:ab,ti or murine:ab,ti or 'wood mouse':ab,ti or apodemus:ab,ti or rat:ab,ti or rats:ab,ti or rattus:ab,ti or norvegicus:ab,ti or 'guinea pig':ab,ti or 'guinea pigs':ab,ti or cavia:ab,ti or porcellus:ab,ti or hamster:ab,ti or hamsters:ab,ti or mesocricetus:ab,ti or cricetulus:ab,ti or cricetus:ab,ti or gerbil:ab,ti or gerbils:ab,ti or jird:ab,ti or jirds:ab,ti or meriones:ab,ti or unguiculatus:ab,ti or jerboa:ab,ti or jerboas:ab,ti or jaculus:ab,ti or chinchilla:ab,ti or chinchillas:ab,ti or beaver:ab,ti or beavers:ab,ti or 'castor fiber':ab,ti or 'castor canadensis':ab,ti or sciuridae:ab,ti or squirrel:ab,ti or squirrels:ab,ti or sciurus:ab,ti or chipmunk:ab,ti or chipmunks:ab,ti or marmot:ab,ti or marmots:ab,ti or marmota:ab,ti or suslik:ab,ti or susliks:ab,ti or spermophilus:ab,ti or cynomys:ab,ti or cottonrat:ab,ti or cottonrats:ab,ti or sigmodon:ab,ti or vole:ab,ti or voles:ab,ti or microtus:ab,ti or myodes:ab,ti or glareolus:ab,ti or primate:ab,ti or primates:ab,ti or prosimian:ab,ti or prosimians:ab,ti or lemur:ab,ti or lemurs:ab,ti or lemuridae:ab,ti or loris:ab,ti or 'bush baby':ab,ti or 'bush babies':ab,ti or bushbaby:ab,ti or bushbabies:ab,ti or galago:ab,ti or galagos:ab,ti or anthropoidea:ab,ti or anthropoids:ab,ti or simian:ab,ti or simians:ab,ti or monkey:ab,ti or monkeys:ab,ti or marmoset:ab,ti or marmosets:ab,ti or callithrix:ab,ti or cebuella:ab,ti or tamarin:ab,ti or tamarins:ab,ti or saguinus:ab,ti or leontopithecus:ab,ti or 'squirrel monkey':ab,ti or 'squirrel monkeys':ab,ti or saimiri:ab,ti or 'night monkey':ab,ti or 'night monkeys':ab,ti or 'owl monkey':ab,ti or 'owl monkeys':ab,ti or douroucoulis:ab,ti or aotus:ab,ti or 'spider monkey':ab,ti or 'spider monkeys':ab,ti or ateles:ab,ti or baboon:ab,ti or baboons:ab,ti or papio:ab,ti or 'rhesus monkey':ab,ti or macaque:ab,ti or macaca:ab,ti or mulatta:ab,ti or cynomolgus:ab,ti or fascicularis:ab,ti or 'green monkey':ab,ti or 'green monkeys':ab,ti or chlorocebus:ab,ti or vervet:ab,ti or vervets:ab,ti or pygerythrus:ab,ti or hominoidea:ab,ti or ape:ab,ti or apes:ab,ti or hylobatidae:ab,ti or gibbon:ab,ti or gibbons:ab,ti or siamang:ab,ti or siamangs:ab,ti or nomascus:ab,ti or symphalangus:ab,ti or hominidae:ab,ti or orangutan:ab,ti or orangutans:ab,ti or pongo:ab,ti or chimpanzee:ab,ti or chimpanzees:ab,ti or 'pan troglodytes':ab,ti or bonobo:ab,ti or bonobos:ab,ti or 'pan paniscus':ab,ti or gorilla:ab,ti or gorillas:ab,ti or troglodytes:ab,ti)

D.

NOT [conference abstract]/lim

~~A + B + C + D = 224 hits (on February 10, 2015)~~

A + B + C + D = 382 hits (on August 6, 2019)
